# Development of supramolecular anticoagulants with on-demand reversibility

**DOI:** 10.1038/s41587-024-02209-z

**Published:** 2024-04-30

**Authors:** Millicent Dockerill, Daniel J. Ford, Simona Angerani, Imala Alwis, Luke J. Dowman, Jorge Ripoll-Rozada, Rhyll E. Smythe, Joanna S. T. Liu, Pedro José Barbosa Pereira, Shaun P. Jackson, Richard J. Payne, Nicolas Winssinger

**Affiliations:** 1 Department of Organic Chemistry, NCCR Chemical Biology, Faculty of Sciences, University of Geneva, Geneva, Switzerland; 2 School of Chemistry, The University of Sydney, Sydney, New South Wales Australia; 3 Australian Research Council Centre of Excellence for Innovations in Peptide and Protein Science, The University of Sydney, Sydney, New South Wales Australia; 4 Charles Perkins Centre, The University of Sydney, Sydney, New South Wales Australia; 5 Heart Research Institute, Sydney, New South Wales Australia; 6 Instituto de Biologia Molecular e Celular (IBMC), Universidade do Porto, Porto, Portugal; 7 Instituto de Investigação e Inovação em Saúde, Universidade do Porto, Porto, Portugal

**Keywords:** Dynamic combinatorial chemistry, Pharmacodynamics

## Abstract

Drugs are administered at a dosing schedule set by their therapeutic index, and termination of action is achieved by clearance and metabolism of the drug. In some cases, such as anticoagulant drugs or immunotherapeutics, it is important to be able to quickly reverse the drug’s action. Here, we report a general strategy to achieve on-demand reversibility by designing a supramolecular drug (a noncovalent assembly of two cooperatively interacting drug fragments held together by transient hybridization of peptide nucleic acid (PNA)) that can be reversed with a PNA antidote that outcompetes the hybridization between the fragments. We demonstrate the approach with thrombin-inhibiting anticoagulants, creating very potent and reversible bivalent direct thrombin inhibitors (*K*_i_ = 74 pM). The supramolecular inhibitor effectively inhibited thrombus formation in mice in a needle injury thrombosis model, and this activity could be reversed by administration of the PNA antidote. This design is applicable to therapeutic targets where two binding sites can be identified.

## Abstract (web summary)

A supramolecular drug design yields anticoagulants that are rapidly reversible.

## Main

Anticoagulants are critically important therapies for the prevention or reversal of thrombotic events and function by reducing fibrin deposition by inhibiting fibrinogen proteolysis and/or platelet activation^1^. A key target of anticoagulant therapy is the protease thrombin (coagulation factor IIa (FIIa)). The use of anticoagulants is based on a risk–benefit analysis, according to which prevention or reduction of progression of thromboembolic disease outweighs the increased risk of bleeding in adverse events or trauma. Indeed, many anticoagulants, particularly heparin and warfarin^2^, require close clinical monitoring to prevent life-threatening bleeding side effects. Nevertheless, anticoagulant-related bleeding and adverse effects are responsible for an estimated 15% of all emergency hospital visits for adverse drug effects^3^ and, as such, strategies for the reversal of anticoagulation are therefore essential^4^. A common strategy to reverse the effects of anticoagulants is the administration of nonspecific reversal agents, which involves the infusion of coagulation factors designed to overwhelm the effects of circulating anticoagulants^5^. In clinical settings, unfractionated heparin is a useful anticoagulant because protamine sulfate can be used for rapid reversal, but unfractionated heparin requires close monitoring^6,7^. More recently, monoclonal antibodies and recombinant FXa have been developed, which bind to specific small-molecule anticoagulants with high affinity (idarucizumab for dabigatran and andexanet-α for apixaban, edoxaban and rivaroxaban), thus reversing the inhibition of FXa or thrombin^8,9^. Although these approaches are effective, there are limitations for their use, and they are costly.

**Fig. 1:**
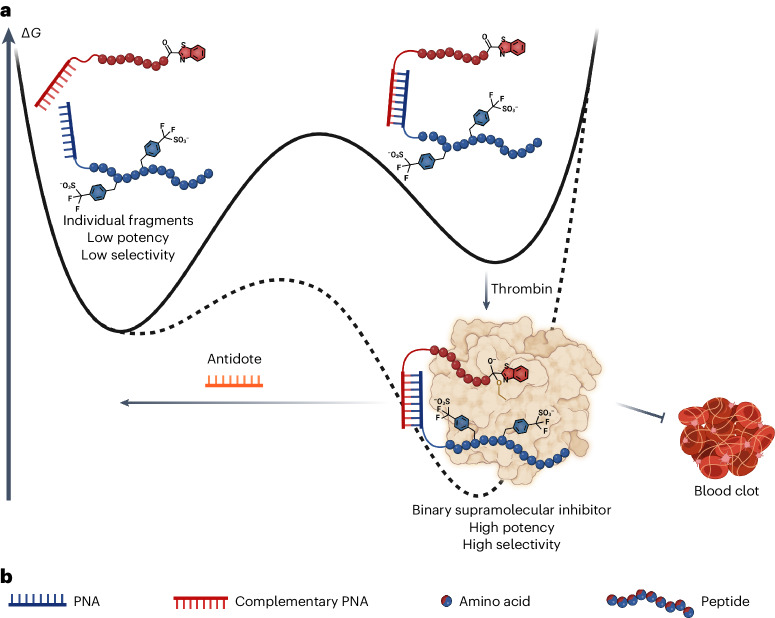
Supramolecular drug with on-demand reversibility. **a**, Assembly of the supramolecular drug is catalyzed by the binding to thrombin, which creates a highly potent and highly selective inhibitor from two compounds with low potency and selectivity. The inhibition of thrombin can be rapidly reversed by the addition of an antidote. ∆*G*, Gibbs free energy change. **b**, Legend of components represented in **a**.

Here, we present a means to generate potent thrombin-inhibiting anticoagulants with on-demand reversibility through programmed supramolecular assembly^10^. Supramolecular entities rely on labile noncovalent interactions and by their very nature are dynamic and reversible in response to specific environmental cues or stimuli by shifting equilibria in the system^11^. These features of supramolecular systems have been elegantly applied to molecular recognition, catalysis, molecular motors, stimuli-responsive polymers and drug discovery and delivery but, to our knowledge, have not been realized for applications in medicinal chemistry and pharmacology^12–16^. Our strategy is based on the ability to link two fragments by a reversible supramolecular interaction, and these two fragments can interact cooperatively with the target at two distinct sites (Fig. 1), with the formation of the active inhibitor instructed by the target. Disruption of the supramolecular interaction linking the two fragments results in a loss of cooperativity and thus of inhibitory activity. Our design of supramolecular thrombin inhibitors made use of binary interactions directed to the active site of thrombin and to exosite II (the so-called heparin binding site), joined together by hybridized peptide nucleic acid (PNA) molecules.

## Results

**Extended Data Fig. 1:**
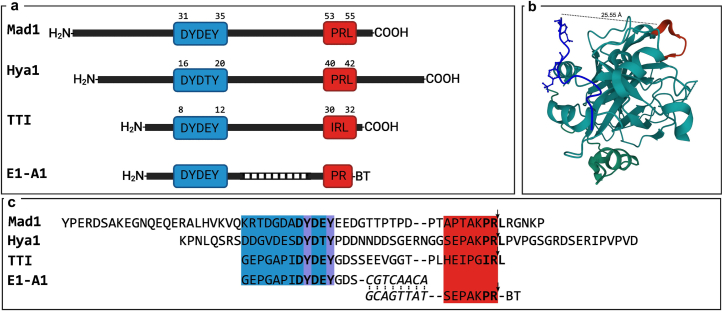
Amino acid sequence alignment of inhibitors from a range of blood-feeding arthropods alongside the supramolecular drug. **a**. Schematic representation of sequences of Madanin1 (Mad1), Hyalomin1 (Hya1) and tsetse fly thrombin inhibitors (TTI) alongside **A1-E1**. **b**. Crystal structure of the tsetse thrombin inhibitor:thrombin complex (PDB 6TKG) showing the distance between the active site and exosite II binding components. **c**. Full sequences aligned. The exosite II sequence alignment is shown on a blue background with the sulfated tyrosine residues highlighted in purple. The active site sequence alignment is shown on a red background with the scissile bond indicated by an arrow. The PNA is written in italics and the benzothiazole reversible covalent warhead is shortened to ‘BT’.

The designed supramolecular assembly was inspired by thrombin inhibitors produced naturally by blood-feeding (hematophagous) organisms, such as leeches, ticks, mosquitoes and flies, which secrete small protein thrombin inhibitors from their salivary glands to facilitate acquisition and digestion of a bloodmeal. These salivary proteins exhibit potent thrombin inhibition by interacting with two distinct binding sites on thrombin, but this activity cannot be easily reversed due to their extremely high affinity for thrombin. At the outset, we focused on hyalomin 1, a 59-residue sulfated protein secreted by the tick *Hyalomma marginatum rufipes* that shares sequence similarity to other tick anticoagulant proteins but is the most potent thrombin inhibitor in the family (*K*_i_ = 5.4 pM). Analysis of the X-ray crystal structures of several of these proteins complexed with thrombin (for example, tick-derived madanin-1 (Protein Data Bank (PDB) 5L6N)^17^ and tsetse thrombin inhibitor (TTI) from the tsetse fly (PDB 6TKG))^18^ together with the thrombin inhibitory data suggested that the potent inhibition exhibited by these molecules was derived from interactions at two loci of thrombin, the active site and exosite II, separated by 20–30 Å (Extended Data Fig. 1a–c). We reasoned that we could leverage an established ketobenzothiazole-containing mechanism-based pan-serine protease inhibitor for active site targeting that forms reversible covalent (hemiketal) intermediates with serine proteases but is not selective for thrombin^19^. For the peptide targeting exosite II, we investigated sequences from several salivary proteins from hematophagous organisms that possess sulfotyrosine residues as a common post-translational modification that has been shown to enhance activity (Extended Data Fig. 1c)^17,18,20,21^. Considering the reported lability of the tyrosine sulfate post-translational modification, we opted for a synthetic analog of the natural modification, namely sulfono(difluoro)methyl-phenylalanine (F_2_Smp)^22^. For the link between the two binding motifs in our supramolecular anticoagulant, we chose to use the synthetic DNA mimetic PNA^23^ based on the tunability of the hybridization dynamics of this molecular class to provide anticoagulant reversibility, its metabolic stability and the compatibility of its chemistry with peptide synthesis^24^.

### Synthesis and characterization of supramolecular inhibitors

**Fig. 2:**
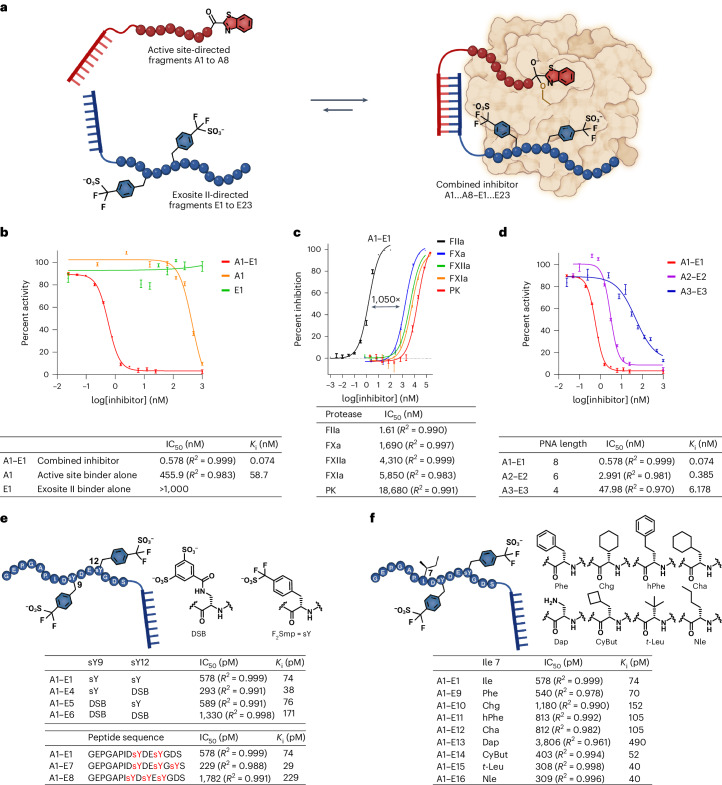
Inhibitor development. **a**, Schematic representation of cooperative dynamic drug assembly. The inhibitors disclosed in this study are composed of two fragments: the active site-directed fragments, which are numbered A1 to A8, and the exosite II-directed fragments, which are numbered E1 to E23. Combination of the two fragments yields a potent inhibitor named as the combination of the two assembled fragments (for example, A1–E1 is the combination of active site fragment A1 and exosite II fragment E1). **b**, Thrombin inhibition data for the combined inhibitor versus the two fragments alone. **c**, Selectivity data for A1–E1 against a panel of common proteases. **d**, Effect of PNA length on inhibition. **e**, Structure–activity relationship data of the exosite II binder by different charge. **f**, Structure–activity relationship data of the exosite II binder by different hydrophobic amino acids instead of isoleucine. For all data, *n* = 3 replicates, with individual data points presented as mean values ± s.d., with the exception of the data in **c** where *n* = 2. Source data

**Extended Data Fig. 2:**
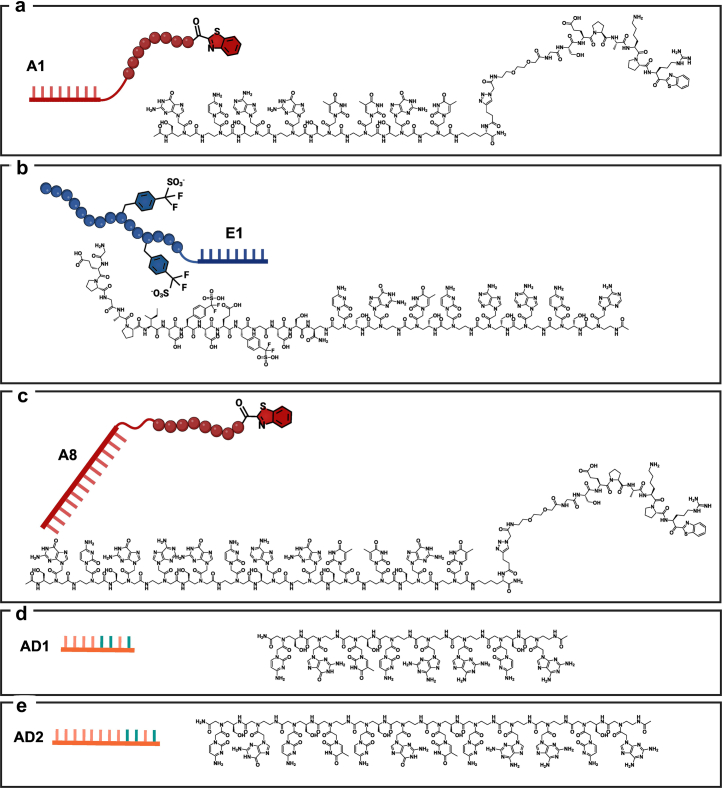
Chemical Structures of main compounds of the study. **a**. Active site-directed inhibitor **A1**, **b**. Exosite II-directed inhibitor **E1**, **c**. Active site-directed inhibitor with 4-mer toehold PNA **A8**, **d**. 8-mer antidote **AD1** and **e**. 12-mer antidote **AD2**.

**Extended Data Fig. 3:**
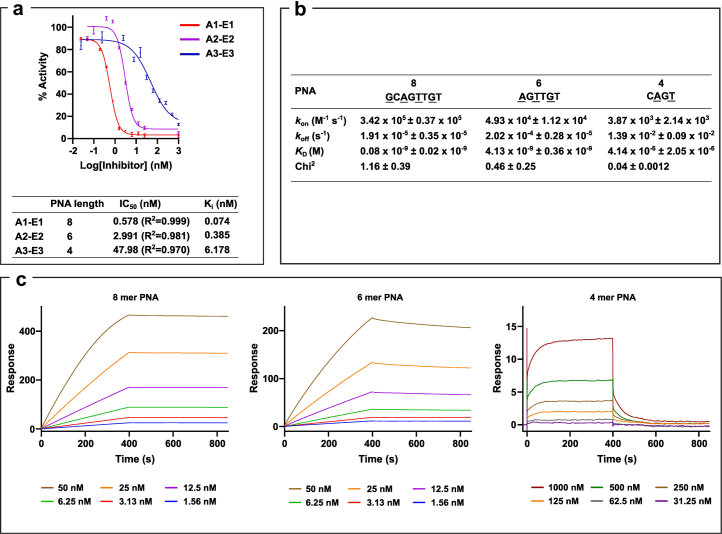
K_D_ of binding between PNAs of different lengths. **a**. Inhibition data for inhibitors with different length of PNA. **b**. SPR data for the binding between 4-, 6-, and 8-mer PNA strands with **ACAACTGC** immobilised via a biotin on a streptavidin coated SPR chip. The PNA sequences are written N to C, with serine-modified monomers underlined. **c**. SPR kinetic curves. For data presented in **a**. and **b**., n = 3 replicates with individual data points presented as mean values +/− SD. Source data

We first used solid-phase synthesis to prepare the mechanism-based active site targeting peptide fragment A (A1, derived from hyalomin 1 fused to a ketobenzothiazole warhead) linked to an 8-mer PNA sequence and fragment E (E1, derived from the exosite II binding region of TTI) linked to the complementary 8-mer PNA (Fig. 2a and Extended Data Fig. 2). Given the known importance of two native negatively charged sulfotyrosine residues for interaction with the heparin binding exosite II in TTI, we incorporated two F_2_Smp residues as stable mimics in fragment E1. Fragment A1 showed moderate inhibitory activity against thrombin (*K*_i_ = 58.7 nM) in a fluorogenic thrombin activity assay, whereas E1 alone possessed no inhibitory activity (Fig. 2b). However, an 800-fold enhancement of activity was observed when both components were mixed together using the 8-mer PNA supramolecular connection, with A1–E1 exhibiting a *K*_i_ of 74 pM (Fig. 2b). This supramolecular inhibitor also gained selectivity for thrombin when tested against a panel of proteases present in the coagulation pathway, including FXa, FXIa, FXIIa and plasma kallikrein (PK) (>1,000-fold; Fig. 2c). It is noteworthy that, like thrombin, the substrate specificities for FXa and FXIa also strongly favor arginine at P1 (refs. ^25,26^), but only thrombin benefits from the bivalent interaction of the supramolecular drug, resulting in greater than 1,000-fold selectivity. To further investigate the supramolecular connectivity between the two fragments, we reduced the length of the PNA from 8-mer to 6-mer or 4-mer while keeping the overall distance equal. This led to a progressive loss of activity (Fig. 2d). However, the assembly composed of the shortest supramolecular linker (4-mer: A3–E3) was still tenfold more potent than the active site inhibitor alone (A1). Together, these data support a cooperative interplay between the supramolecular interaction of the PNA and the inhibition of thrombin through engagement with both the active site and exosite II. The hybridization *K*_D_ of the 4-mer PNA was measured by surface plasmon resonance (SPR) to be 4.14 µM at 25 °C (Extended Data Fig. 3), yet the supramolecular tether still yielded a benefit at concentrations well below the *K*_D_. Cooperativity in inhibition was observed if the equilibrium rebinding of the active site ligand was faster in the supramolecular assembly–enzyme complex than the dissociation of the supramolecular tether. It stands to reason that the longer PNA with slower *k*_off_ yields better cooperativity.

**Extended Data Fig. 4:**
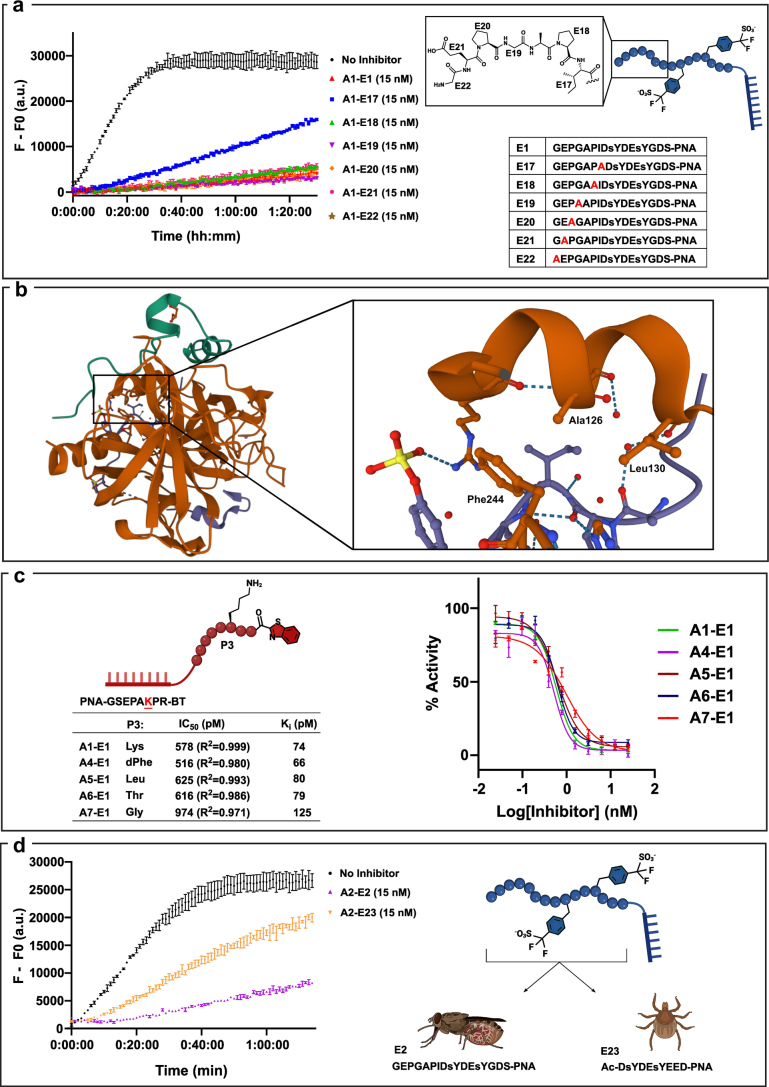
Additional SAR. **a**. Exosite II Ala scan - fluorogenic inhibition assay data for **A1** combined with **E17** to **E22**. **b**. Crystal structure (PDB 6TKG) of the tsetse thrombin inhibitor (TTI) complexed with thrombin. The zoom shows the hydrophobic pocket in which isoleucine is situated. **c**. IC_50_ values for active site binders with modifications in the P3 positions. **d**. Comparison between inhibitors inspired from the exosite II sequence of the tsetse thrombin inhibitor (TTI) and Madanin1 (Mad1, from the *Haemaphysalis longicornis* species) at 15 nM. For data presented in **a., c**. and **d**., n = 3 replicates with individual data points presented as mean values +/− SD. Source data

The use of PNA as a supramolecular tether also provides the opportunity to quickly assemble analogs and perform structure–activity studies because new combinations can be generated by simply mixing the binary ligands. We first explored other stable sulfotyrosine mimics in the exosite II binding fragment by incorporating disulfonic benzoate (DSB) in lieu of F_2_Smp (referred to as sY herein) into the E fragment to generate E4 (sY12 → DSB), E5 (sY9 → DSB) and E6 (sY9,sY12 → DSB) that could be used to form supramolecular assemblies with active site binding fragment A1 by simple mixing (Fig. 2e)^18^. Inclusion of DSB in place of F_2_Smp at position 12 led to a twofold gain of activity (A1–E4; Fig. 2e), but replacement of both F_2_Smp residues with DSB moieties led to a decrease in inhibition (A1–E6; Fig. 2e). The position and number of sulfotyrosine mimics also had a strong impact (A1–E1 versus A1–E7/A1–E8; Fig. 2e). We next performed an alanine scan of the peptide sequence of E1 that targeted exosite II. This revealed an isoleucine residue at position 7 as a hot spot (Extended Data Fig. 4a), an observation consistent with the structure of the TTI–thrombin complex (PDB 6TKG; Extended Data Fig. 4b)^18^, which shows this isoleucine filling a hydrophobic pocket. A moderate (approximately twofold) gain in activity could be achieved with substitution for hydrophobic nonproteinogenic amino acids (for example, *tert*-leucine or norleucine in A1–E15 or A1–E16, respectively; Fig. 2f).

### In vitro evaluation of the supramolecular inhibitor

**Extended Data Fig. 5:**
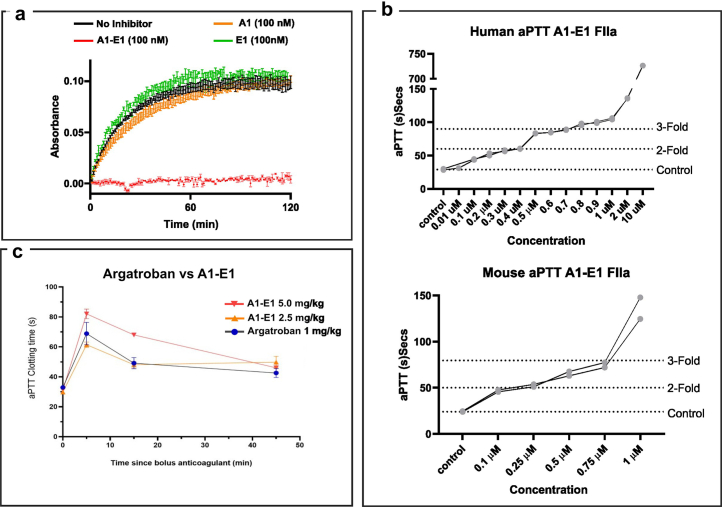
Additional Assays. **a**. Fibrinogen inhibition assay of compounds **A1-E1** and **A1** and **E1** alone. **b**. In vitro aPTT of **A1-E1** in human (top) and mouse (bottom) plasma. n = 2 with both replicates displayed. **c**. Ex-vivo aPTT of **A1-E1** at 0.314 μmol/kg (2.5 mg/kg) and 0.627 μmol/kg (5 mg/kg) versus Argatroban at 1.966 μmol/kg (1 mg/kg). For data presented in **a**., and **c**., n = 3 replicates with individual data points presented as mean values +/− SD. Source data

**Extended Data Fig. 6:**
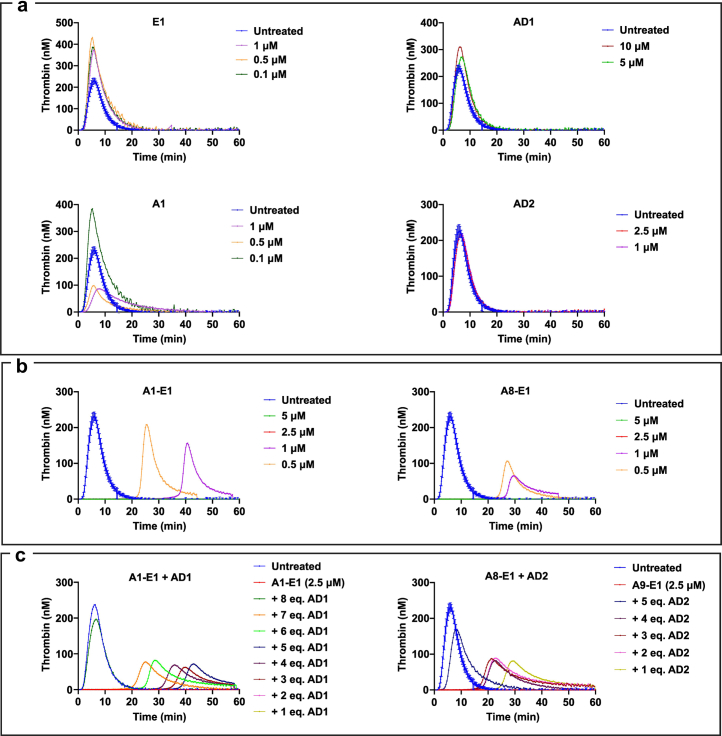
Calibrated Automated Thrombogram (CAT). **a**. **A1**, **E1**, **AD1**, and **AD2** tested alone at a range of concentrations. **b**. Combined inhibitors **A1-E1** and **A8-E1** tested at a range of concentrations. **c**. Combined inhibitors with antidote **A1-E1+AD1** and **A8-E1+AD2** tested at 2.5 μM with a range of antidote concentrations. For all data presented, n = 3 replicates with individual data points presented as mean values +/− SD. Source data

Having established the feasibility of the supramolecular inhibitor concept, we selected A1–E1 as a lead to profile in subsequent biochemical assays and for anticoagulant activity in vitro. To this end, we first investigated the inhibition of fibrinogen proteolysis, whereby A1–E1 exhibited complete inhibition at 100 nM (Extended Data Fig. 5a), whereas A1 or E1 alone was comparable to no inhibitor. Having demonstrated that A1–E1 could prevent fibrinogen proteolysis in vitro, we next turned our attention to an activated partial thromboplastin time (aPTT) assay in both human and mouse plasma. aPTT assays are routine tests performed by physicians and are used as an indicator of the function of coagulation factors in the intrinsic and common pathways. Effective inhibition of thrombin is expected to lengthen the time plasma takes to clot, and a clinically significant increase in clotting is said to be twofold. A1–E1 exhibited a therapeutically significant prolongation of clotting time in both human and mouse plasma at a concentration as low as 250 nM (Extended Data Fig. 5b). We next investigated the effects of A1–E1 on thrombin generation in a calibrated automated thrombogram (CAT). The CAT uses a fluorogenic thrombin substrate, thus allowing measurement of thrombin formation in plasma in real time. This is of particular importance because thrombin generation is a dynamic process; the coagulation cascade has many feedback loops and inhibitory pathways that are all directly or indirectly influenced by the developing thrombin concentration, and thrombin plays a central and pivotal role throughout the whole process. Additionally, and in contrast to aPTT assays, the CAT allows for a large variation in the concentration and character of the trigger used and can therefore be implemented to detect subtle differences between thrombin inhibitors. A1–E1 potently inhibited thrombin activity in both the initiation phase and propagation phase of coagulation and was able to completely inhibit thrombin activity at 2.5 µM (Extended Data Fig. 6).

### In vivo evaluation of the supramolecular inhibitor

**Fig. 3:**
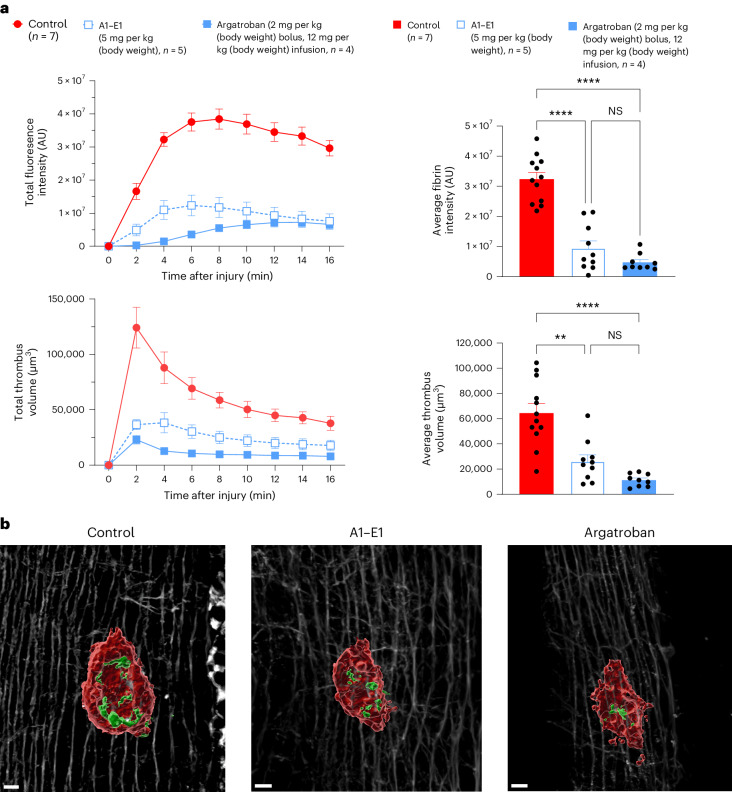
In vivo inhibition of thrombin. **a**, Time course of fibrin fluorescence intensity and total thrombus volume (left) and bar chart quantification of average fibrin intensity and average thrombus volume (right) for control animals (*n* = 7), argatroban-treated animals (*n* = 4; 2 mg per kg (body weight) bolus followed by an infusion of 12 mg per kg (body weight)) and A1–E1-treated animals (*n* = 5; 5 mg per kg (body weight) bolus). Statistical significance between multiple treatment groups was analyzed using an ordinary one-way analysis of variance (ANOVA) with Tukey’s multiple comparisons testing with a single pooled variance. For multiple comparisons testing, the mean of each column was compared to the mean of every other column. Data are presented as mean ± s.e.m., where ‘*n*’ equals the number of independent experiments performed; NS, not significant (*P* = 0.6660 for average fibrin intensity and *P* = 0.5128 for average thrombus volume); **P = 0.0025; ****P < 0.0001; AU, arbitrary units. **b**, Exemplar image of a thrombus 15 min after needle injury without inhibitor (left), with A1–E1 (center) and with argatroban (right). Platelets are shown in red, fibrin is shown in green, and collagen in the background is shown in white; scale bars, 10 μm. Source data

Having determined that our supramolecular anticoagulant potently inhibited thrombin activity and possessed anticoagulant activity in vitro, we next investigated whether A1–E1 would be effective at inhibiting thrombus formation in vivo. To determine a suitable dose for our in vivo efficacy study, we used an ex vivo aPTT assay. Briefly, A1–E1 was administered intravenously to mice at 2.5 or 5 mg per kg (body weight), and blood samples were collected at 5, 15 and 45 min. Clotting times were then measured using a standard aPTT protocol and showed that a single bolus (5 mg per kg (body weight)) was effective at prolonging the aPTT greater than twofold for 30 min (Extended Data Fig. 5c). We next assessed the in vivo efficacy of the supramolecular anticoagulant A1–E1 compared to the standard of care argatroban in a localized needle injury model^27^. This injury leads to both fibrin formation and platelet aggregation in thrombus formation, which were visualized by Alexa 546-conjugated anti-fibrin and DyLight 649-conjugated anti-GP1bβ^28^. Owing to its short half-life in vivo, argatroban was dosed with an intravenous bolus (3.9 μmol kg^–1^ (2 mg per kg (body weight))) followed by an infusion at 24 μmol kg^–1^ over 60 min (12 mg per kg (body weight), total dose of 27.9 μmol kg^–1^). A1–E1 was dosed twice via intravenous bolus at 0.63 μmol kg^–1^ (5 mg per kg (body weight)) 30 min apart (total dose of 1.3 μmol kg^–1^). Both A1–E1 and argatroban showed significant decreases in fibrin formation and thrombus size (Fig. 3). After treatment with the supramolecular anticoagulant A1–E1 followed by injury, we observed near complete inhibition of fibrin deposition at the site of injury compared to control-treated injuries (Fig. 3). We also observed that A1–E1 achieved a similar level of anticoagulation as a bolus infusion of argatroban at the 5 mg per kg (body weight) dosing regimen (Fig. 3). On a molarity basis, A1–E1 yielded comparable results to the standard of care (argatroban) at 24-fold lower drug loading, indicating that the potent inhibitory activity observed in vitro translates in vivo.

### On-demand on/off activity switching in vitro and in vivo

**Fig. 4:**
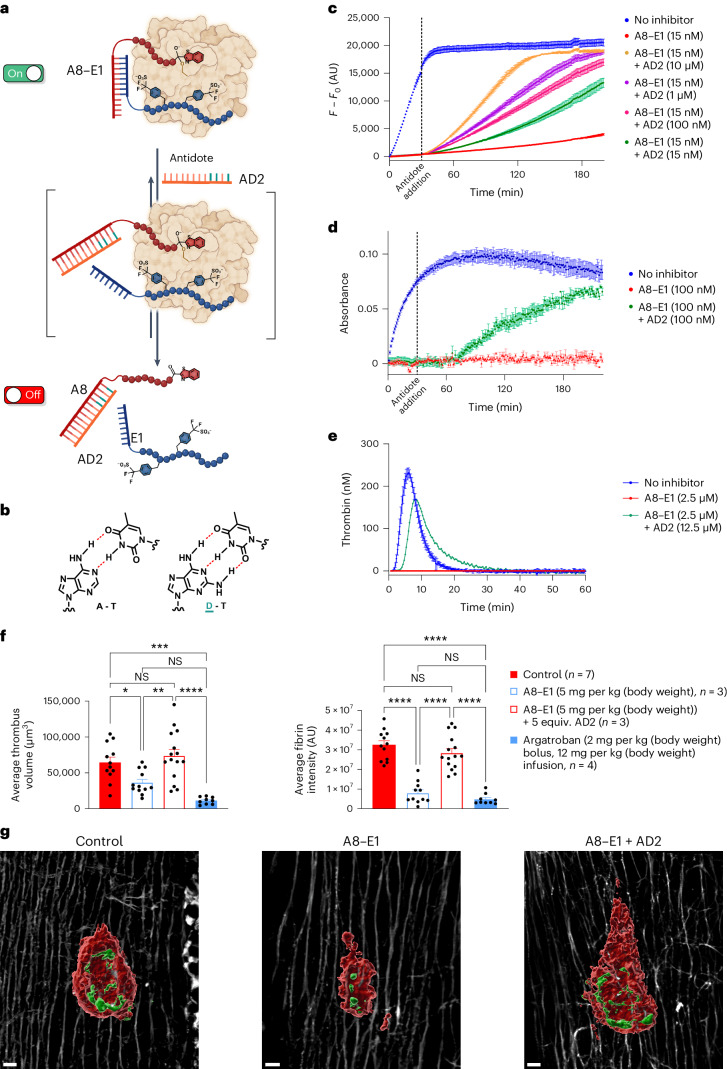
Reversal of thrombin inhibition. **a**, Schematic representation of antidote addition and reversal of inhibition. **b**, Chemical structures of adenine and diaminopurine forming hydrogen bonds with thymine. **c**, Fluorogenic assay data showing the reversal of thrombin inhibition by the addition of different concentrations of antidote after 30 min of inhibition. Data are presented as mean ± s.d., with *n* = 3 replicates. **d**, Fibrinogen assay data showing the reversal of thrombin inhibition by the addition of antidote (1 equiv.) after 30 min of inhibition. Data are presented as mean ± s.d., with *n* = 3 replicates. **e**, CAT of A8–E1 with and without antidote. Data are presented as mean values, with *n* = 3 replicates. **f**, Average fibrin intensity and average thrombus volume for control-treated animals (*n* = 7), argatroban-treated animals (*n* = 4; 2 mg per kg (body weight) bolus followed by an infusion of 12 mg per kg (body weight)), A8–E1-treated animals (*n* = 3; 5 mg per kg (body weight) bolus) and A8–E1 + AD2-treated animals (*n* = 3; 5 mg per kg (body weight) + 5 molar equiv. antidote). Statistical significance between multiple treatment groups was analyzed using an ordinary one-way ANOVA with Tukey’s multiple comparisons testing and a single pooled variance. For multiple comparisons, the mean of each column was compared to the mean of every other column. Data are presented as mean ± s.e.m., where ‘*n*’ equals the number of independent experiments performed. The *P* values for the average thrombus volume data are *P = 0.0412 (control versus A8–E1), *P* = 0.7759 (NS; control versus A8–E1 + AD2), ***P = 0.0001 (control versus argatroban), **P = 0.0021 (A8–E1 versus A8–E1 + AD2), *P* = 0.1208 (NS; A8–E1 versus argatroban) and ****P < 0.0001 (A8–E1 + AD2 versus argatroban). The *P* values for the average fibrin intensity data are *P* = 0.4545 (NS; control versus A8–E1 + AD2), *P* = 0.7333 (NS; A8–E1 versus argatroban) and ****P < 0.0001 (all other comparisons). **g**, Exemplar image of a thrombus 15 min after needle injury without inhibitor (left), with A8–E1 (center) and with A8–E1 + AD2 (right). Platelets are shown in red, fibrin is shown in green, and collagen is shown in white; scale bars, 10 μm. Source data

**Extended Data Fig. 7:**
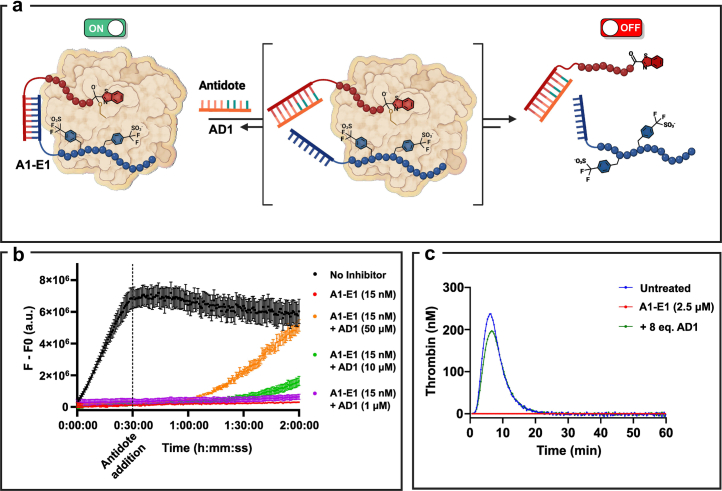
Reversal of thrombin inhibition with an 8 mer PNA antidote. **a**. Schematic representation of antidote addition and reversal of inhibition. **b**. Fluorogenic assay data showing the reversal of thrombin inhibition by addition of different concentrations of antidote (**AD1**) after 30 minutes of inhibition. **c**. Calibrated Automated Thrombogram (CAT) of **A1-E1** with and without antidote (**AD1**). For data presented in **b**., and **c**., n = 3 replicates with individual data points presented as mean values +/− SD. Source data

Having established promising in vivo efficacy for our supramolecular inhibitor, we turned our attention to investigate the ability to reverse the anticoagulant activity with an antidote. Given the non-covalent nature of the supramolecular linker between the active site and exosite II binding entities, we rationalized that the inhibition could be disrupted by competing for the hybridization. To favor the equilibrium toward the dissociation of the binary fragments, the competitor PNA was designed to incorporate diaminopurines instead of adenine because oligomers containing diaminopurines form more stable duplexes with their complementary strand than oligomers containing adenine^29^. Although this competitor (AD1) functioned as an effective antidote by reversing inhibition, the kinetics of the antidote were deemed too slow at low concentrations (1–10 μM; Extended Data Fig. 7). Mindful of the observed cooperativity between target interaction and hybridization, we introduced a toehold sequence^30^ on the supramolecular connector (A8–E1) to achieve a larger equilibrium shift in the hybridization with AD2, a 12-mer PNA (Fig. 4a,b). Following the kinetic progress of the reaction in real time with a fluorogenic substrate, we observed the ability to switch from complete inhibition (15 nM binary inhibitor) to ~40% of uninhibited thrombin activity within 30 min using 10 μM antidote (Fig. 4c). Using lower concentrations of antidote resulted in more progressive restoration of thrombin activity. Using just 1 equiv. of antidote was sufficient to restore ~20% of thrombin catalytic activity within 90 min. These observations were also validated in the fibrinogen clotting and CAT assays described earlier, with clotting restored using 1 equiv. and 5 equiv. of antidote relative to the supramolecular inhibitor, respectively (Fig. 4d,e; A8–E1 + AD2). Based on these promising in vitro data, we assessed the ability of our designed antidote to reverse anticoagulation in the in vivo thrombosis model. In this experiment, we first treated animals with our supramolecular construct (5 mg per kg (body weight), the concentration that provided effective anticoagulation in the needle injury thrombosis model), followed by administration of 5 molar equiv. of the 12-mer PNA antidote (9.4 mg per kg (body weight)). Following addition of the antidote, anticoagulation was effectively reversed, as determined by the amount of fibrin deposition and thrombus volume compared to control injuries lacking treatment with the antidote (Fig. 4f,g). These data support the potential of supramolecular inhibitors as bona fide therapeutic leads and lay the foundation for targeting a range of therapeutic targets with this approach in the future.

## Discussion

We have designed highly potent direct bivalent thrombin inhibitors that display an 800-fold gain in activity relative to individual fragments by applying the constitutional dynamic properties of supramolecular binary fragments. The supramolecular pairing was achieved with PNA, allowing simple tuning of the dissociation kinetics of the supramolecular complex. An important point of difference between these molecules and classical inhibitors is that the dynamic equilibrium can be modulated by external factors, yielding a simple strategy for reversing inhibition. This feature is highly pertinent for direct thrombin inhibition due to the risk of adverse bleeding side effects in anticoagulation therapy. These known side effects have stimulated the development of several antidotes to clinically approved anticoagulant drugs^31^ that center on the use of expensive monoclonal antibodies and cocktails of competitive coagulation proteins. Our designed supramolecular anticoagulants showed potent thrombin inhibition and anticoagulation activities in vitro that could be rapidly reversed using small PNA-based antidotes. This potent anticoagulant activity with on-demand reversibility was also demonstrated in an in vivo thrombosis model, providing a starting point for future use of this therapeutic modality for anticoagulant drug candidates. PNAs are known to be metabolically stable and, unless purposefully modified, cell impermeant^32^. These features make PNA a good choice to focus the activity of supramolecular drugs on extracellular targets, limiting off-target effects^33^ and any intrinsic pharmacological activity for the antidote. Future improvements could make use of γ*-*modified PNA^34^ with d-stereochemistry to preclude interaction with endogenous extracellular oligonucleotides^35,36^.

The strategy adopted here offers a general mechanism to turn therapeutic activity on or off rapidly and is therefore not limited to applications in thrombosis. For example, the supramolecular concept could be beneficial in immunotherapy when an antidote to a chimeric antigen receptor T cell response is desired or to reverse the action of immunomodulators in case of severe infection. The fact that assembly can be encoded by different sequences of low-cost PNA should make it possible to multiplex programmable supramolecular drug candidates. This strategy requires the identification of two fragments that bind synergistically to a protein of interest. DNA-encoded libraries making use of dual display are poised to deliver such fragments for new targets lacking prior information^37,38^. The same approach can also be considered with Fab antibody fragments^39^.

## Methods

### General methods

Unless otherwise specified, all reagents and solvents for all organic synthesis procedures were purchased from commercial sources and were used without further purification. High-performance liquid chromatography (HPLC) purification was performed with an Agilent Technologies 1260 Infinity HPLC using a ZORBAX 300SB-C18 column (9.4 × 250 mm). LC–mass spectrometry (LC–MS) spectra were recorded on a DIONEX Ultimate 3000 UHPLC with a Thermo LCQ Fleet Mass Spectrometer System using a PINNACLE DB C18 column (1.9 µm, 50 × 2.1 mm) operated in positive mode. All the LC–MS spectra were measured by electrospray ionization. Matrix-assisted laser desorption/ionization–time of flight (MALDI–TOF) mass spectra were measured using a Bruker Daltonics Autoflex spectrometer operated in positive mode. High-resolution mass spectra were obtained on a Xevo G2 TOF spectrometer (ionization mode, electrospray ionization positive polarity; mobile phase, methanol at 100 µl min^–1^). Automated solid-phase synthesis was performed on an Intavis AG Multipep RS instrument.

### Synthesis of PNA–peptide conjugates

Resin (5.0 mg) was swollen in dichloromethane (DCM) for 10 min and washed twice with dimethylformamide (DMF). Iterative cycles of amide coupling (procedure 1), capping of the resin (procedure 4) and deprotection of the protecting group (procedure 2 or procedure 3) were performed to synthesize the PNA probes. The compounds were deprotected and cleaved from the resin using procedure 5 and finally purified by HPLC. Characterization of the PNA–peptide conjugates was performed using MALDI (Bruker Daltonics Autoflex spectrometer with Flex control 3.4 software and analysis with FlexAnalysis 3.4) and/or LC–MS (DIONEX Ultimate 3000 UHPLC with a Thermo LCQ Fleet Mass Spectrometer System using a PINNACLE DB C18 column (1.9 µm, 50 × 2.1 mm) with Thermo Xcalibur 2.2.SP1.48 software and analysis with Thermo Xcalibur Qual Browser 2.2.Sp1.48). For MALDI analysis, 1.0 µl of the sample (in either water or water/acetonitrile (1:1)) was mixed with 1.0 µl of 2,5-dihydroxybenzoic acid (DHB) matrix solution (30 mg of DHB in 1.0 ml of 70:30:0.01 water/acetonitrile/trifluoroacetic acid (TFA)), and the mixture was spotted on a MALDI plate. The measurements were acquired in positive linear mode. For LC–MS analysis, 20 µl of sample in water or water/acetonitrile (1:1) was injected on the LC and further analyzed by MS in positive mode. Compounds containing the benzene disulfonic acid motif could only be analyzed by LC–MS due to fragmentation when analyzed by MALDI.

#### 2-Chlorotrityl chloride resin loading

2-Chlorotrityl chloride resin (1.46 mmol g^–1^ loading) was swollen in dry DCM for 30 min, followed by washing with DCM + 1% *N*,*N*-diisopropylethylamine (DIPEA; 3 ml, one time) and DCM (3 ml, ten times). A solution of Fmoc-Xaa-OH (0.7 mmol g^–1^ resin) and DIPEA (4 equiv. relative to resin functionalization) in DCM (final concentration of 0.125 M amino acid) was added to the resin, which was shaken at room temperature for 16 h. The resin was then washed with DCM (3 ml, five times), DMF (3 ml, five times) and DCM (3 ml, five times). The resin was then capped via treatment with 17:2:1 (vol/vol/vol) DCM/methanol/DIPEA (5 ml) for 40 min at room temperature. The resin was then washed again with DCM (3 ml, five times), DMF (3 ml, five times) and DCM (3 ml, five times) before further use.

#### Rink amide resin loading

Nova PEG Rink amide resin (0.44 mmol g^–1^; Novabiochem) was swollen in DCM for 10 min and washed twice with DMF. Standard amide coupling (procedure 1) was performed, followed by capping of the resin (procedure 4). The resin was then washed again with DCM (3 ml, five times), DMF (3 ml, five times) and DCM (3 ml, five times) before further use.

#### Amide coupling (procedure 1)

The corresponding Fmoc-protected PNA monomer^40^ or amino acid (4 equiv., 0.2 M in *N*-methylpyrrolidone (NMP)) was incubated for 5 min with HATU (3.5 equiv., 0.5 M in NMP) and base solution (1.2 M (4 equiv.) DIPEA and 1.8 M (6.0 equiv.) 2,6-lutidine in NMP). The mixture was then added to the corresponding resin. After 20 min, the mixture was filtered, the resin was washed with DMF, and a new premixed reaction solution was added to the resin and allowed to react for another 20 min. The resin was then washed sequentially with DMF, DCM and DMF two times each.

#### Fmoc deprotection (procedure 2)

A solution of 20% (vol/vol) piperidine in DMF was added to the resin and allowed to react for 5 min. The mixture was then filtered, the resin was washed with DMF, and the sequence was repeated for another 5 min. The resin was then washed sequentially with DMF, DCM and DMF two times each.

#### 4-Methyltrityl deprotection (procedure 3)

A solution (made from 244 mg of hydroxybenzotriazole in 10 ml of hexafluoroisopropanol and 10 ml of 1,2-dichloroethane) was added to the prewashed resin to reach a volume of 10 ml g^–1^ of resin and allowed to react for 5 min. The solution was flushed, the resin was washed with DCM, and the sequence was repeated for another 5 min. Finally, the resin was washed sequentially with DCM and DMF two times each.

#### Capping (procedure 4)

The resin was treated with a capping mixture (0.92 ml of acetic anhydride and 1.3 ml of 2,6-lutidine in 18 ml of DMF; 10 ml of solution per g of resin) for 5 min. After flushing the solution, the resin was washed sequentially with DMF, DCM and DMF two times each.

#### Cleavage from the resin and final deprotection (procedure 5)

Resin (5.0 mg, 1.0 μmol) was treated with 125 μl of a mixture of TFA and scavengers (440 µl of TFA + 25 mg of phenol + 25 µl of water + 10 µl of triisopropylsilane) for 2 h. The resin was filtered and washed with TFA (50 μl), and the collected fractions of cleavage product were precipitated in cold ether (1.5 ml). After centrifugation, the pellet was vortexed again with cold diethyl ether (1.5 ml) and centrifuged (18,000*g*). The pellet was dissolved in water/acetonitrile (3:1; 1.5 ml) and lyophilized to obtain a white powder.

#### Microcleavage for quality control (procedure 6)

The minimum number of beads was picked with a pipette plastic tip and transferred to 50 µl of TFA. The solution was left for 1 h and transferred to 1.0 ml of ether. The ether solution was maintained at −20 °C for 5 min and centrifuged for 5 min at 18,000*g*. The ether supernatant was removed, and the pellet was dissolved in 20 µl of 1:1 acetonitrile/water, which was then used for analysis by MALDI–TOF and/or LC–MS.

#### On-resin copper(I)-catalyzed azide-alkyne cycloaddition (CuAAC; procedure 7)

A solution of CuSO_4_ (15 µl, 64 mg ml^–1^ in water) was added to tris(benzyltriazolylmethyl)amine (2 mg) in 20 µl of DMF, followed by the addition of 50 µl of sodium ascorbate (396 mg ml^–1^ in water). Azide-containing peptide (2 equiv. in 60 µl of DMF) was added to the mixture, which was mixed before the addition of 5 mg of alkyne-derivatized Rink amide resin (0.0022 mmol). After 16 h of shaking, the mixture was filtered, and the resin was washed six times with 250 µl of sodium diethyl dithiocarbamate (0.02 M) in DMF, six times in 250 µl of DMF, six times in methanol and six times in DCM.

#### Coupling of Fmoc-l-F_2_Smp(nP)-OH (procedure 8)

Fmoc-l-F_2_Smp(nP)-OH was prepared as previously described^22^. A mixture of Fmoc-l-F_2_Smp(nP)-OH (0.003 mmol, 1.5 equiv.), hydroxybenzotriazole (0.003 mmol, 1.5 equiv.) and *N*,*N*′-diisopropylcarbodiimide (0.003 mmol, 1.5 equiv.) was added to the corresponding resin and shaken overnight. The mixture was filtered, and the resin was washed sequentially with DMF, DCM and DMF two times each.

#### Coupling of Arg(Pbf)-benzothiazole (procedure 9)

Arg(Pbf)-benzothiazole (0.0044 mmol, 2 equiv.) and HATU (0.0034 mmol, 1.5 equiv.) in NMP (100 μl) were added to 5 mg of resin (0.0022 mmol), followed by the addition of DIPEA (0.012 mmol, 6 equiv.). The reaction was shaken for 2 h, the mixture was filtered, and the resin was washed sequentially with DMF, DCM and DMF two times each.

#### Neopentyl deprotection and characterization of PNA–peptide conjugates (procedure 10)

The precipitate collected after cleavage and ether precipitation was lyophilized. The remaining solid was dissolved in a solution of 1 M ammonium acetate and 6 M guanidinium chloride and shaken at 37 °C for 2 h. The solution was then diluted with water/acetonitrile (50:50) and purified by HPLC.

### Thrombin inhibition assay

Inhibition of the activity of human α-thrombin (Haematologic Technologies, HCT0020) was followed spectrophotometrically using Phe-Pro-Arg-Coumarin (synthesis described in Supplementary Information) as the chromogenic substrate.

Inhibition assays were performed using 0.2 nM enzyme, 20 μM substrate and increasing concentrations of inhibitor. The concentration of each inhibitor variant was determined using the absorbance of the PNA at 260 nm, as measured by NanoDrop. All reactions were performed at 37 °C in 50 mM Tris-HCl (pH 8.0), 50 mM NaCl and 1 mg ml^–1^ bovine serum albumin in black 96-well microtiter plates (Thermo Fisher Scientific, 267342). Reaction progress was monitored by excitation at 339 nm and emission at 439 nm using a SpectraMax or Tecan Spark Plate Reader. Dose–response curves were used to determine the half-maximal inhibitory concentrations (IC_50_) using Prism 8.0 (GraphPad Software). For each inhibitor, the reactions were performed in triplicate, together with control reactions in the absence of enzyme. The initial velocity was calculated from the slope of the first 10 min of the assay. The curves were normalized to the well without inhibitor, where the initial velocity was set to 100% activity.

For the antidote assay, the plate was removed from the plate reader at the desired time of addition (usually 30 min). One microliter of antidote (100×) was added, and reading was resumed.

### Fibrinogen assay

Human α-thrombin (Haematologic Technologies, HCT0020; final concentration of 2.5 nM) was incubated with compound (final concentration of 15 nM) at 37 °C for 30 min. Fibrinogen (final concentration of 1 mg ml^–1^) was added, and absorbance at 288 nm was measured using a SpectraMax Plate Reader. All reactions were performed at 37 °C in 50 mM Tris-HCl (pH 8.0), 50 mM NaCl and 1 mg ml^–1^ bovine serum albumin in clear 96-well microtiter plates (Greiner Bio-One, 650201).

For the antidote assay, the plate was removed from the plate reader at the desired time of addition (usually 30 min). One microliter of antidote (100×) was added, and reading was resumed.

### Selectivity assays

Inhibitory activity of A1–E1 was tested against α-human thrombin, FXIa and FXa (Haematologic Technologies) and α-FXIIa and PK (Enzyme Research Laboratories). Chromogenic assays were followed spectrophotometrically using the following specific substrates: 100 μM Tos-Gly-Pro-Arg-PNA (Chromozym TH, Roche) for thrombin, 500 µM Pyr-Pro-Arg-PNA (L-2145, Bachem) for FXIa, 500 µM Moc-d-norleucine-Gly-Arg-PNA (L-1565, Bachem) for FXa and 200 µM or 400 µM d-Pro-Phe-Arg-PNA (Cayman Chemical) for α-FXIIa or PK, respectively. The assay buffers included 50 mM Tris-HCl (pH 8.0) and 50 mM NaCl for thrombin (0.2 nM); PBS (pH 7.4) for FXIa (0.5 nM); 25 mM Tris-HCl (pH 7.5), 100 mM NaCl and 5 mM CaCl_2_ for FXa (0.5 nM); 20 mM HEPES (pH 7.6), 150 mM NaCl, 0.1% (wt/vol) PEG 8000 and 0.01% (vol/vol) Triton X-100 for α-FXIIa (4 nM); and 50 mM Tris-HCl (pH 8.0) and 150 mM NaCl for PK (0.25 nM). Bovine serum albumin (Sigma) was added to all buffers at 1 g l^–1^. All reactions were initiated by the addition of the protease and were performed at 37 °C in 96-well, flat-bottom microtiter plates. Reaction progress was monitored at 405 nm for 30 min (60 min for FXa and α-FXIIa) on a multimode microplate reader (Synergy2, BioTek) with measurements taken every 5 min. All measurements were performed in duplicate. IC_50_ values were determined from the log dose–response curves with Prism 9 (GraphPad Software).

### SPR experiments

SPR experiments were performed on a Biacore T200 instrument (GE Healthcare) at 25 °C in PBS-P+ buffer (10× stock; Cytiva Life Sciences, 28995084). Biotin-PNA (8-mer) was immobilized on a Streptavidin Series S sensor chip (Cytiva Life Sciences, 29104992). Before immobilization, the two channels were conditioned with 1 M NaCl in 50 mM NaOH. After stabilization, the compound (solution in PBS-P+) was flowed over one of the flow cells of the sensor chip at a concentration of 50 nM at a flow rate of 10 μl min^−1^ with a response unit target of 500. Biotin-PNA (8-mer) reached a response unit value of 513.7. The system (not including the flow cells) was washed with 50% isopropanol in 1 M NaCl and 50 mM NaOH after each ligand injection. Kinetic measurements consisted of injections (association, 400 s; dissociation, 450 s; flow rate, 30 μl min^−1^) of decreasing concentrations of PNA (4-, 6- and 8-mer; twofold cascade dilutions from the starting concentration). The chip was regenerated between cycles by one injection of regeneration solution (50 mM NaOH) for 10 s at a flow rate of 20 μl min^−1^, followed by a 10-s stabilization period. Binding was measured as resonance units over time after blank subtraction, and the data were interpreted using Biacore T200 software (version 3.2). All measurements were performed in duplicate. The *K*_D_ values were calculated based on steady-state affinity (1:1 binding).

### aPTT in vitro

aPTT measurements were performed on a BFT II benchtop analyzer as per the manufacturer’s instructions. Dade Actin FSL Activated PTT Reagent (23-044-647) and calcium chloride solution (10446232 ORHO37) were both sourced from Siemens Healthcare Diagnostics Products, and lyophilized pooled human reference plasma (Pooled Norm., 00539) was purchased from Diagnostica Stago. Pooled human plasma was reconstituted as per the manufacturer’s instructions (Milli-Q water, 30 min, room temperature). Pooled mouse plasma was prepared by collection of whole blood from three to four C57BL/6 mice (Australian BioResources) into sodium citrate (3.8%), with plasma isolated by centrifugation at 5,000*g* for 15 min and stored on ice until required.

Human or mouse plasma was incubated with inhibitors at the indicated concentrations and prewarmed to 37 °C. Fifty microliters of each plasma/inhibitor mixture was incubated with Actin FSL (50 ml) in a stirred reaction vessel for 3 min before addition of 50 ml of calcium chloride solution to initiate coagulation. The time taken for fibrin clot formation was recorded in a semiautomated fashion using a BFT II Analyzer, which uses a turbodensitometric detection technique.

### Ex vivo aPTT

All procedures involving the use of animals were performed as approved by the University of Sydney Animal Ethics Committee (protocol 2021/1912). C57BL/6 mice (25–30 g) were anesthetized using a mixture of ketamine (125 mg per kg (body weight)) and xylazine (12.5 mg per kg (body weight); intraperitoneal delivery) and administered A1–E1 as a single bolus delivered intravenously via the femoral vein at either 2.5 or 5.0 mg per kg (body weight). Blood was drawn from the inferior vena cava at the indicated times into citrate anticoagulant (3.8%), plasma was isolated as described above for in vitro aPTT studies, and aPTT was assessed via changes in plasma opacity at 405 nm using a CLARIOstar plate reader fitted with dual injectors heated to 37 °C using a modified version of the aPTT protocol described above. Briefly, injectors were primed for Dade Actin FSL Activated PTT Reagent (line A) and calcium chloride solution (line B), and mouse plasma was aliquoted in duplicate (25 μl) into wells of a Nunc 368-well polystyrene plate (Z723010, Sigma-Aldrich). Following injection of 25 ml of Dade Actin FSL, the plate was mixed using the orbital shaking function for 2 s (500 rpm) and incubated for 182 s at 37 °C. At this time (designated *t* = 0 s), 25 ml of calcium chloride solution was injected, the plate was mixed as described above, and absorbance measurements were taken at 405 nm for 360 intervals (22 flashes per well, interval time of 0.5 s). Clotting time was denoted by the timing of initial inflection point, denoting transition of plasma from transparent to opaque.

### CAT

Normal lyophilized human pooled plasma (Pool Norm., 00539, Diagnostica Stago) was reconstituted and incubated for 30 min at 37 °C. Vehicle and various inhibitors at different concentrations were then incubated in plasma for 30 min. Thrombin assays were performed using a Hemker Calibrated Automated Thrombinoscope (Diagnostica Stago) and a Fluoroskan Ascent plate reader (Thermo Fisher Scientific). All experiments were conducted in triplicate in 96-well microplates for fluorescence-based assays (M33089, Thermo Fisher Scientific) and calibrated using untreated plasma and a thrombin calibrator (86192, Diagnostica Stago). Thrombinoscope experiments were conducted following patented commercial protocols. In brief, each sample well was filled with 20 μl of PPP reagent containing a mixture of phospholipids and tissue factor (86193, Diagnostica Stago). Eighty microliters of plasma (untreated/ treated) was then added to each of these wells and mixed using reverse pipetting, and the well plate was incubated in the plate reader at 37 °C for 10 min. Meanwhile, a FluCa kit (86197, Diagnostica Stago) containing Fluo-Buffer and Fluo-Substrate was warmed to 37 °C. Following incubation, the thrombinoscope dispenser was flushed, emptied and filled with a FluCa mixture consisting of the Fluo-Buffer and Fluo-Substrate. Twenty microliters of the FluCa mixture was dispensed into each well containing plasma samples, initiating the coagulation reaction. Thrombin activity (nM) was measured over 1 h, with thrombogram parameters including lag time (min), velocity index (nM min^–1^), time to peak (min), peak height (nM), endogenous thrombin potential (nM × min) and time to tail (min).

### Needle injury thrombosis model

C57BL/6J mice were purchased from Australian BioResources and housed at the Laboratory Animal Services facility (University of Sydney). All animals were maintained on a 12-h light/12-h dark cycle with access to food and water ad libitum. For intravital mouse studies, male mice aged between 5 and 8 weeks old (15–20 g) were used. All studies were approved by the University of Sydney Animal Ethics Committee (protocol 2021/1912) in accordance with the requirements of the Australian Code of Practice for the Care and Use of Animals for Scientific Purposes^41^.

A clinical preparation of argatroban (Argatra/Exembol) was purchased from Mitsubishi Tanabe Pharma (Germany) and prepared in sterile saline with 25% (vol/vol) propylene glycol. Synthesized PNA inhibitors and PNA inhibitors + antidote solutions were prepared in sterile saline at a concentration of 2 mg ml^–1^. C57BL/6J male mice (15–25 g) were anesthetized with ketamine (150 mg per kg (body weight)) and xylazine (15 mg per kg (body weight)), supplemented with oxygen and subjected to intravital needle injury, as previously described^42^. Systemic injection of DyLight 649-conjugated anti-GP1bβ (X649, Emfret; 100 µg kg^–1^) and Alexa 546-conjugated anti-fibrin (0.31 mg per kg (body weight)) was performed before vessel injury to monitor thrombus formation and fibrin generation, respectively. Argatroban (80 µg kg^–1^ bolus, 40 µg kg^–1^ min^–1^, 60-min infusion) was delivered via a jugular catheter using a Harvard apparatus pump (704504, Pump 11 Elite I/W Single Syringe Pump). Injections of PNA inhibitors or PNA inhibitors + antidote (5 mg per kg (body weight) bolus every 30 min) were delivered intravenously. Two to four successive injuries were created in multiple vessels in each mouse from each treatment group. Following each injury, platelet thrombus formation and fibrin generation were monitored over a 15-min period using a confocal intravital microscopy platform (Nikon A1R-Si with an Apo LWD, ×40/1.15-NA water immersion objective; sequential excitation: 488-, 561- and 638-nm lasers; emission: 525/50-, 595/50- and 700/75-nm filters) and NIS Elements Advanced Research acquisition software. The microscope stage and objective were maintained at 37 °C throughout the experiment via a Peltier heater (OkoLab). Surface renders of confocal stacks representing thrombi from separate groups were generated using Imaris (version 9.8, Bitplane).

#### Quantitative analysis of thrombus volume over time

NIS Elements software (version. 5.02; Nikon) was used to apply a threshold to DyLight 649-conjugated anti-GP1bβ signal for each *xyz* stack in a time series and was used to calculate the volume for each time point.

#### Quantitation of change in fibrin amount over time

The signal obtained from DyLight 649-conjugated anti-GP1bβ for each *xyz* stack in a time series was thresholded to create a mask. The total signal (arbitrary units) from Alexa Fluor 546-conjugated anti-fibrin within this mask (that is, the fibrin signal within the thrombus) for each time point was then quantified using NIS Elements software (Nikon).

### Statistical analysis

Statistical significance between multiple treatment groups was analyzed using a one-way ANOVA with Tukey’s post hoc testing with a single pooled variance (Prism software version 10.2; GraphPad Software for Science). Data are presented as mean ± s.e.m., where ‘*n*’ equals the number of independent experiments performed.

### Reporting summary

Further information on research design is available in the Nature Portfolio Reporting Summary linked to this article.

## Online content

Any methods, additional references, Nature Portfolio reporting summaries, source data, extended data, supplementary information, acknowledgements, peer review information; details of author contributions and competing interests; and statements of data and code availability are available at 10.1038/s41587-024-02209-z.

## Supplementary information

Supplementary Information Supplementary Figs. 1–6, Tables 1 and 2, synthesis and characterization of Arg(Pbf)-benzothiazole, synthesis and characterization of Phe-Pro-Arg-Coumarin, characterization of PNA–peptide compounds, thrombin inhibition assay methods, and needle injury thrombosis model maximum intensity projections.

Reporting Summary

Supplementary Data Raw NMR data.

Supplementary Data Raw images of maximum intensity projections.

Source Data for Supplementary Figs. 3–6 Source data for supplementary figures.

## Source data

Source Data Fig. 2 IC_50_ curves.

Source Data Fig. 3 In vivo time course and bar chart plots.

Source Data Fig. 4 Thrombin inhibition (fluorogenic and fibrinogen assay) kinetic experiments, CAT and in vivo time course and bar chart plots.

Source Data Extended Data Fig. 3 IC_50_ and SPR curves.

Source Data Extended Data Fig. 4 IC_50_ curves and thrombin inhibition (fluorogenic assay) kinetic experiments.

Source Data Extended Data Fig. 5 Thrombin inhibition (fibrinogen assay) kinetic experiments, ex vivo human and mouse aPTT.

Source Data Extended Data Fig. 6 CAT.

Source Data Extended Data Fig. 7 Thrombin inhibition (fluorogenic assay) kinetic experiments and CAT.

## Data Availability

The data supporting the findings of this study are available within this paper and its Supplementary Information. All raw data have been deposited on Zenodo (10.5281/zenodo.10473739)^43^. Previously published PDB ID numbers that are mentioned and shown in the main text can be found online with the following codes: tick-derived madanin-1 (PDB 5L6N) and TTI from the tsetse fly (PDB 6TKG). Source data are provided with this paper.
