# Supplementary material for: Development of supramolecular anticoagulants with on-demand reversibility
Source: Nat Biotechnol. 2024 Apr 30;43(2):186–93. doi: 10.1038/s41587-024-02209-z (PMC11825364; doi:10.1038/s41587-024-02209-z)
Supplement: Supplementary file 1 — Supplementary Figs. 1–6, Tables 1 and 2, synthesis and characterization of Arg(Pbf)-benzothiazole, synthesis and characterization of Phe-Pro-Arg-Coumarin, characterization of PNA–peptide compounds, thrombin inhibition assay methods, and needle injury thrombosis model maximum intensity projections. [file 41587_2024_2209_MOESM1_ESM.pdf]

nature biotechnology

Article

https://doi.org/10.1038/s41587-024-02209-z

Development of supramolecular anticoagulants with on-demand reversibility

In the format provided by the authors and unedited

Table of Contents

1. **Acronyms** 2
2. **Synthesis of Arg(Pbf)-Benzothiazole** 3
3. **Synthesis of phe-Pro-Arg-Coumarin** 7
4. **Characterisation of PNA-peptide compounds** 12
5. **Thrombin Inhibition Assay** 43
6. **Needle Injury Thrombosis Model – Max Intensity Projections** 45
7. **References** 46

1. Acronyms

CuAAC	Copper(I)-catalyzed azide-alkyne cycloaddition
DAP	Diaminopropionic acid
DCE	1,2-dichloroethane
DCM	Dichloromethane
DHB	2,5-Dihydroxybenzoic acid
DIPEA	N,N-Diisopropylethylamine
DMF	Dimethylformamide
DMSO	Dimethyl sulfoxide
EDC	1-Ethyl-3-(3-dimethylaminopropyl)carbodiimide
ESI	Electrospray ionization
Fmoc	Fluorenylmethoxycarbonyl
HATU	Hexafluorophosphate Azabenzotriazole Tetramethyl Uronium
HIFP	Hexafluoroisopropanol
HOBt	Hydroxybenzotriazole
HPLC	High performance liquid chromatography
HRMS	High-resolution mass spectra
LCMS	Liquid chromatography-mass spectrometry
MALDI	Matrix-assisted laser desorption/ionization
MS	Mass spectrometry
NaAsc	Sodium ascorbate
NMP	N-Methyl-2-pyrrolidone
PEG	Polyethylene glycol
PNA	Peptide Nucleic Acid
TBTA	Tris((1-benzyl-4-triazolyl)methyl)amine
TFA	Trifluoroacetic acid
UHPLC	Ultra High performance liquid chromatography

The general methods and synthetic procedures are described in the Online Methods section.

2. Synthesis of Arg(Pbf)-Benzothiazole

Tert-butyl (S,Z)-(1-(methoxy(methyl)amino)-1-oxo-5-(2-((2,2,4,6,7-pentamethyl-2,3-dihydrobenzofuran-5-yl)sulfonyl)guanidino)pentan-2-yl)carbamate

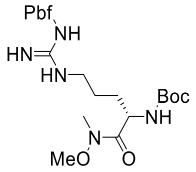

Chemical Formula: $C_{26}H_{43}N_5O_7S$, **Exact Mass**: 569.29, **Molecular Weight**: 569.72.

Boc-Arg(Pbf)-OH was prepared as previously described by Jakobsche *et al.*[1] Boc-Arg(Pbf)-OH (1 g, 1.90 mmol) was dissolved in dry THF under N_2. HATU (2.28 mmol, 1.2 equiv.) and DIPEA (9.5 mmol, 5 equiv.) were added, followed by MeNHOMe (2.28 mmol, 1.2 equiv.). The mixture was stirred at room temperature for 2.5 hours. The mixture was concentrated under *vacuo*, water was added and the product was extracted with EtOAc (3x), the organic layers were washed with H_2O (2x), Brine (1x), dried over Na_2SO_4, and concentrated. The crude material was purified by flash chromatography (20% pentane in ethyl acetate) to yield the Weinreb amide (953 mg, 1.67 mmol, 88%).

LCMS (ESI); RT= 2.63, $[M+1H]^{1+}$: 570.05.

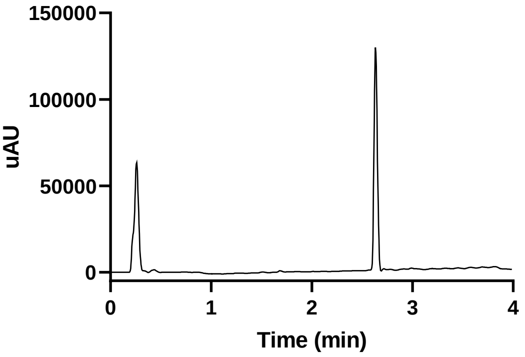

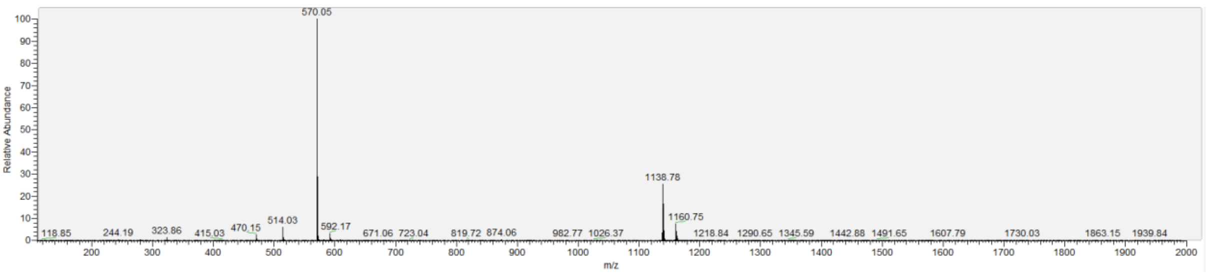

NMR; ^{1}H NMR (400 MHz, $CDCl_3$) δ 6.51 (s, 2H), 5.50 (d, *J* = 8.8 Hz, 1H), 4.63 (s, 1H), 3.74 (s, 3H), 3.42 (d, *J* = 7.6 Hz, 1H), 3.20 (s, 3H), 2.96 (s, 2H), 2.80 (s, 7H), 2.55 (s, 3H), 2.50 (s, 3H), 2.09 (s, 3H), 1.78 – 1.52 (m, 4H), 1.47 (s, 6H), 1.42 (s, 9H).

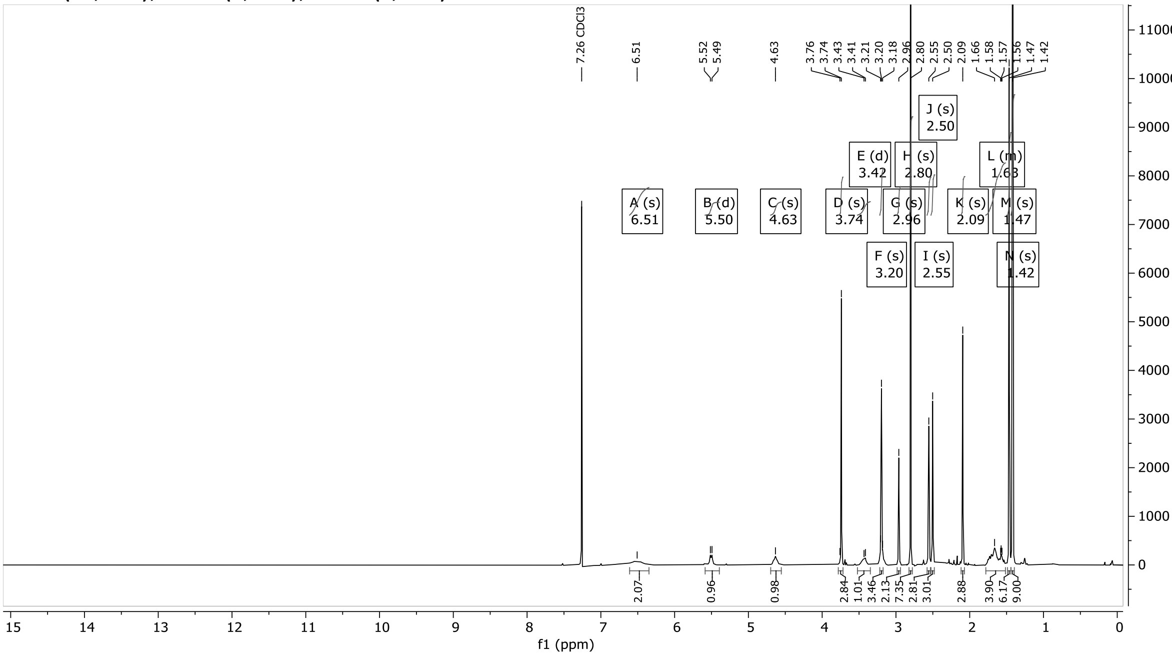

tert-butyl (S)-(1-(benzo[d]thiazol-2-yl)-1-oxo-5-(3-((2,2,4,6,7-pentamethyl-2,3-dihydrobenzofuran-5-yl)sulfonyl)guanidino)pentan-2-yl)carbamate

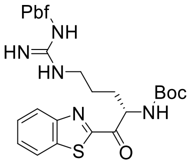

Chemical Formula: $C_{31}H_{41}N_5O_6S_2$, **Exact Mass**: 643.25, **Molecular Weight**: 643.82

Benzothiazole (6.3 mmol, 18 equiv.) was added to dry THF (21 mL) under N_2 and cooled to -78 °C. n-BuLi (6.125 mmol, 17.5 equiv.) was added dropwise over 10 minutes and stirred for 30 minutes at -78 °C. A solution of Weinreb amide (0.35 mmol) in THF (11 mL) was added dropwise at -78 °C and stirred for 2 hours. The reaction was quenched with 10 mL of saturated NH_4Cl and warmed to room temperature. The mixture was extracted with EtOAc (3x), the organic layers were washed with H_2O (2x), Brine (1x) and dried over Na_2SO_4 and concentrated. The crude product was purified by flash chromatography (10 to 100% EtOAc in Pentane) to yield Boc-Arg(Pbf)-Benzothiazole as brown solid (170 mg, 0.26 mmol, 75%).

LCMS (ESI); RT= 3.13, $[M+1H]^{1+}$: 644.06

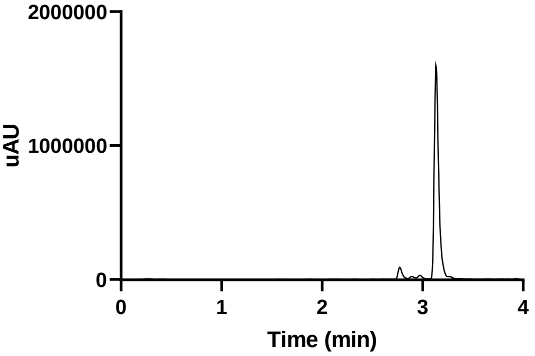

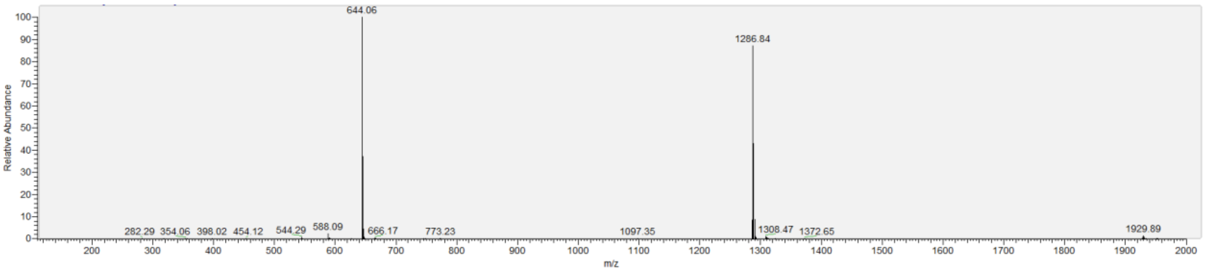

NMR; ^{1}H NMR (400 MHz, $CDCl_3$) δ 8.25 – 8.15 (m, 1H), 8.05 – 7.89 (m, 1H), 7.66 – 7.49 (m, 2H), 6.38 (s, 1H), 6.26 (s, 2H), 5.74 – 5.51 (m, 2H), 3.32 (s, 2H), 2.95 (s, 2H), 2.56 (s, 3H), 2.51 (s, 3H), 2.08 (s, 3H), 1.70 – 1.66 (m, 4H), 1.45 (d, *J* = 9.5 Hz, 15H).

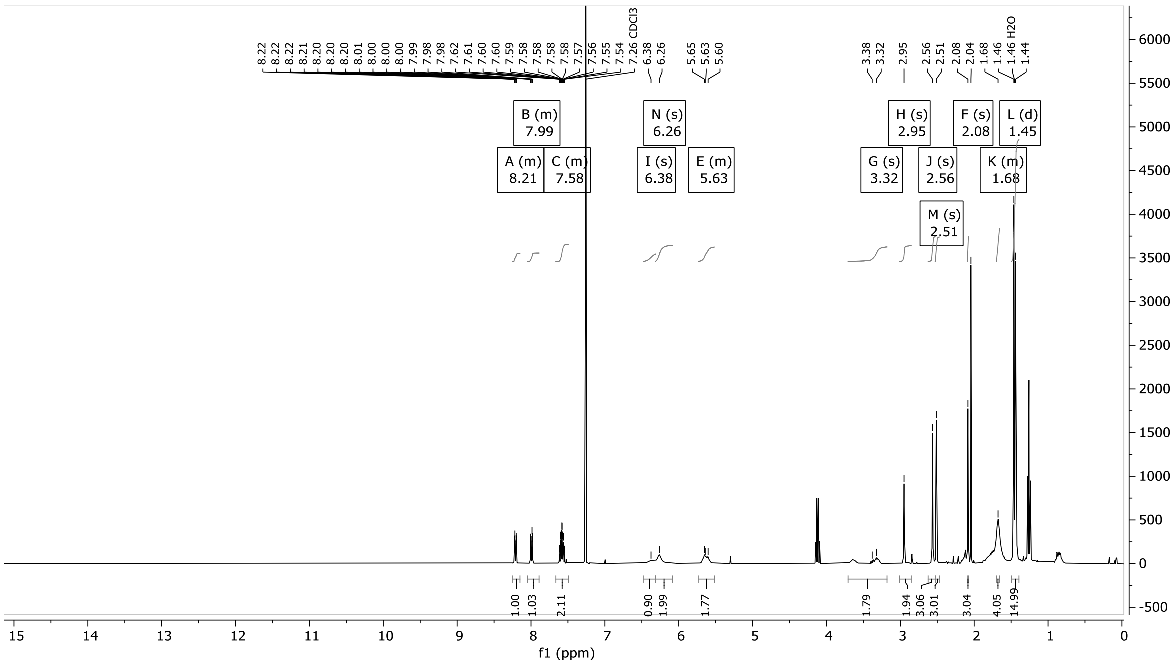

(S)-N-(N-(4-amino-5-(benzo[d]thiazol-2-yl)-5-oxopentyl)carbamimidoyl)-2,2,4,6,7-pentamethyl-2,3-dihydrobenzofuran-5-sulfonamide

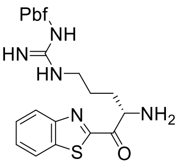

Chemical Formula: $C_{26}H_{33}N_5O_4S_2$, **Exact Mass**: 543.20, **Molecular Weight**: 543.70.

Boc-Arg(Pbf)-Benzothiazole (74 mg, 0.115 mmol) was dissolved in dioxane and cooled to 0 °C. 4M HCl in dioxane was added and the mixture was stirred for 2 hours at room temperature. The mixture was concentrated under *vacuo* to yield Arg(Pbf)-Benzothiazole as a brown solid (63 mg, quant.). The compound was used without further purification.

LCMS (ESI); RT= 2.22, $[M+1H]^{1+}$: 544.18.

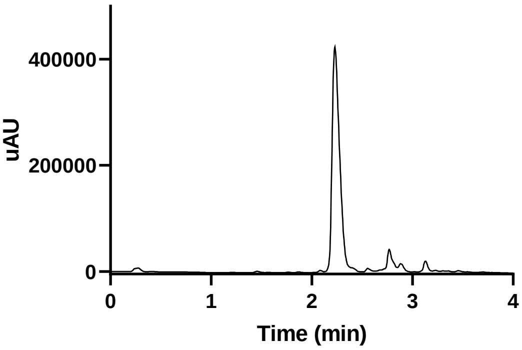

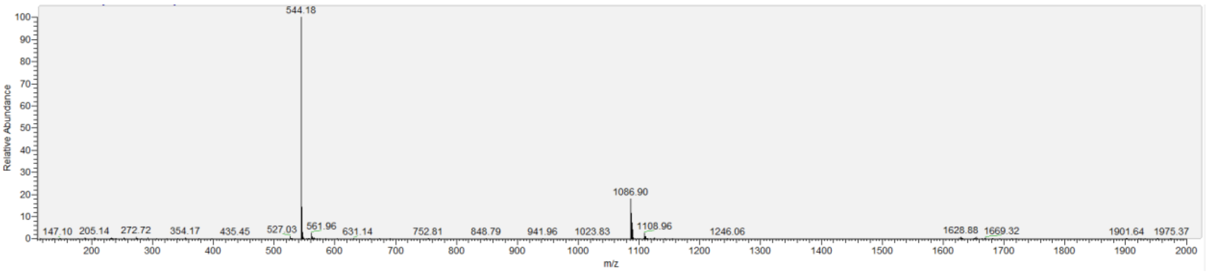

NMR; ^{1}H NMR (400 MHz, $CDCl_3$) δ 8.89 (s, 2H), 8.39 (s, 1H), 8.22 – 8.13 (m, 1H), 7.83 (s, 1H), 7.49 (s, 2H), 5.52 (s, 1H), 3.74 (d, *J* = 7.5 Hz, 1H), 3.62 – 3.35 (m, 2H), 2.90 (s, 2H), 2.54 (d, *J* = 6.3 Hz, 2H), 2.41 (d, *J* = 19.4 Hz, 6H), 2.19 (s, 2H), 1.43 (s, 6H).

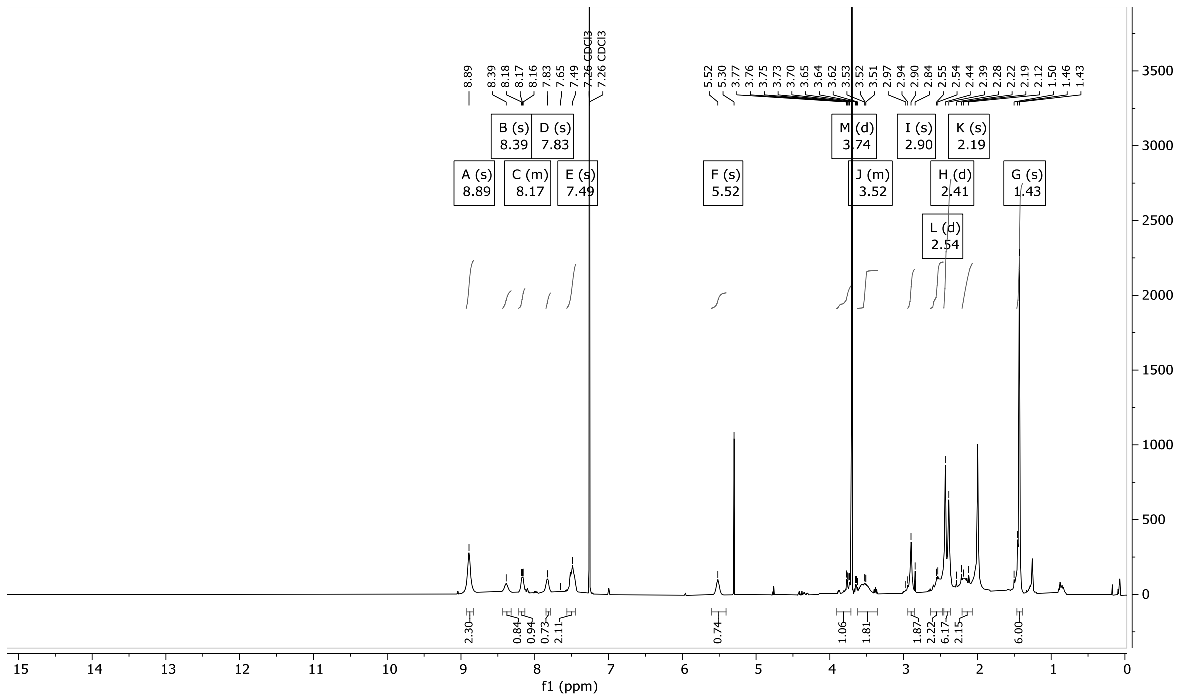

3. Synthesis of phe-Pro-Arg-Coumarin

(9H-fluoren-9-yl)methyl (S)-(1-((4-methyl-2-oxo-2H-chromen-7-yl)amino)-1-oxo-5-(3-((2,2,4,6,7-pentamethyl-2,3-dihydrobenzofuran-5-yl)sulfonyl)guanidino)pentan-2-yl)carbamate (Fmoc-Arg(Pbf)-Coumarin)

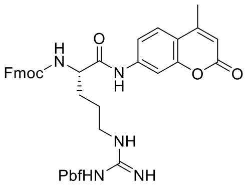

Chemical Formula: $C_{44}H_{47}N_5O_8S$, **Exact Mass**: 805.32, **Molecular Weight**: 805.95.

7-amino-4-methylcoumarin (0.28 mmol) and Fmoc-Arg(Pbf)-OH (0.28 mmol) were dissolved in pyridine (1.6 mL) and cooled to 0 °C. $POCl_3$ (0.052 mL) was added dropwise, the reaction was allowed to warm to room temperature and stirred for 1 hour. The reaction mixture was quenched with 10 mL H_2O and extracted with EtOAc (3x). The organic layers were washed with 2 M HCl (1x), 5% $NaHCO_3$ (1x) and brine (1x), dried on sodium sulfate and concentrated under *vacuo*. The crude product was purified by reverse phase chromatography (Biotage, 48g C18 column, 10-100% ACN in H_2O) to yield Fmoc-Arg(Pbf)-Coumarin (0.112 mmol, 40%).

LCMS (ESI); RT= 3.03, $[M+1H]^{1+}$: 806.13.

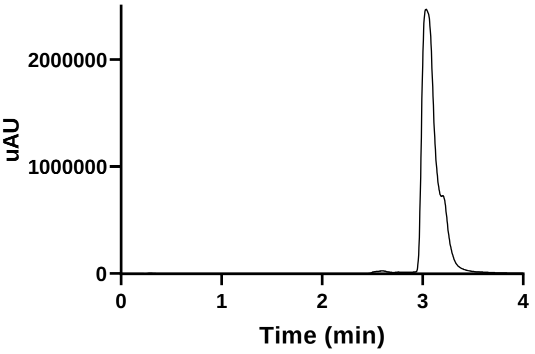

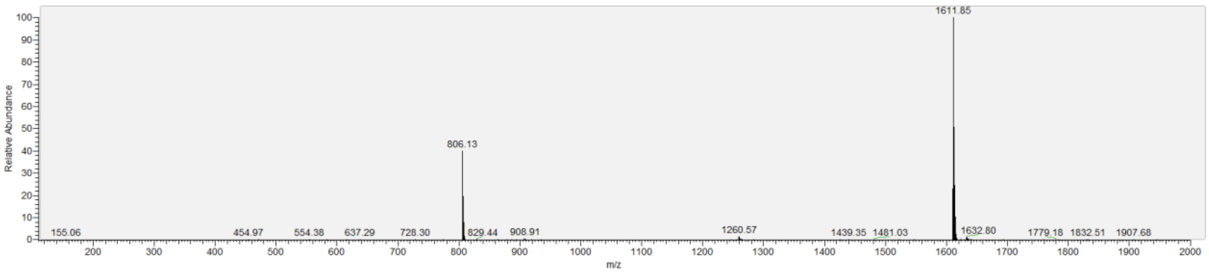

NMR; ^{1}H NMR (400 MHz, DMSO) δ 10.49 (s, 1H), 7.88 (d, *J* = 7.5 Hz, 2H), 7.80 – 7.69 (m, 5H), 7.49 (dd, *J* = 8.7, 2.1 Hz, 1H), 7.40 (t, *J* = 7.5 Hz, 2H), 7.31 (tt, *J* = 7.4, 1.6 Hz, 2H), 6.27 (d, *J* = 1.4 Hz, 1H), 4.28 (d, *J* = 8.0 Hz, 2H), 4.22 (d, *J* = 6.8 Hz, 1H), 4.15 (td, *J* = 8.4, 5.6 Hz, 1H), 3.06 (h, *J* = 6.5 Hz, 2H), 2.89 (s, 2H), 2.45 (s, 3H), 2.41 – 2.37 (m, 6H), 1.95 (s, 3H), 1.76 – 1.56 (m, 2H), 1.50 (t, *J* = 7.6 Hz, 2H), 1.37 (s, 6H).

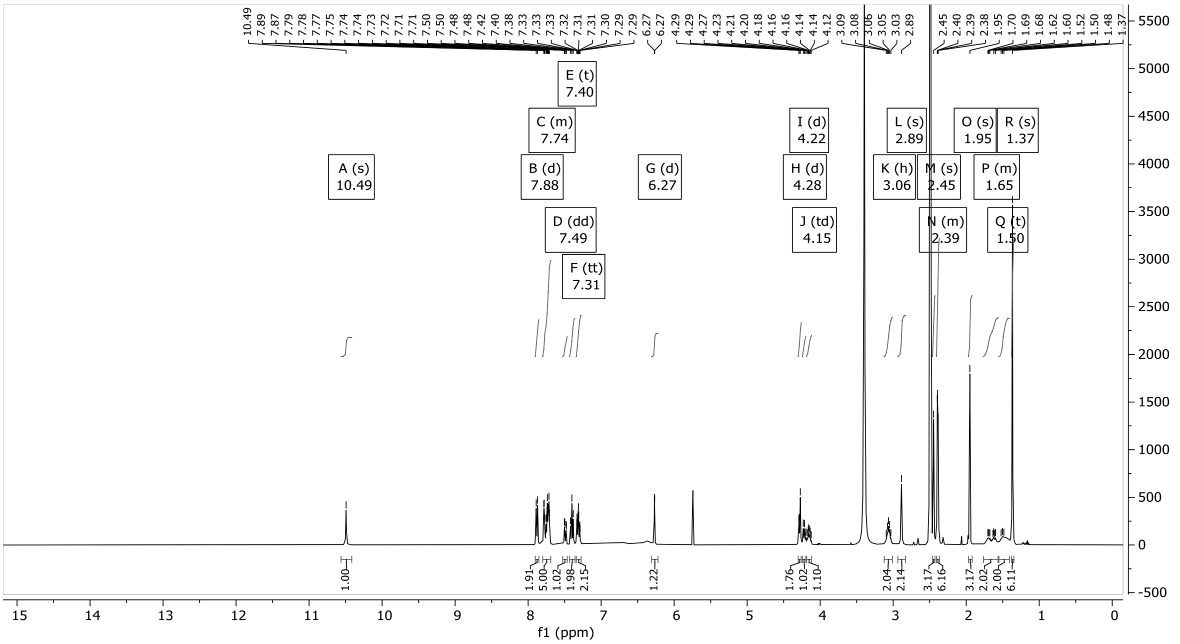

(9H-fluoren-9-yl)methyl (S)-2-(((S)-1-((4-methyl-2-oxo-2H-chromen-7-yl)amino)-1-oxo-5-(3-((2,2,4,6,7-pentamethyl-2,3-dihydrobenzofuran-5-yl)sulfonyl)guanidino)pentan-2-yl)carbamoyl)pyrrolidine-1-carboxylate (Fmoc-Pro-Arg(Pbf)-Coumarin)

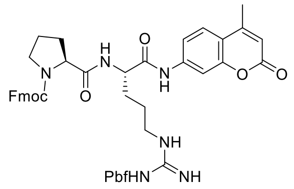

Chemical Formula: $C_{49}H_{54}N_6O_9S$, **Exact Mass**: 902.37, **Molecular Weight**: 903.06.

Fmoc-Arg(Pbf)-Coumarin (0.11 mmol) was treated with DEA (1 mL) in acetonitrile (1 mL) for 30 minutes. The reaction mixture was concentrated under *vacuo*, redissolved in acetonitrile (5 mL) and reconcentrated (2x). The crude Arg(Pbf)-coumarin was dissolved in 0.3 mL dry DMF.

Fmoc-Pro-OH (0.12 mmol), HATU (0.11 mmol) and DIPEA (0.33 mmol) were dissolved in dry DMF (0.8 mL) and stirred at 0 °C for 15 minutes. Arg(Pbf)-coumarin (0.11 mmol) in 0.3 mL dry DMF was added dropwise. The reaction mixture was allowed to warm to room temperature and stirred for 1 hour. The reaction mixture was quenched with 10 mL H_2O and extracted with EtOAc (3x). The organic layers were washed with 2 M HCl (1x), 5% $NaHCO_3$ (1x) and brine (1x), dried on sodium sulfate and concentrated under *vacuo*. The crude product was purified by reverse phase chromatography (Biotage, 48g C18 column, 10-100% ACN in H_2O) to yield Fmoc-Pro-Arg(Pbf)-Coumarin (0.070 mmol, 64%).

LCMS (ESI); RT= 2.89, $[M+1H]^{1+}$: 903.17.

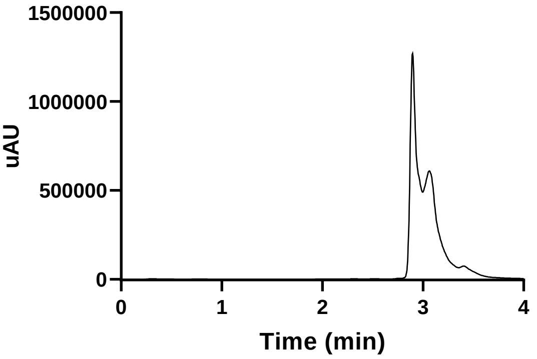

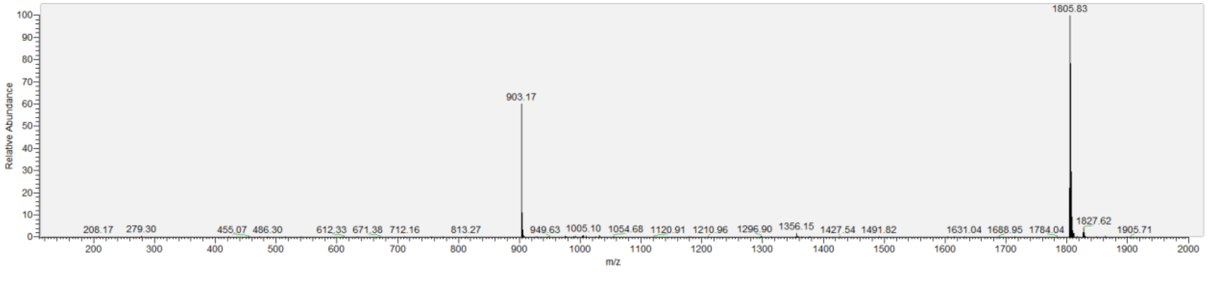

NMR; ^{1}H NMR (400 MHz, DMSO) δ 7.91 – 7.88 (m, 1H), 7.82 – 7.77 (m, 1H), 7.73 – 7.61 (m, 3H), 7.51 (ddd, *J* = 8.8, 5.4, 3.3 Hz, 1H), 7.44 – 7.26 (m, 5H), 6.29 – 6.22 (m, 1H), 4.47 (dt, *J* = 9.0, 4.5 Hz, 1H), 4.41 – 4.30 (m, 1H), 4.30 – 4.10 (m, 3H), 3.47 (s, 3H), 3.06 (s, 2H), 2.87 (d, *J* = 12.4 Hz, 2H), 2.44 (s,

2H), 2.41 – 2.37 (m, 5H), 2.34 (s, 2H), 1.94 (d, *J* = 11.5 Hz, 3H), 1.88 – 1.67 (m, 4H), 1.54 (d, *J* = 62.5 Hz, 3H), 1.37 (s, 6H).

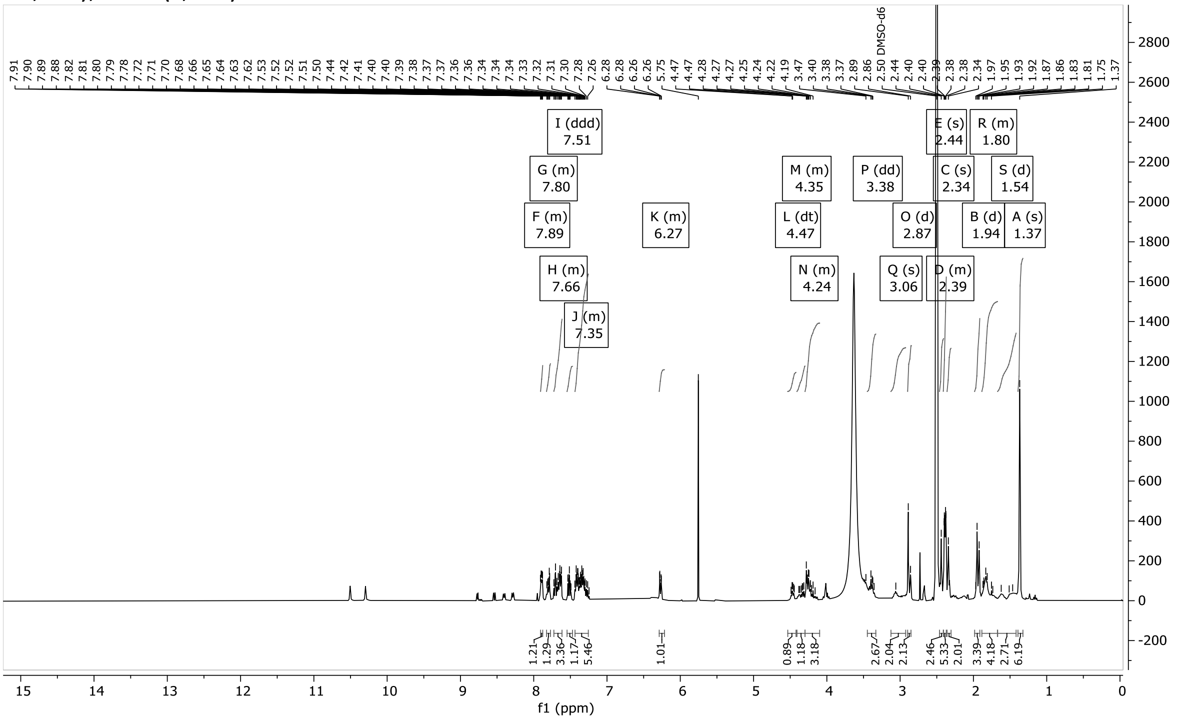

(S)-1-(D-phenylalanyl)-N-((S)-5-guanidino-1-((4-methyl-2-oxo-2H-chromen-7-yl)amino)-1-oxopentan-2-yl)pyrrolidine-2-carboxamide (phe-Pro-Arg-Coumarin)

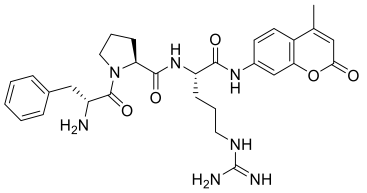

Chemical Formula: $C_{30}H_{37}N_7O_5$, **Exact Mass**: 575.29, **Molecular Weight**: 575.67.

Fmoc-Pro-Arg(Pbf)-Coumarin (0.066 mmol) was treated with DEA (1.5 mL) in acetonitrile (1.5 mL) for 30 minutes. The reaction mixture was concentrated under *vacuo*, redissolved in acetonitrile (5 mL) and reconcentrated (2x). The crude Pro-Arg(Pbf)-coumarin was dissolved in 0.2 mL dry DMF.

Fmoc-d-phe-OH (0.073 mmol), HATU (0.066 mmol) and DIPEA (0.198 mmol) were dissolved in dry DMF (0.5 mL) and stirred at 0 °C for 15 minutes. Pro-Arg(Pbf)-coumarin (0.066 mmol) in 0.2 mL dry DMF was added dropwise. The reaction mixture was allowed to warm to room temperature and stirred for 1 hour. The reaction mixture was quenched with 10 mL H_2O and extracted with EtOAc (3x). The organic layers were washed with 2 M HCl (1x), 5% $NaHCO_3$ (1x) and brine (1x), dried on sodium sulfate and concentrated under *vacuo*.

The crude protected peptide was dissolved in 50:50 TFA:DCM and stirred at room temperature for 2 hours. The mixture was concentrated under *vacuo* and azeotroped with toluene three times. The peptide was then dissolved in 20% piperidine in DMF and stirred for 30 minutes. The mixture was purified by reverse phase chromatography (Biotage, 48g C18 column, 10-100% ACN in H_2O) to yield phe-Pro-Arg(Pbf)-Coumarin (0.015 mmol, 23%).

LCMS (ESI); RT= 1.54, $[M+1H]^{1+}$: 576.26.

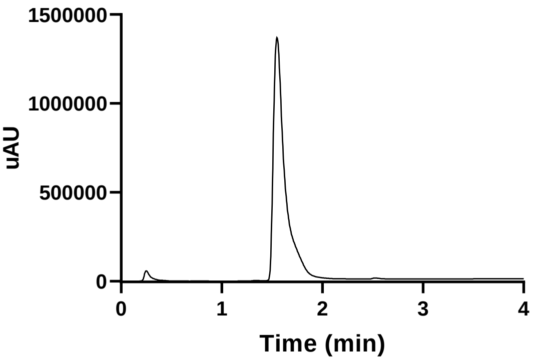

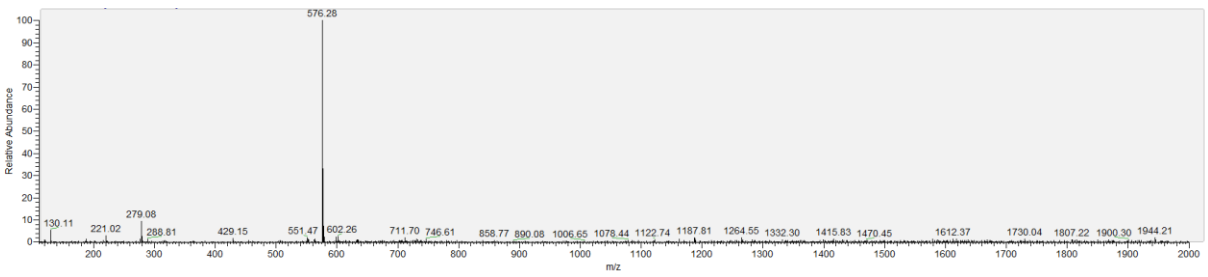

NMR; ^{1}H NMR (400 MHz, DMSO) δ 10.41 (s, 1H), 8.37 – 8.29 (m, 1H), 8.24 (s, 2H), 7.79 – 7.77 (m, 1H), 7.74 – 7.70 (m, 1H), 7.58 – 7.52 (m, 1H), 7.50 – 7.45 (m, 1H), 7.41 – 7.28 (m, 4H), 7.26 – 7.22 (m, 2H), 6.29 (s, 1H), 4.49 – 4.23 (m, 3H), 3.58 – 3.46 (m, 2H), 3.19 – 3.04 (m, 3H), 3.02 – 2.92 (m, 1H), 2.85 – 2.69 (m, 1H), 2.41 (s, 3H), 1.85 – 1.70 (m, 4H), 1.69 – 1.56 (m, 2H), 1.55 – 1.42 (m, 2H).

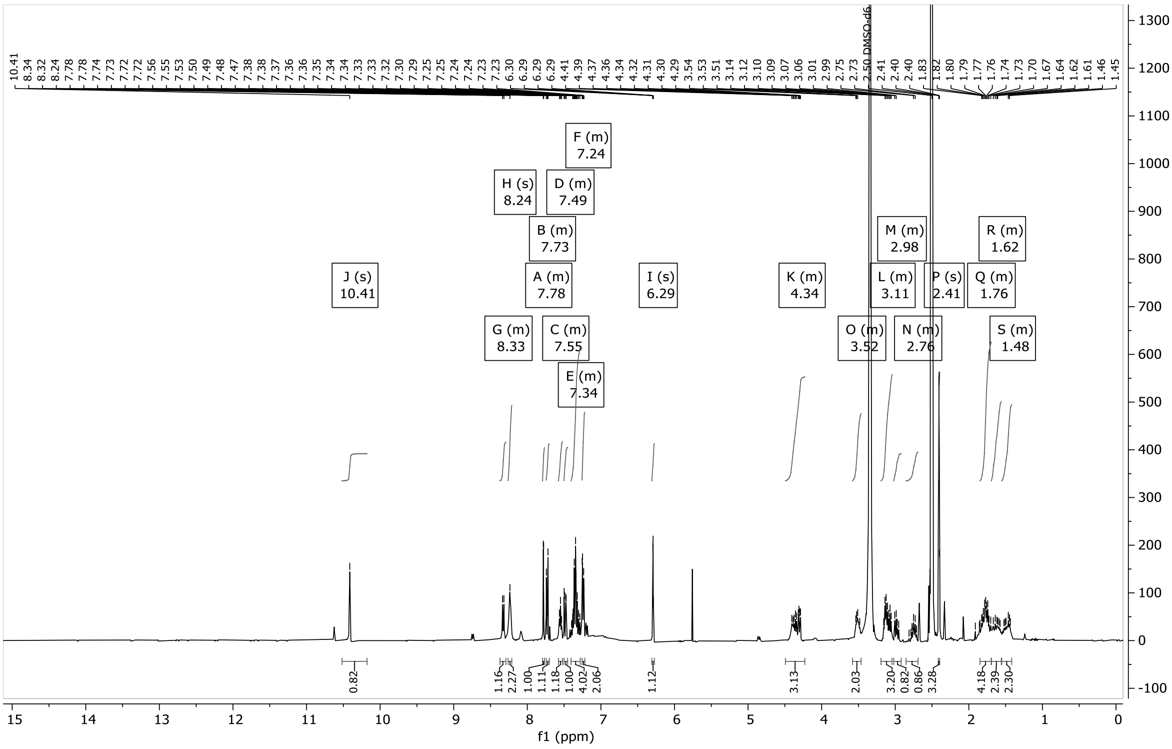

^{13}C NMR (126 MHz, DMSO) δ 170.96, 170.77, 166.24, 159.68, 156.31, 153.32, 152.77, 141.67, 134.15, 129.22, 128.26, 127.17, 125.69, 114.98, 114.90, 112.13, 105.43, 59.07, 53.17, 51.61, 46.95, 46.51, 40.12, 39.78, 36.47, 28.99, 28.55, 24.94, 23.48, 17.67.

4. Characterisation of PNA-peptide compounds

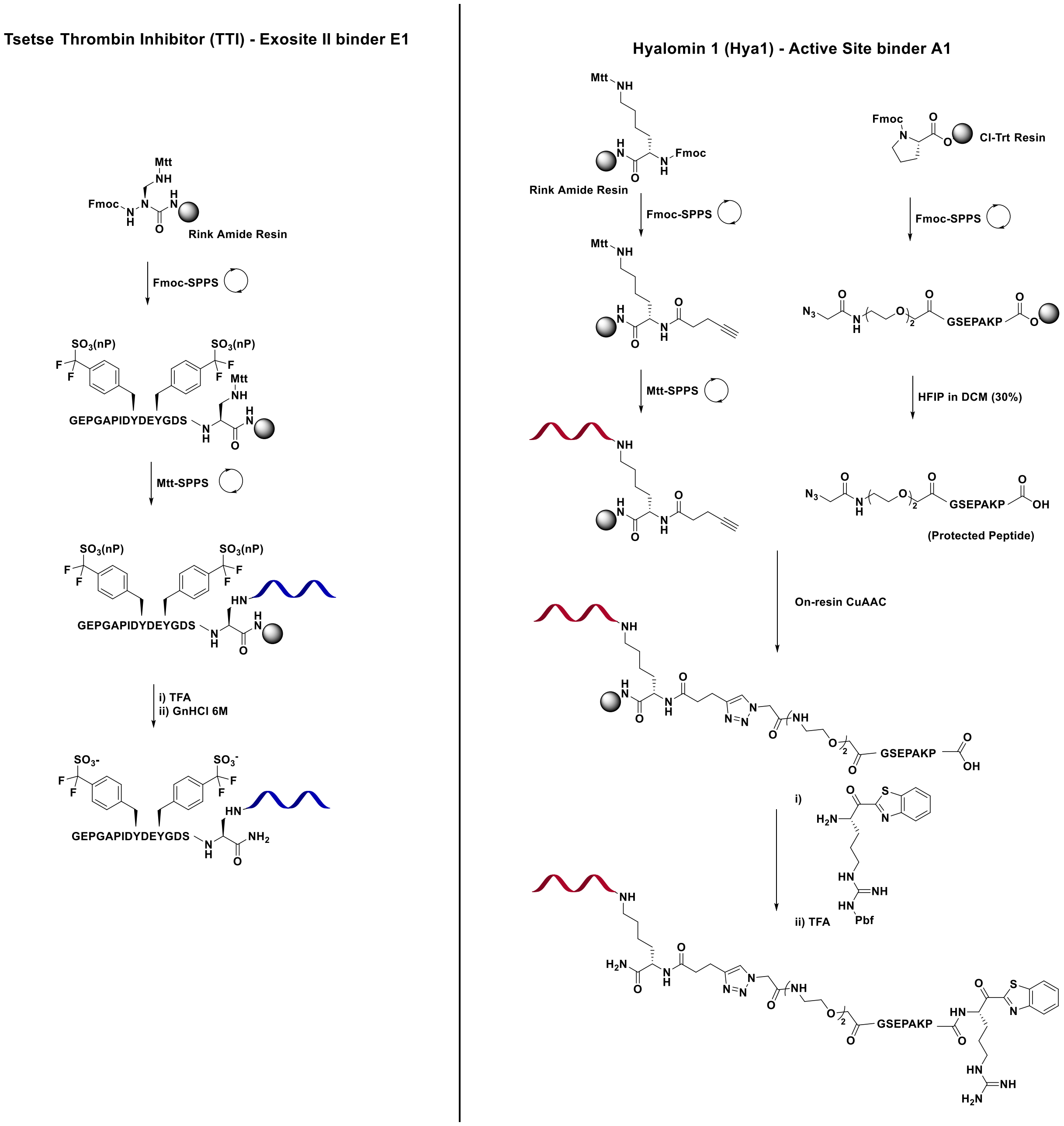

Supplementary Figure 1: General Scheme for the synthesis of the compounds. The general methods and synthetic procedures are described in the Online Methods section.

i) Inhibitors

A1

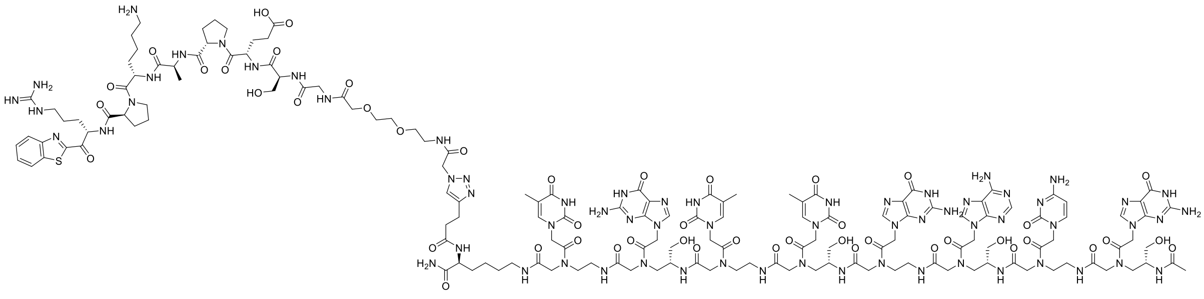

Chemical Formula: $C_{154}H_{211}N_{65}O_{48}S$, **Exact Mass**: 3770.58, **Molecular Weight**: 3772.85.

MALDI-TOF; m/z found: 3772.40.

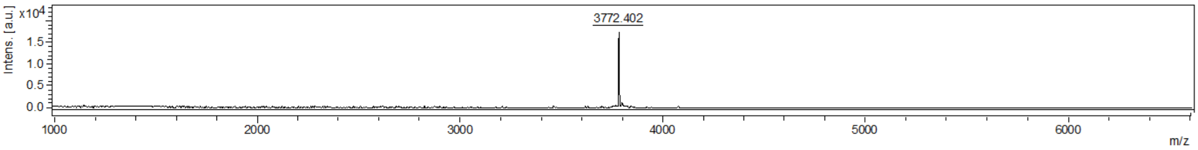

LCMS (ESI); RT= 1.44 min, $[M+3H]^{3+}$: 1258.42, $[M+4H]^{4+}$: 944.25, $[M+5H]^{5+}$: 755.50, $[M+6H]^{6+}$: 629.92.

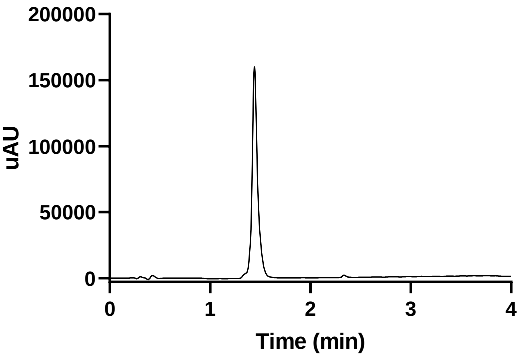

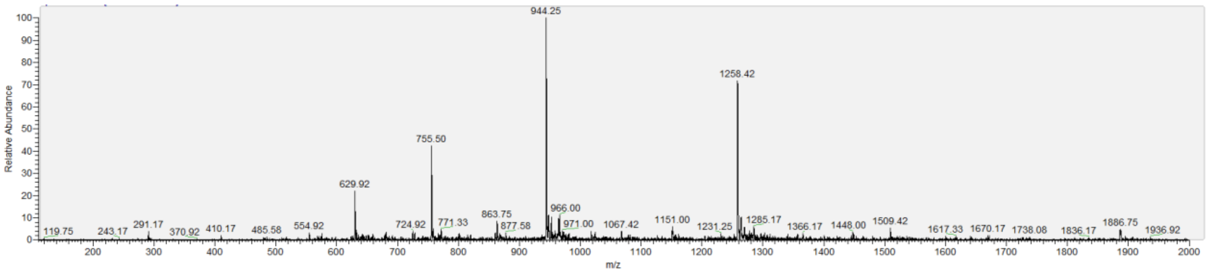

HRMS; $[M+4H]^{4+}$: 943.8892, $[M+5H]^{5+}$: 755.5194.

<u>E1</u>

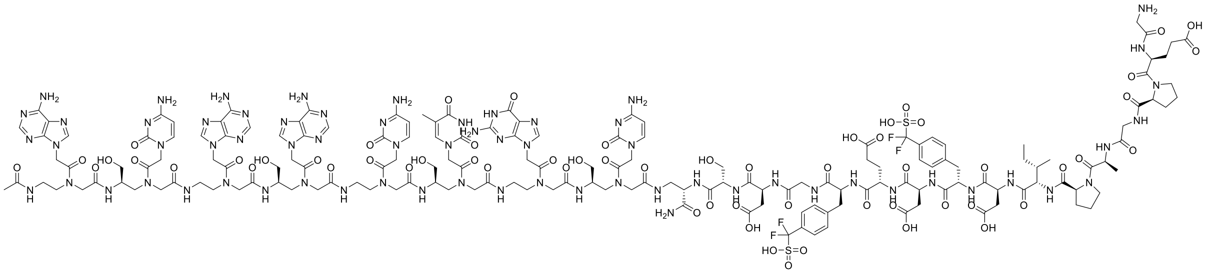

Chemical Formula: $C_{164}H_{215}F_4N_{65}O_{60}S_2$, **Exact Mass**: 4194.52, **Molecular Weight**: 4197.03.

MALDI-TOF; m/z found: 4197.52.

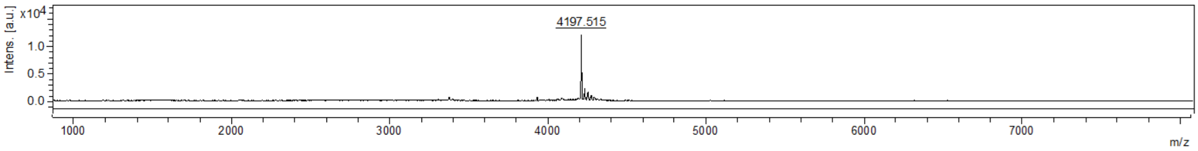

LCMS (ESI); RT= 1.38 min, $[M+3H]^{3+}$: 1399.75, $[M+4H]^{4+}$: 1050.25.

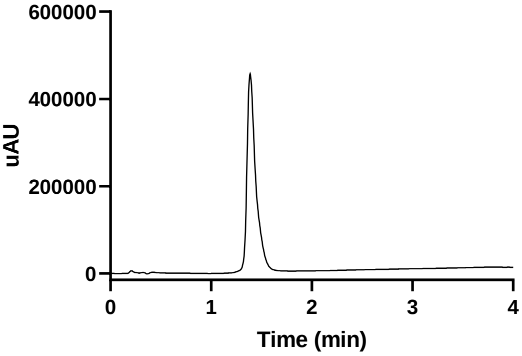

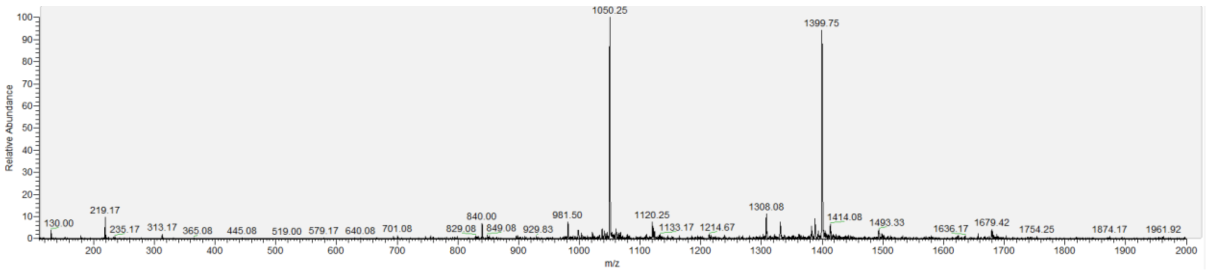

HRMS; [M+3H]$^{3+}$: 1399.8491, [M+4H]$^{4+}$: 1050.3862.

ii) Variation of PNA length

E2

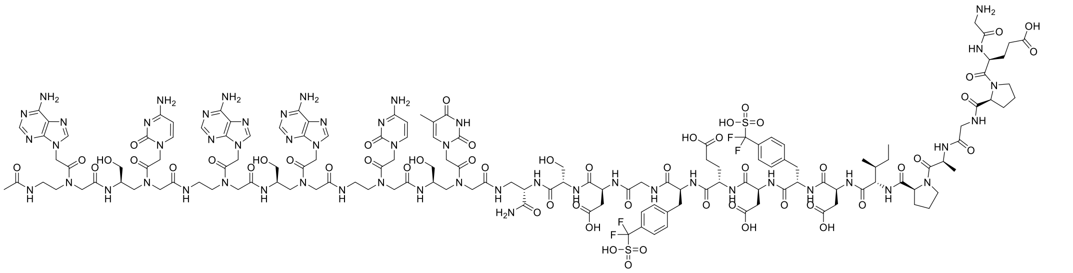

Chemical Formula: $C_{142}H_{187}F_4N_{53}O_{53}S_2$, **Exact Mass**: 3622.29, **Molecular Weight**: 3624.49

MALDI-TOF; m/z found: 3624.26

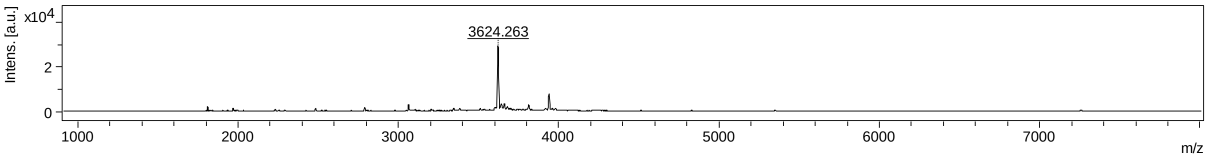

LCMS (ESI); RT= 1.36 min, [M+3H]$^{3+}$: 1208.75, [M+4H]$^{4+}$: 906.83

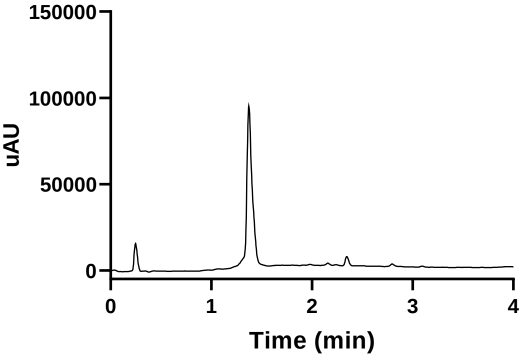

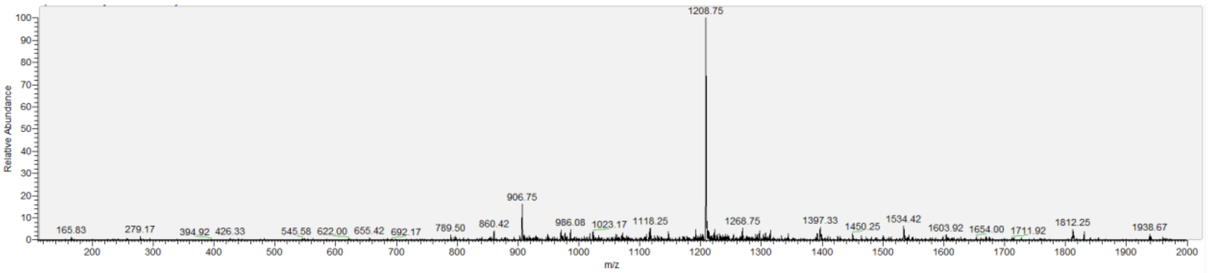

A2

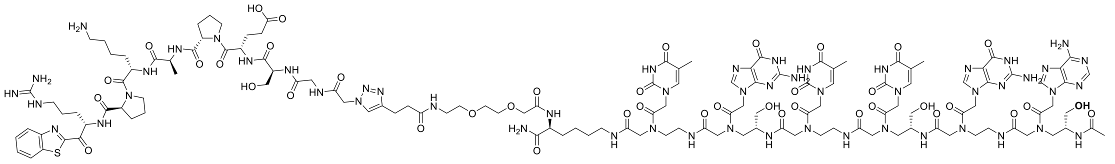

Chemical Formula: $C_{132}H_{182}N_{52}O_{42}S$, **Exact Mass**: 3199.34, **Molecular Weight**: 3201.29

MALDI-TOF; m/z found: 3201.17.

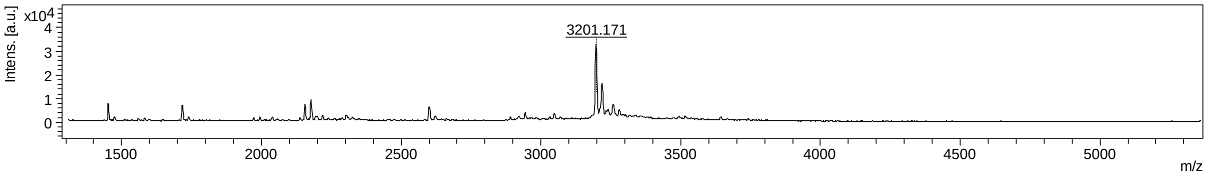

LCMS (ESI); RT= 1.48 min, $[M+2H]^{2+}$: 1600.75, $[M+3H]^{3+}$: 1067.83, $[M+4H]^{4+}$: 801.00, $[M+5H]^{5+}$: 641.17.

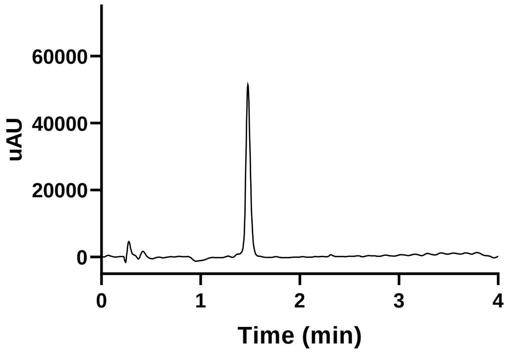

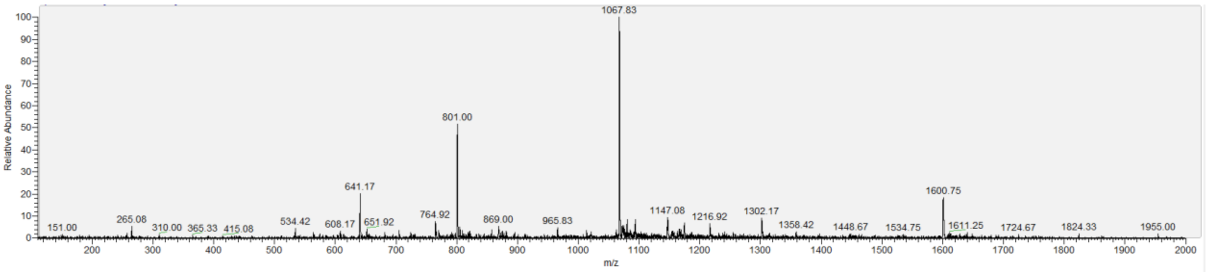

E3

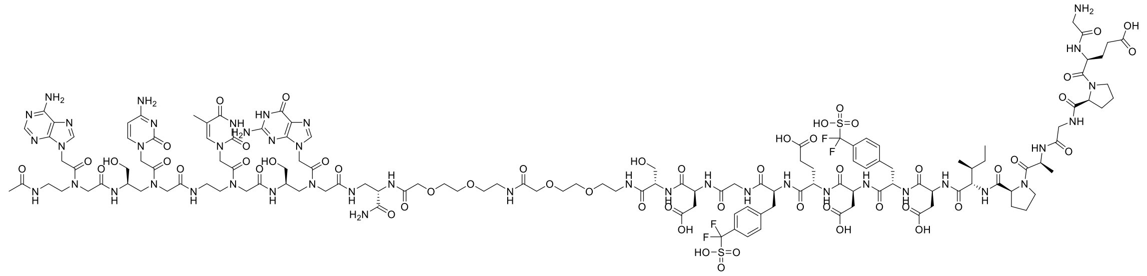

Chemical Formula: $C_{132}H_{181}F_4N_{43}O_{54}S_2$, **Exact Mass**: 3372.21, **Molecular Weight**: 3374.26.

MALDI-TOF; m/z found: 3373.96.

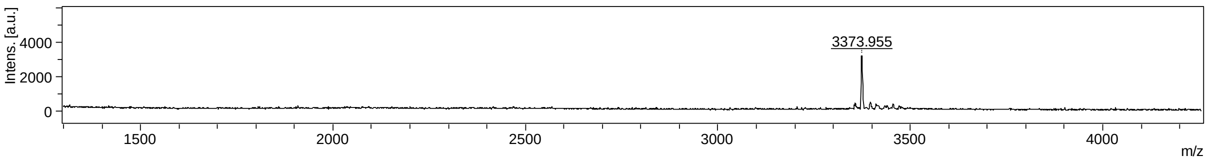

LCMS (ESI); RT= 1.42 min, $[M+2H]^{2+}$: 1687.58, $[M+3H]^{3+}$: 1125.33.

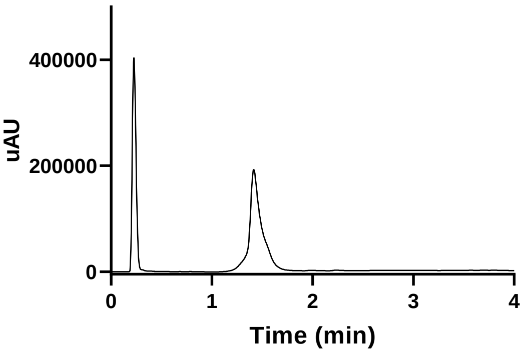

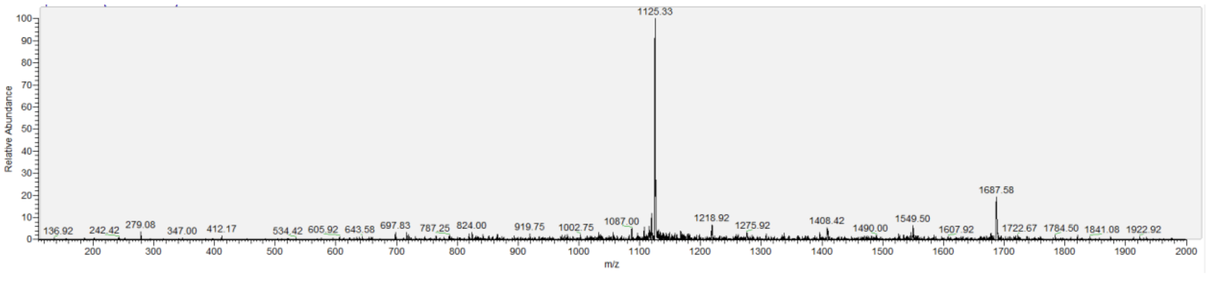

A3

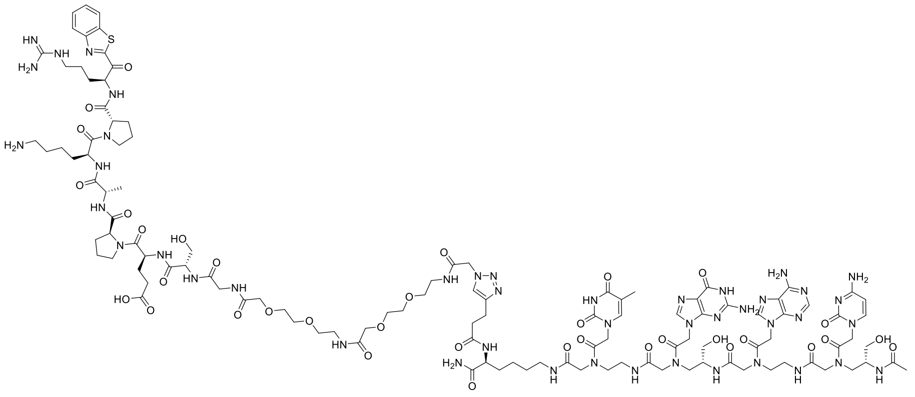

Chemical Formula: $C_{114}H_{164}N_{44}O_{35}S$, **Exact Mass**: 2741.21, **Molecular Weight**: 2742.90.

MALDI-TOF; m/z found: 2743.43.

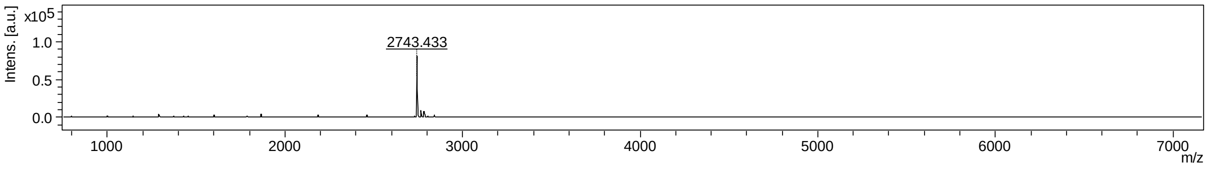

LCMS (ESI); RT= 1.44 min, $[M+2H]^{2+}$: 1372.00, $[M+3H]^{3+}$: 915.17, $[M+4H]^{4+}$: 686.58, $[M+5H]^{5+}$: 549.50.

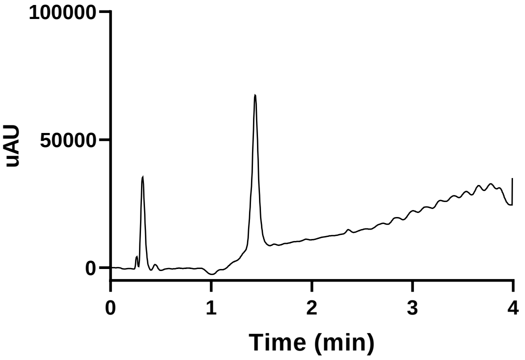

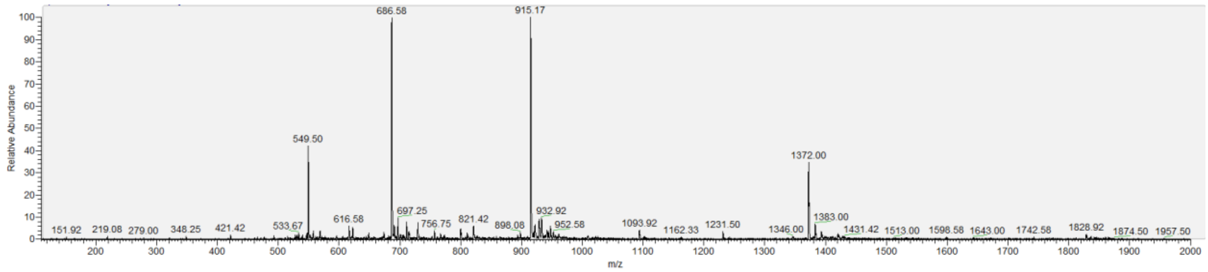

A8

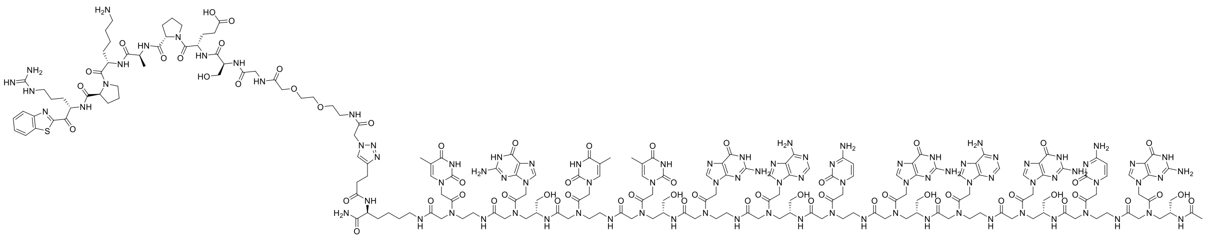

Chemical Formula: $C_{199}H_{267}N_{91}O_{61}S$, **Exact Mass**: 4939.03, **Molecular Weight**: 4941.96.

MALDI-TOF; m/z found: 4942.01.

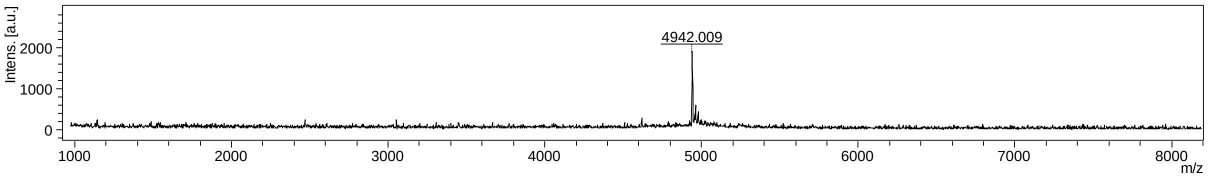

LCMS (ESI); RT= 1.49 min, $[M+3H]^{3+}$: 1647.67, $[M+4H]^{4+}$: 1236.25, $[M+5H]^{5+}$: 969.17, $[M+6H]^{6+}$: 824.50, $[M+7H]^{7+}$:706.67.

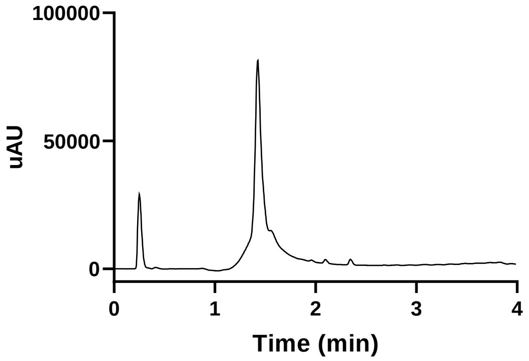

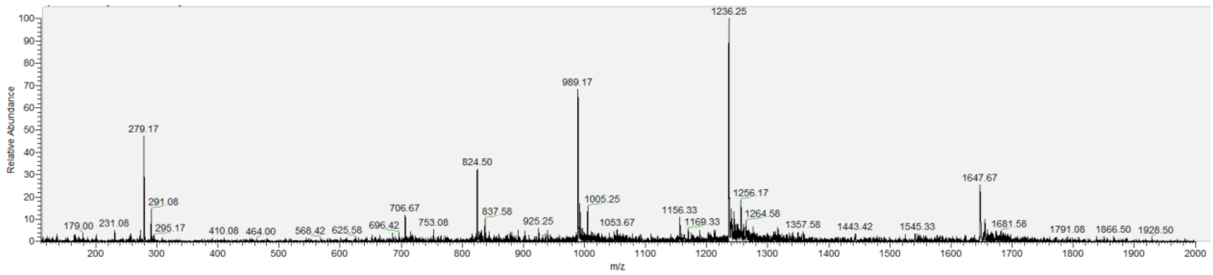

HRMS; $[M+3H]^{3+}$: 1648.3586, $[M+4H]^{4+}$: 1236.2682, $[M+5H]^{5+}$: 989.2154, $[M+6H]^{6+}$: 824.5084, $[M+7H]^{7+}$:706.8661.

iii) Charge modification on exosite II binder

E4

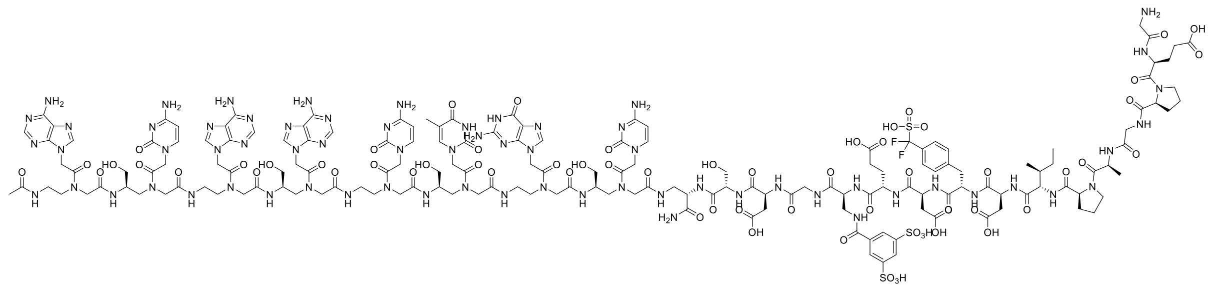

Chemical Formula: $C_{164}H_{216}F_2N_{66}O_{64}S_3$, **Exact Mass**: 4267.48, **Molecular Weight**: 4270.11.

LCMS (ESI); RT= 1.48 min, $[M+3H]^{3+}$: 1424.00, $[M+4H]^{4+}$: 1068.33.

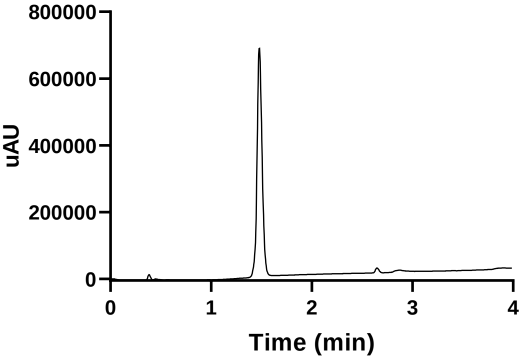

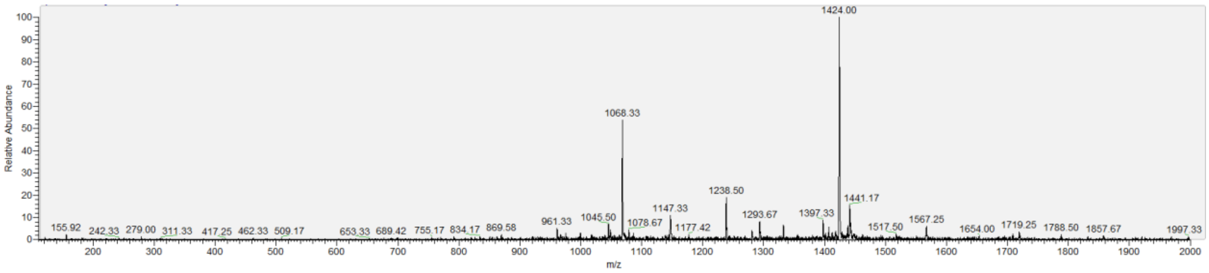

<u>**E5**</u>

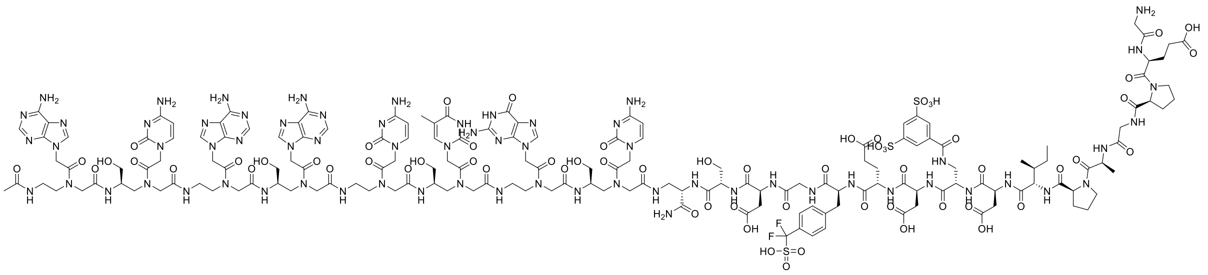

Chemical Formula: $C_{164}H_{216}F_2N_{66}O_{64}S_3$, **Exact Mass**: 4267.48, **Molecular Weight**: 4270.11.

LCMS (ESI); RT= 1.30 min, $[M+3H]^{3+}$: 1424.08, $[M+4H]^{4+}$: 1068.25.

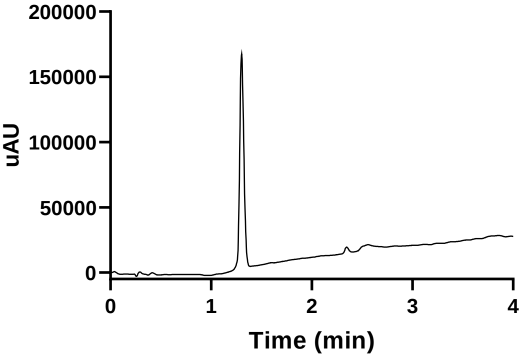

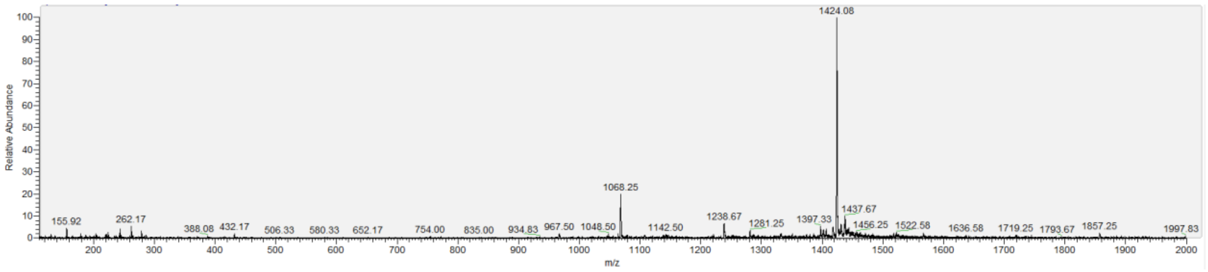

E6

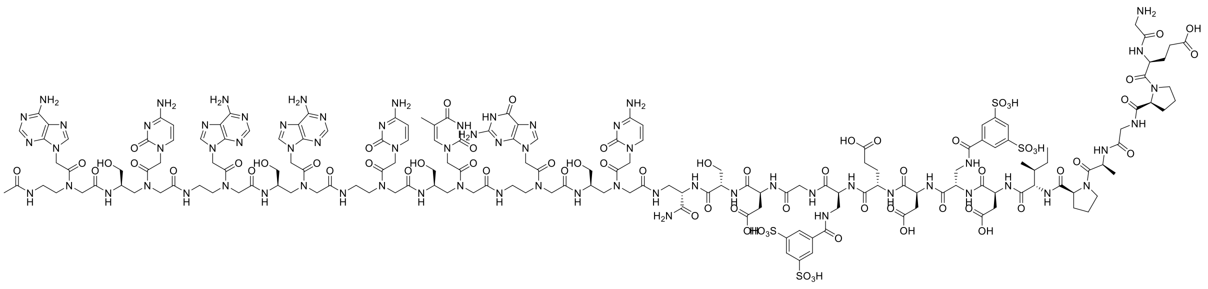

Chemical Formula: $C_{164}H_{217}N_{67}O_{68}S_4$, **Exact Mass**: 4340.45, **Molecular Weight**: 4343.18.

LCMS (ESI); RT= 1.24 min, $[M+3H]^{3+}$: 1446.33, $[M+4H]^{4+}$: 1086.50.

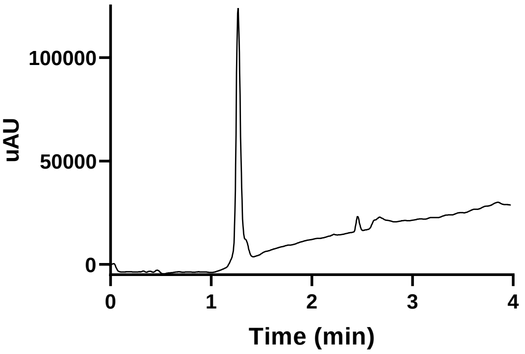

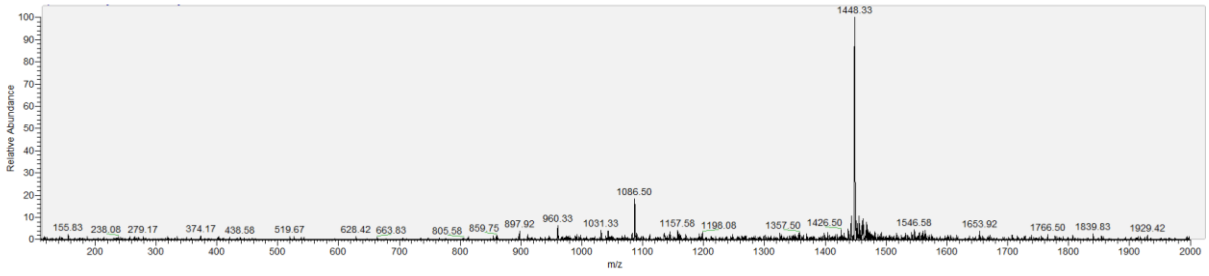

E7

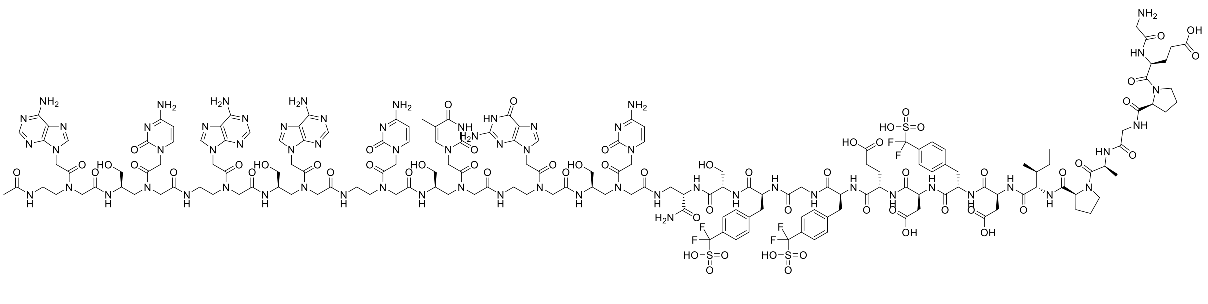

Chemical Formula: $C_{170}H_{219}F_6N_{65}O_{61}S_3$, **Exact Mass**: 4356.51, **Molecular Weight**: 4359.19

MALDI-TOF; m/z found: 4359.75

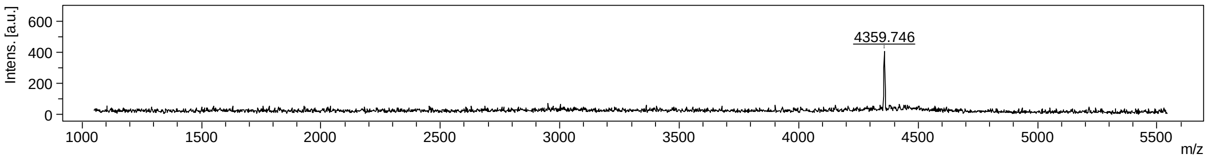

LCMS (ESI); RT= 1.42 min, $[M+3H]^{3+}$: 1453.67, $[M+4H]^{4+}$: 1090.67.

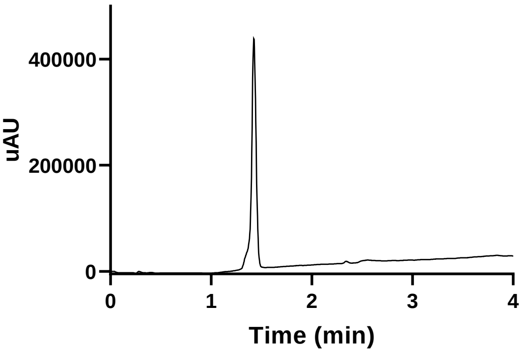

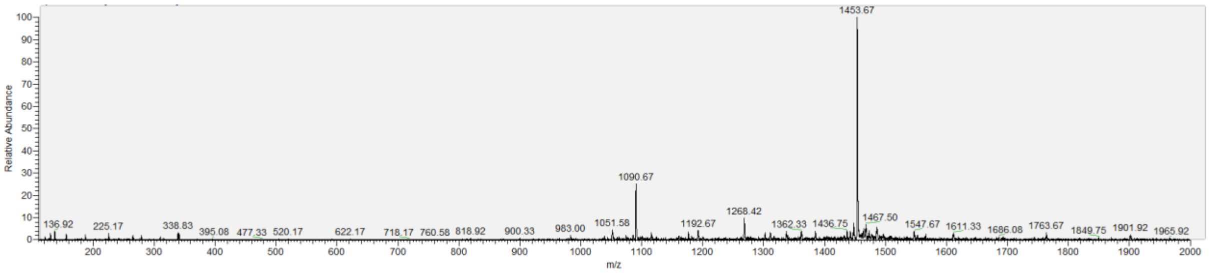

E8

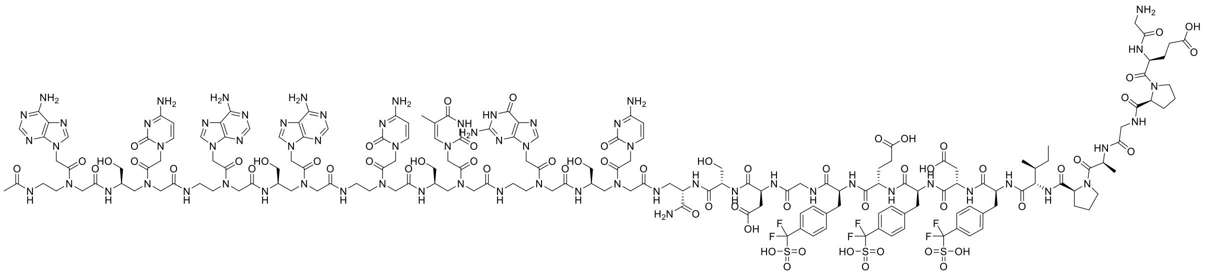

Chemical Formula: $C_{170}H_{219}F_6N_{65}O_{61}S_3$, **Exact Mass**: 4356.51, **Molecular Weight**: 4359.19

MALDI-TOF; m/z found: 4359.70

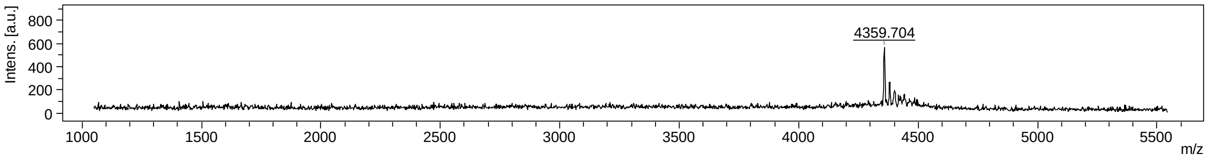

LCMS (ESI); RT= 1.46 min, $[M+3H]^{3+}$: 1453.75, $[M+4H]^{4+}$: 1090.33.

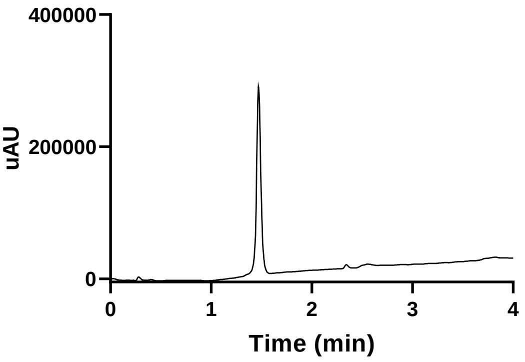

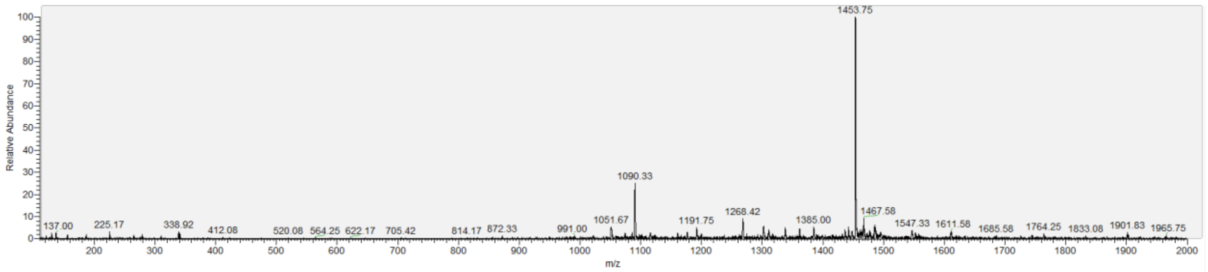

iv) Hydrophobic residues

<u>E9</u>

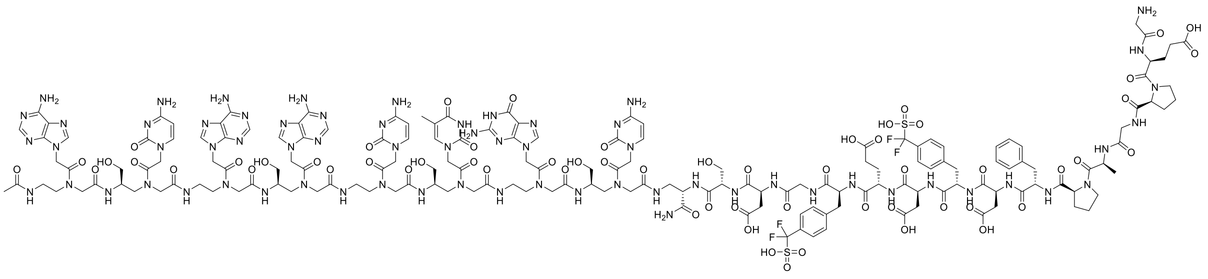

Chemical Formula: $C_{167}H_{213}F_4N_{65}O_{60}S_2$, **Exact Mass**: 4228.50, **Molecular Weight**: 4231.05.

MALDI-TOF; m/z found: 4230.99.

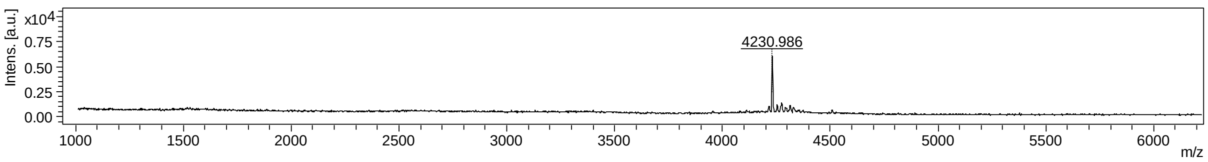

LCMS (ESI); RT= 1.48 min, $[M+3H]^{3+}$: 1411.08, $[M+4H]^{4+}$: 1058.33.

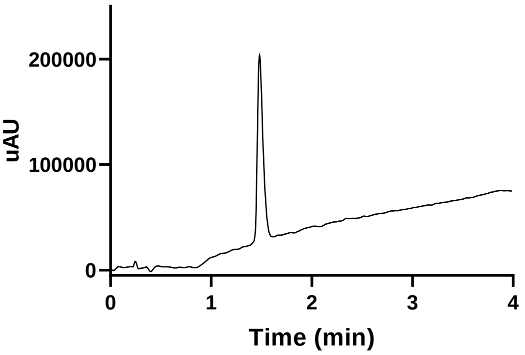

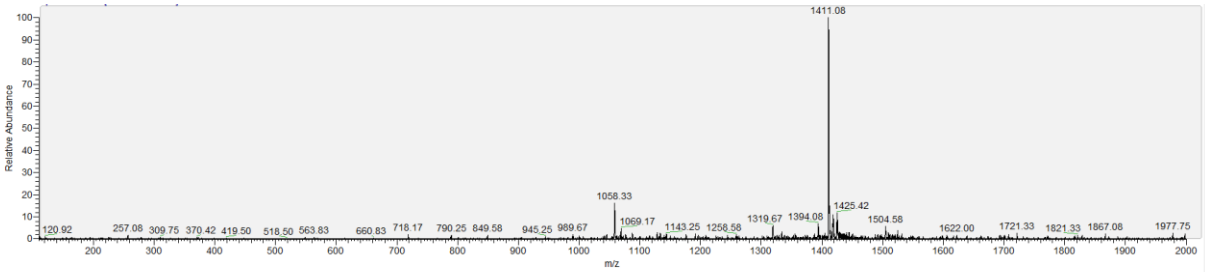

E10

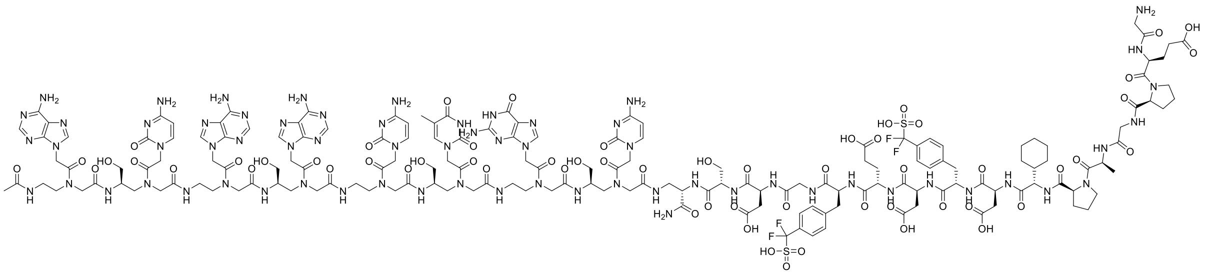

Chemical Formula: $C_{166}H_{217}F_4N_{65}O_{60}S_2$, **Exact Mass**: 4220.53, **Molecular Weight**: 4223.07.

MALDI-TOF; m/z found: 4223.40.

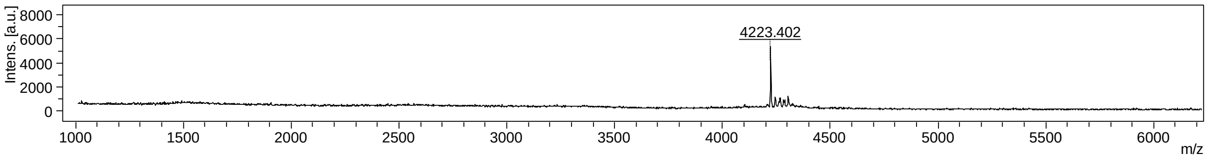

LCMS (ESI); RT= 1.48 min, $[M+3H]^{3+}$: 1408.42, $[M+4H]^{4+}$: 1056.50.

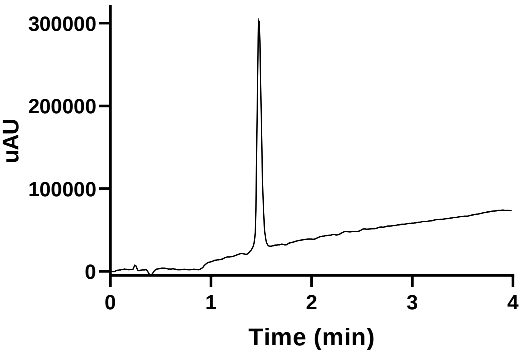

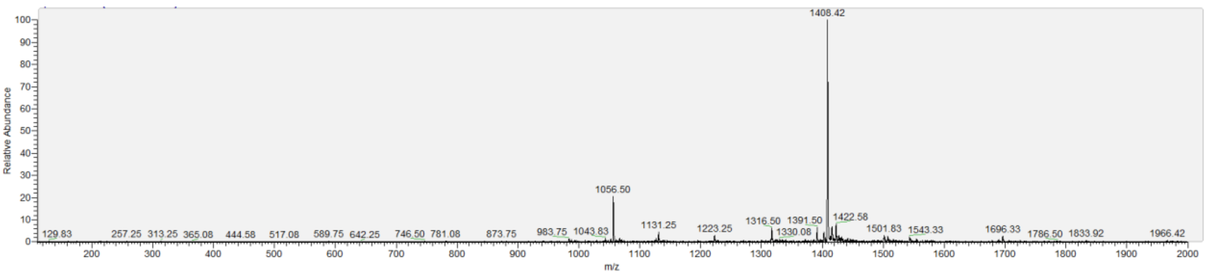

E11

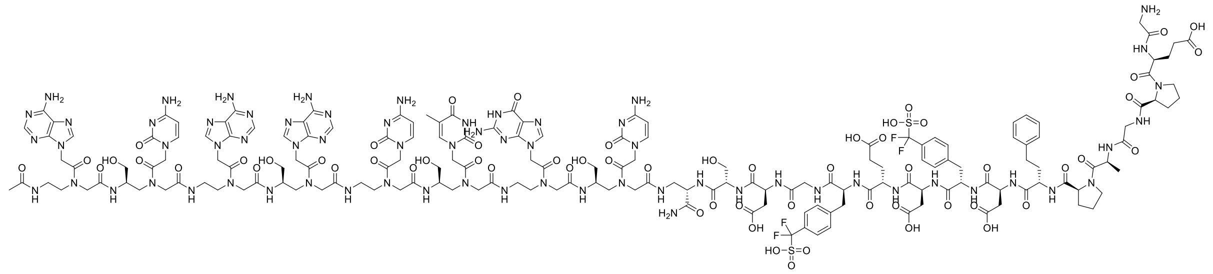

Chemical Formula: $C_{168}H_{215}F_4N_{65}O_{60}S_2$, **Exact Mass**: 4242.52, **Molecular Weight**: 4245.08.

MALDI-TOF; m/z found: 4245.59.

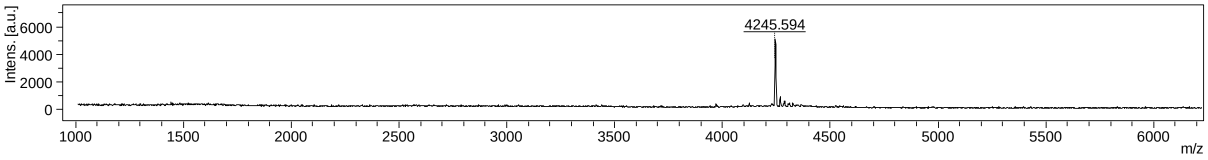

LCMS (ESI); RT= 1.51 min, $[M+3H]^{3+}$: 1415.83, $[M+4H]^{4+}$: 1062.17.

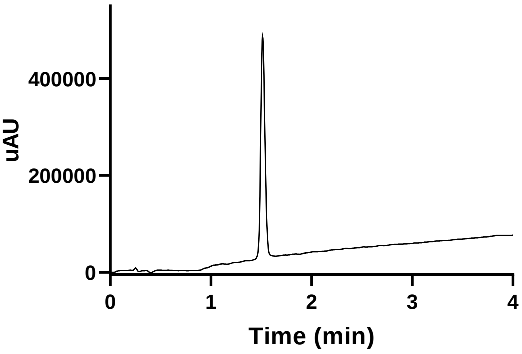

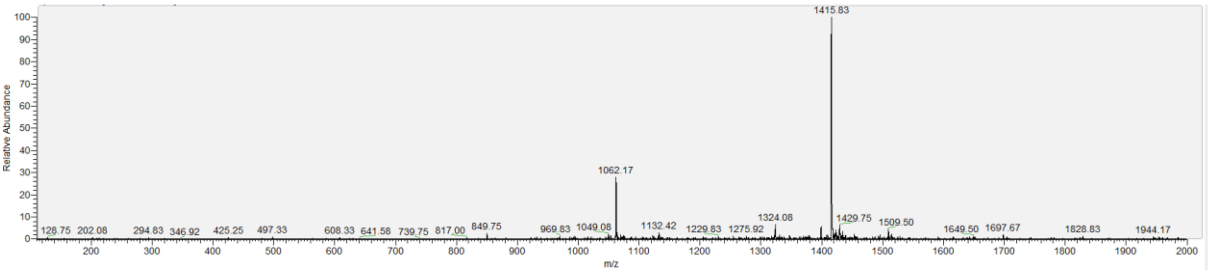

E12

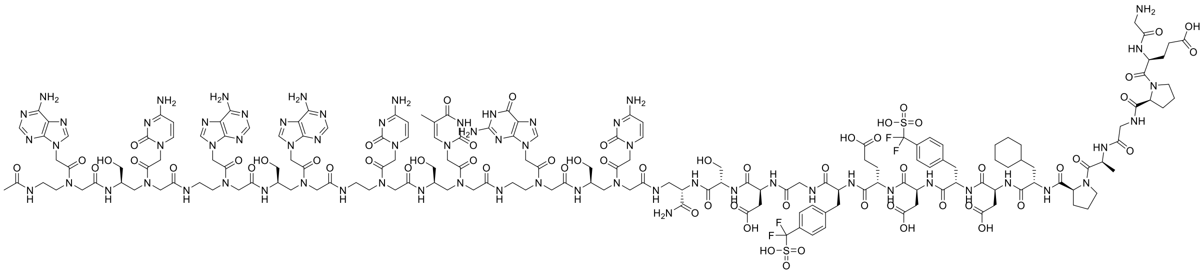

Chemical Formula: $C_{167}H_{219}F_4N_{65}O_{60}S_2$, **Exact Mass**: 4234.55, **Molecular Weight**: 4237.10

MALDI-TOF; m/z found: 4237.21.

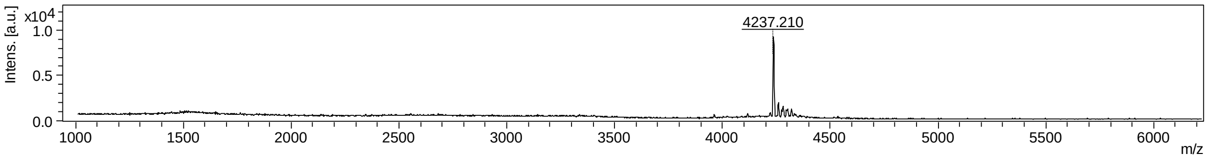

LCMS (ESI); RT= 1.56 min, $[M+3H]^{3+}$: 1413.00, $[M+4H]^{4+}$: 1059.92.

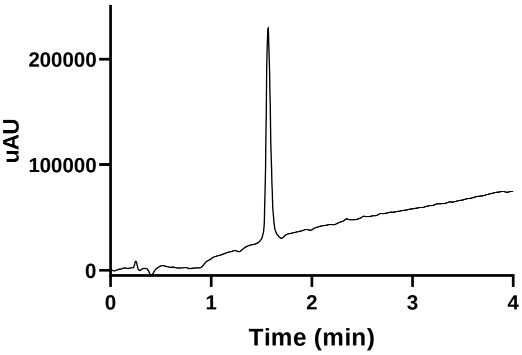

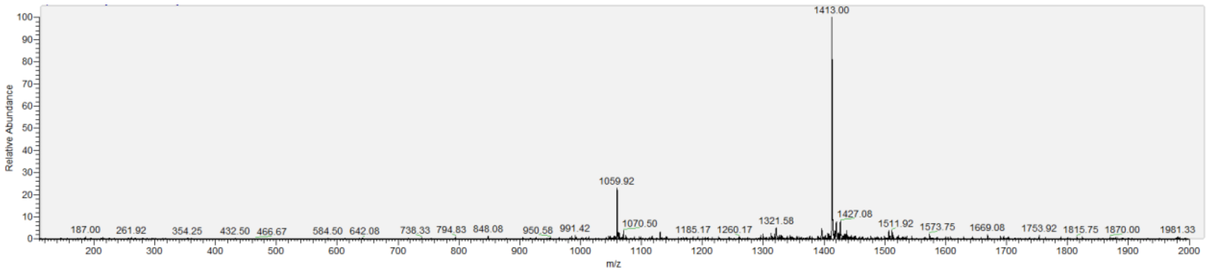

E13

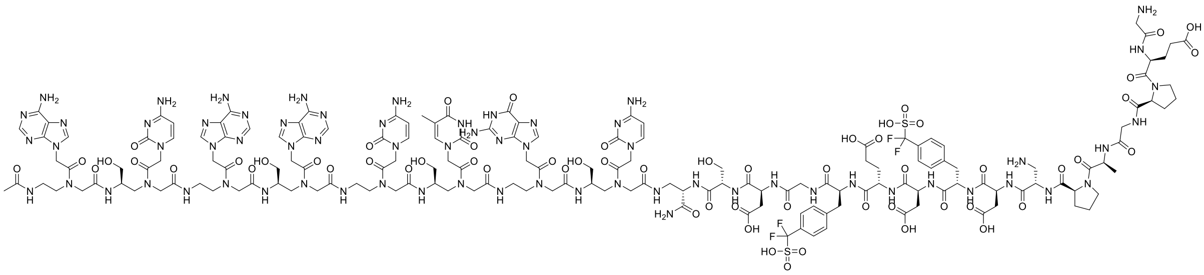

Chemical Formula: $C_{161}H_{210}F_4N_{66}O_{60}S_2$, **Exact Mass**: 4167.48, **Molecular Weight**: 4169.97.

MALDI-TOF; m/z found: 4169.95.

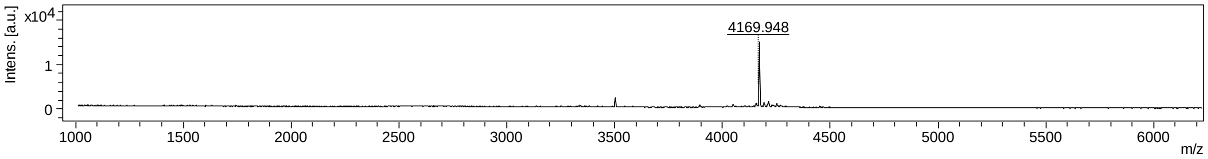

LCMS (ESI); RT= 1.35 min, $[M+3H]^{3+}$: 1390.75, $[M+4H]^{4+}$: 1043.17.

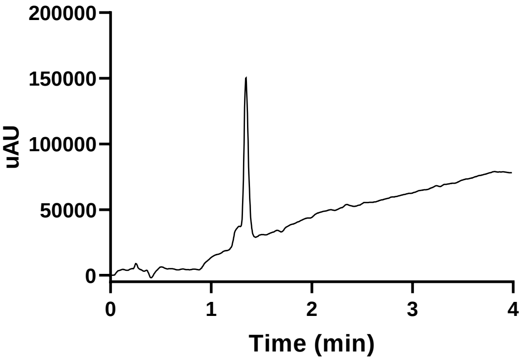

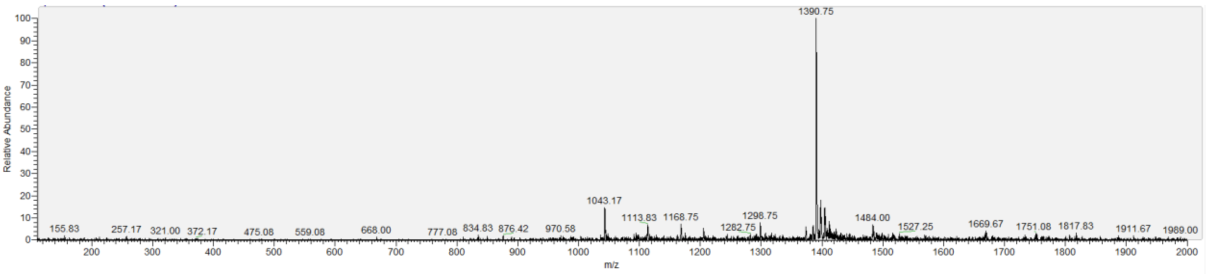

E14

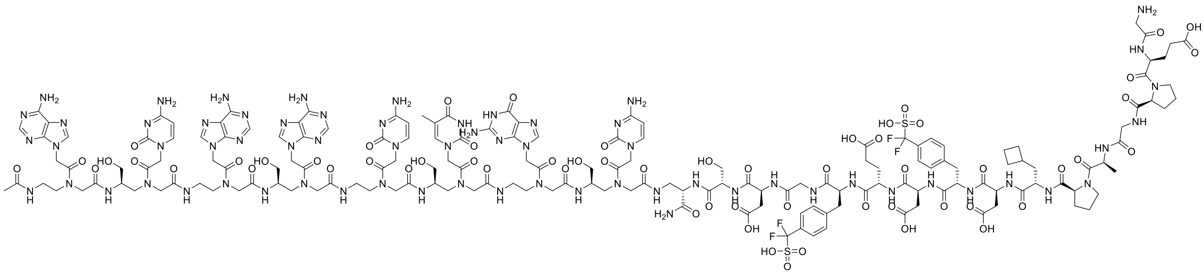

Chemical Formula: $C_{165}H_{215}F_4N_{65}O_{60}S_2$, **Exact Mass**: 4206.52, **Molecular Weight**: 4209.04.

MALDI-TOF; m/z found: 4209.36.

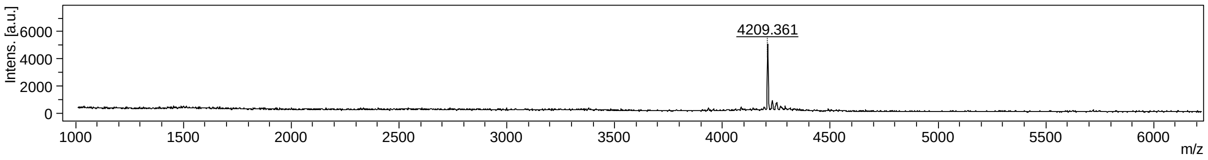

LCMS (ESI); RT= 1.46 min, $[M+3H]^{3+}$: 1403.75, $[M+4H]^{4+}$: 1052.75.

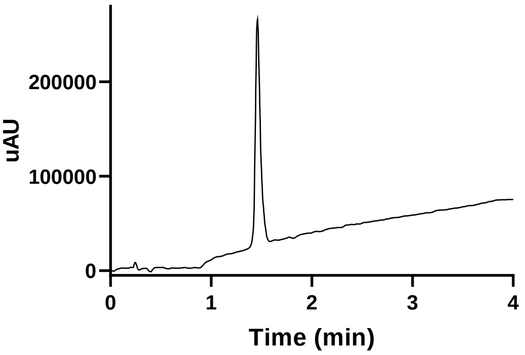

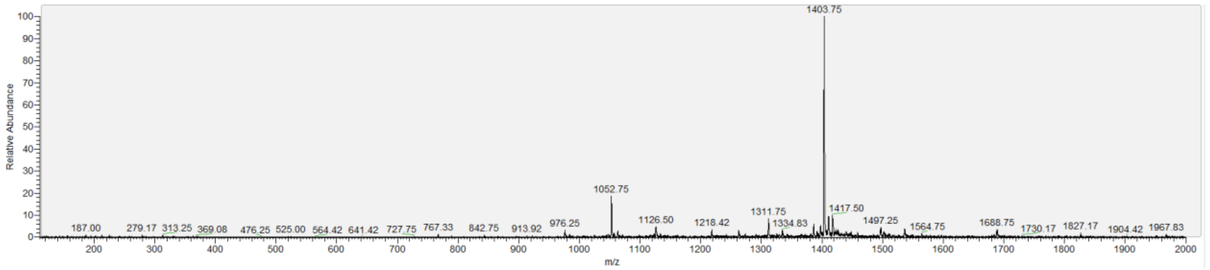

E15

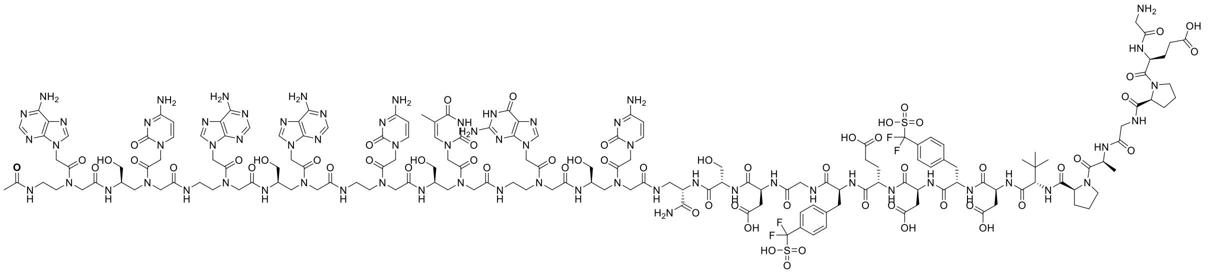

Chemical Formula: C164H215F4N65O60S2, **Exact Mass**: 4194.52, **Molecular Weight**: 4197.03.

MALDI-TOF; m/z found: 4196.96

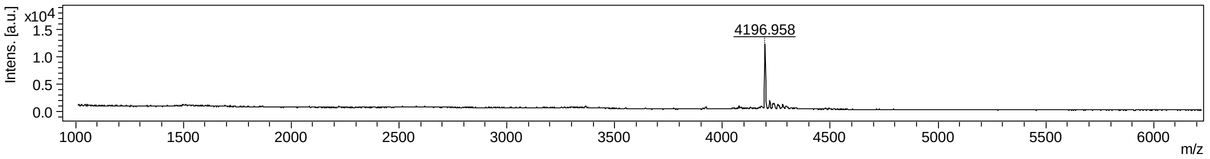

LCMS (ESI); RT= 1.42 min, $[M+3H]^{3+}$: 1399.67, $[M+4H]^{4+}$: 1050.17.

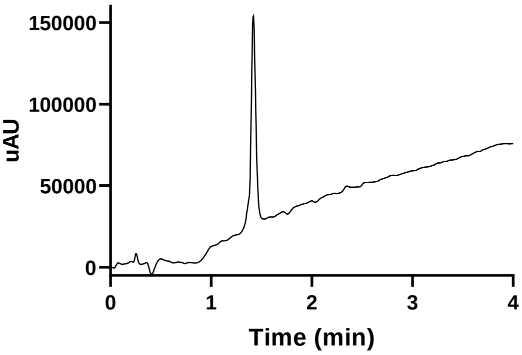

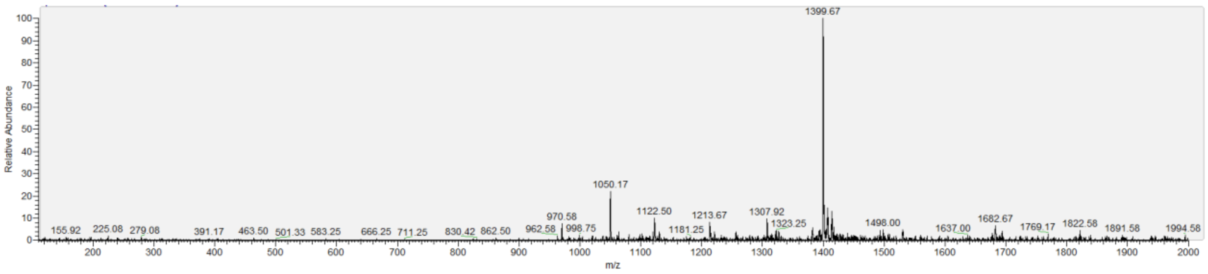

E16

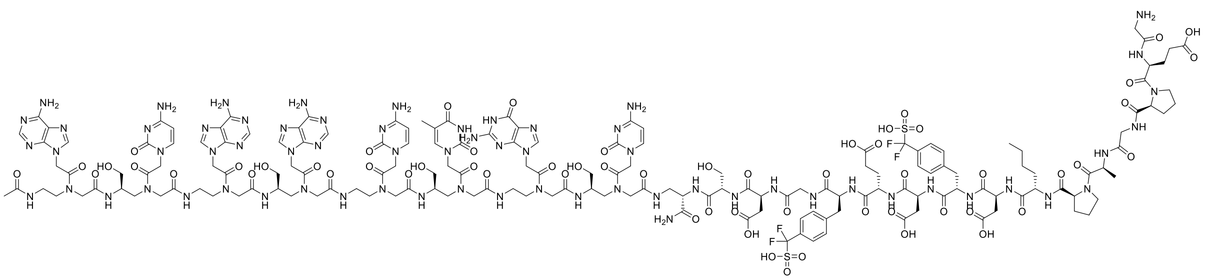

Chemical Formula: $C_{164}H_{215}F_4N_{65}O_{60}S_2$, **Exact Mass**: 4194.52, **Molecular Weight**: 4197.03

MALDI-TOF; m/z found: 4197.05.

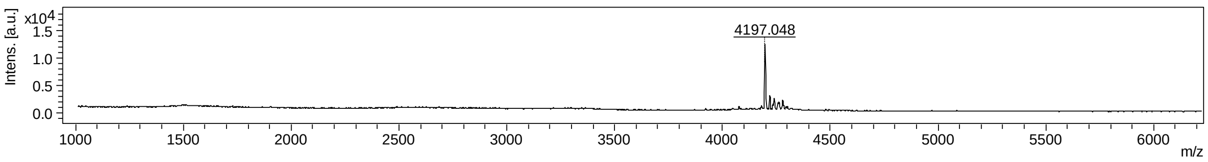

LCMS (ESI); RT= 1.44 min, $[M+3H]^{3+}$: 1399.75, $[M+4H]^{4+}$: 1049.75.

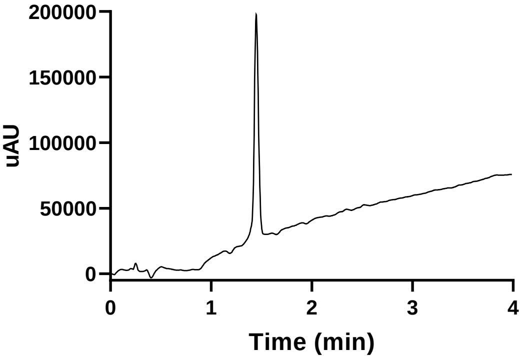

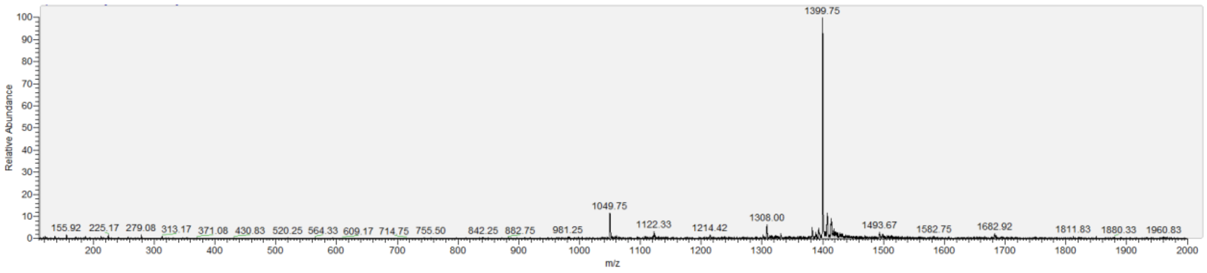

i) Ala scan

E17

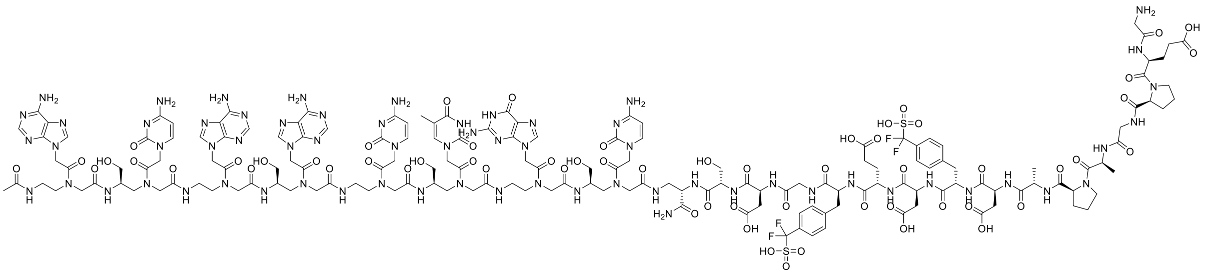

Chemical Formula: $C_{161}H_{209}F_4N_{65}O_{60}S_2$, **Exact Mass**: 4152.47, **Molecular Weight**: 4154.95

MALDI-TOF; m/z found: 4154.51

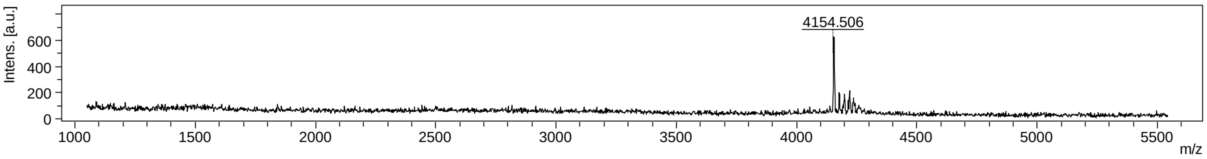

LCMS (ESI); RT= 1.32 min, $[M+3H]^{3+}$: 1385.67, $[M+4H]^{4+}$: 1039.33.

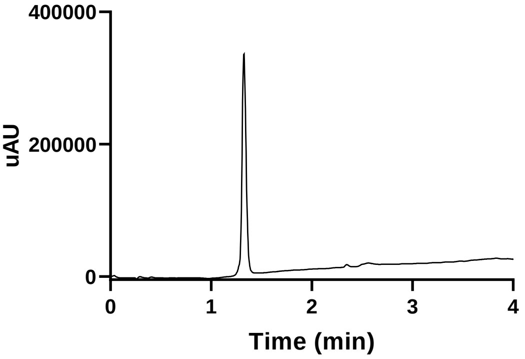

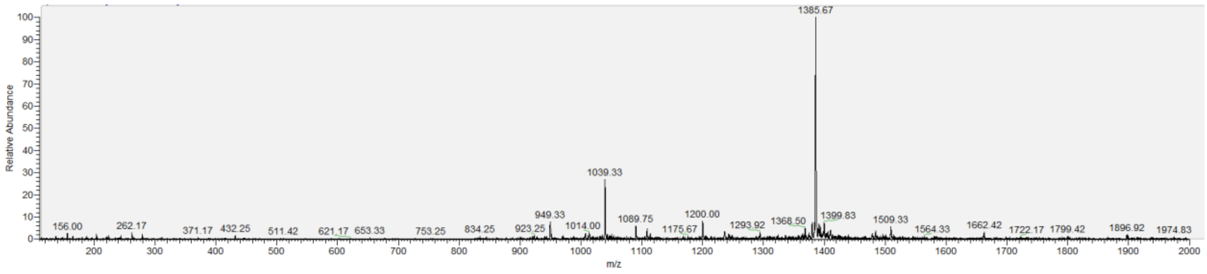

E18

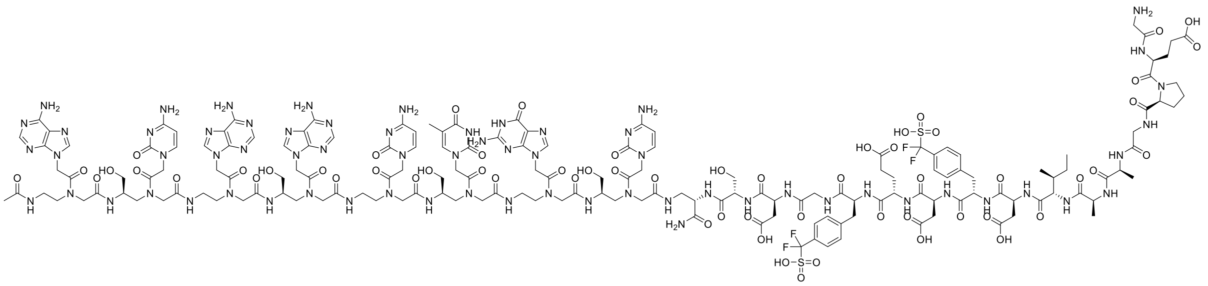

Chemical Formula: $C_{162}H_{213}F_4N_{65}O_{60}S_2$, **Exact Mass**: 4168.50, **Molecular Weight**: 4171.00

MALDI-TOF; m/z found: 4170.71

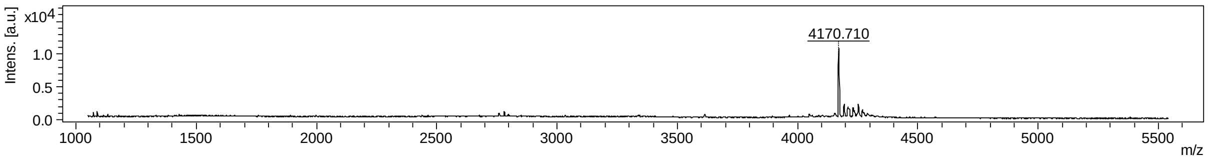

LCMS (ESI); RT= 1.38 min, $[M+3H]^{3+}$: 1391.08, $[M+4H]^{4+}$: 1043.75.

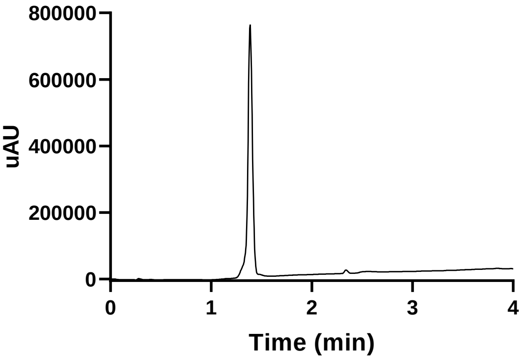

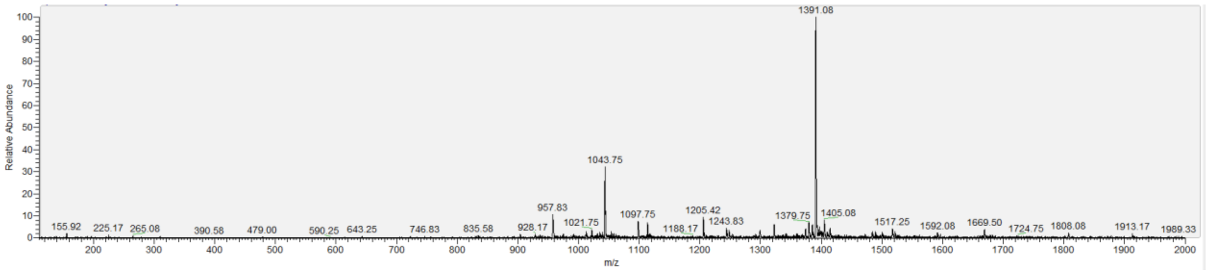

E19

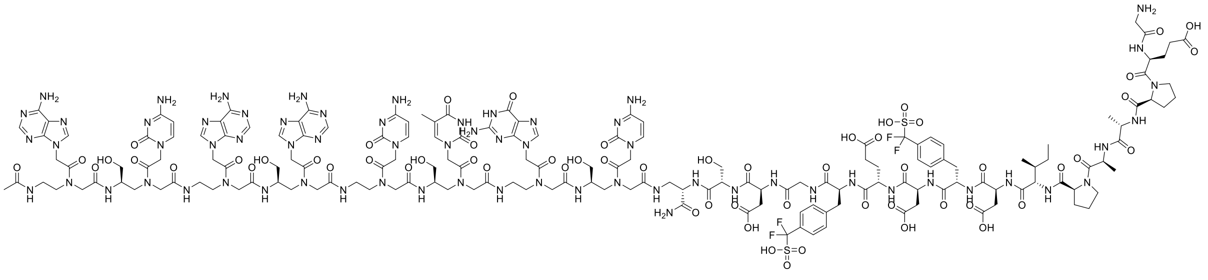

Chemical Formula: $C_{165}H_{217}F_4N_{65}O_{60}S_2$, **Exact Mass**: 4208.53, **Molecular Weight**: 4211.06

MALDI-TOF; m/z found: 4211.61.

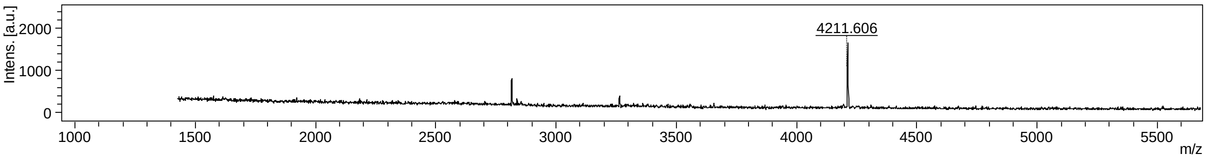

LCMS (ESI); RT= 1.42 min, $[M+3H]^{3+}$: 1404.17, $[M+4H]^{4+}$: 1053.58.

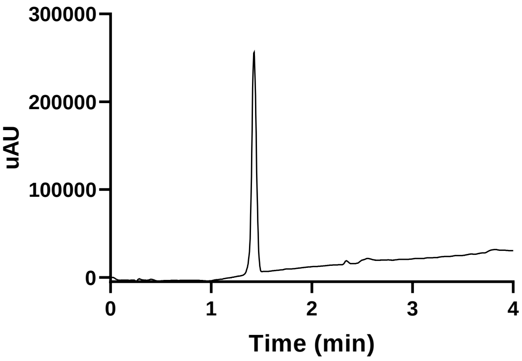

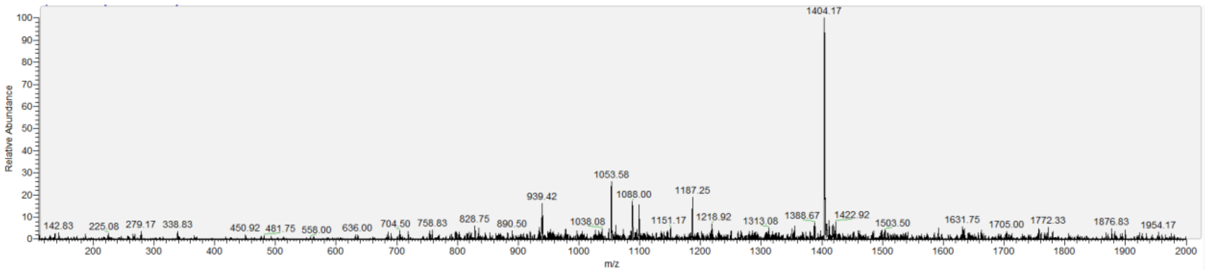

E20

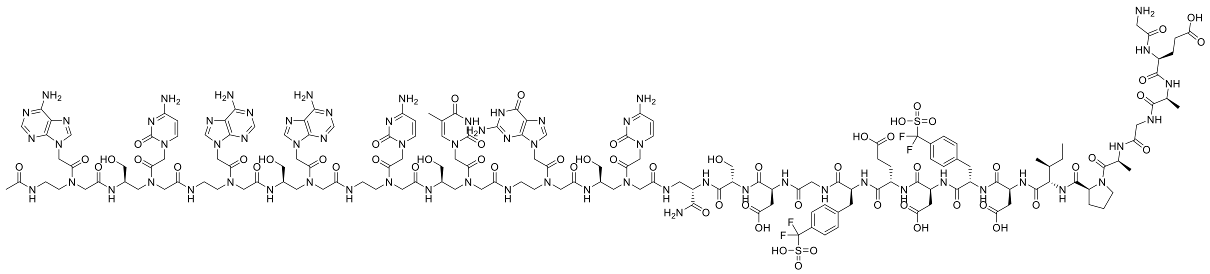

Chemical Formula: $C_{162}H_{213}F_4N_{65}O_{60}S_2$, **Exact Mass**: 4168.50, Molecular Weight: 4171.00

MALDI-TOF; m/z found: 4171.11.

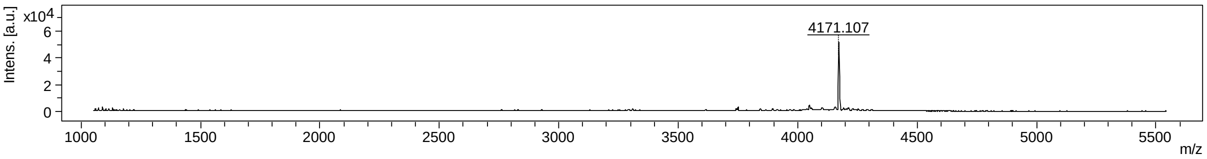

LCMS (ESI); RT= 1.39 min, $[M+3H]^{3+}$: 1391.25, $[M+4H]^{4+}$: 1043.50.

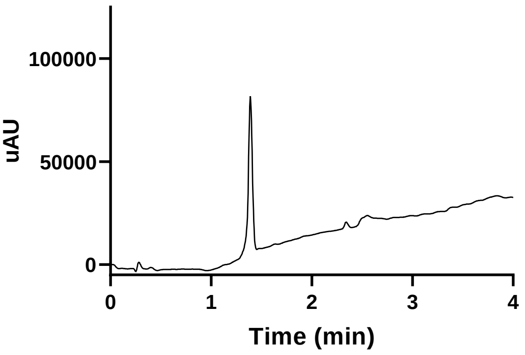

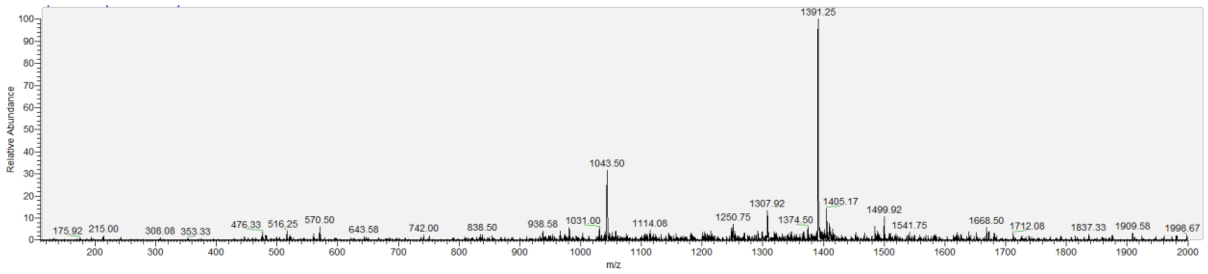

E21

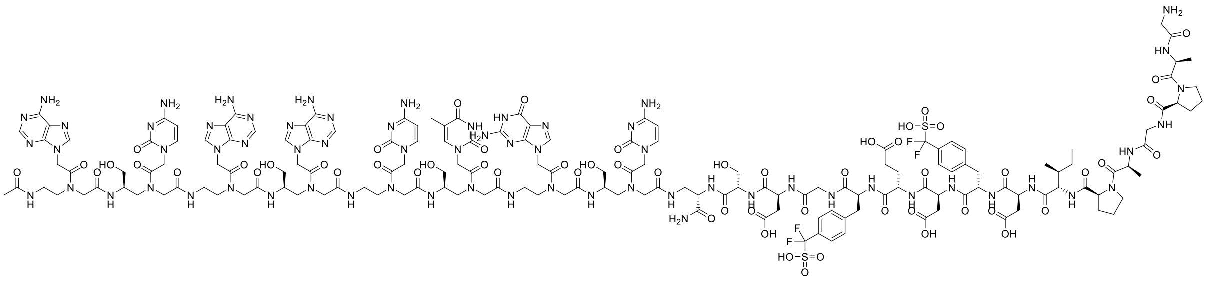

Chemical Formula: $C_{162}H_{213}F_4N_{65}O_{58}S_2$, **Exact Mass**: 4136.51, **Molecular Weight**: 4139.00.

MALDI-TOF; m/z found: 4139.19.

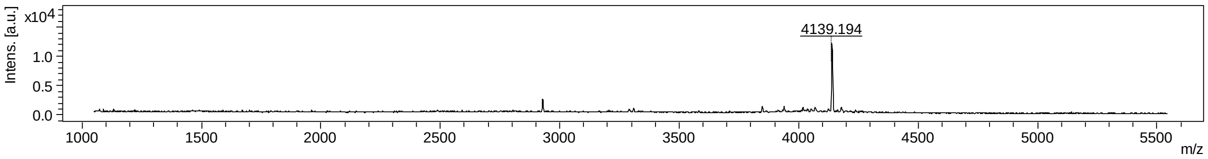

LCMS (ESI); RT= 1.39 min, $[M+3H]^{3+}$: 1380.42, $[M+4H]^{4+}$: 1035.67.

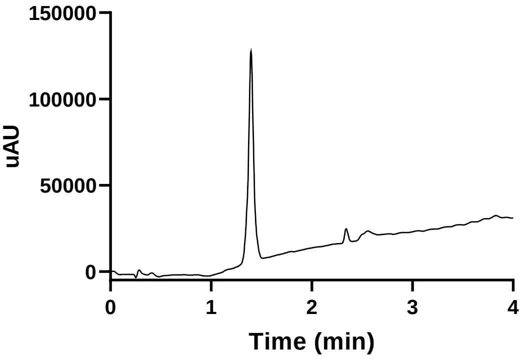

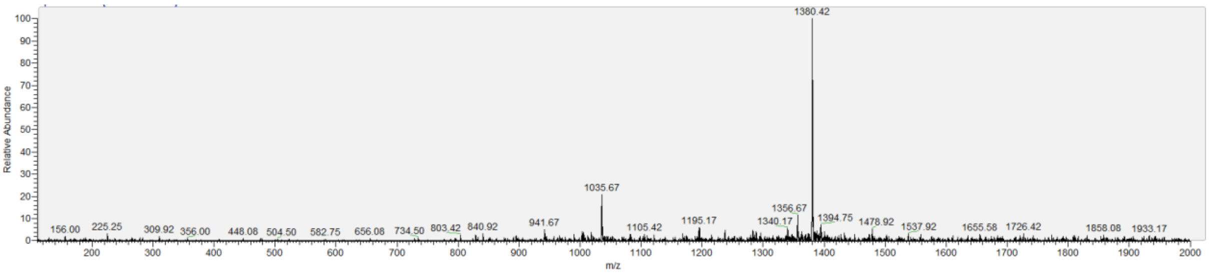

E22

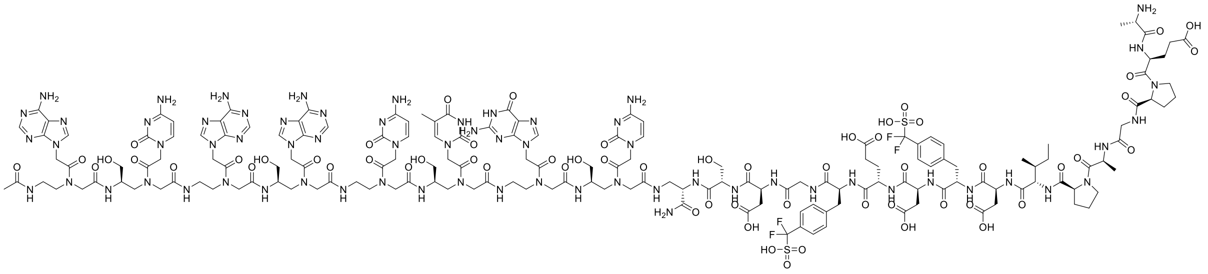

Chemical Formula: $C_{165}H_{217}F_4N_{65}O_{60}S_2$, **Exact Mass**: 4208.53, **Molecular Weight**: 4211.06.

MALDI-TOF; m/z found: 4211.44.

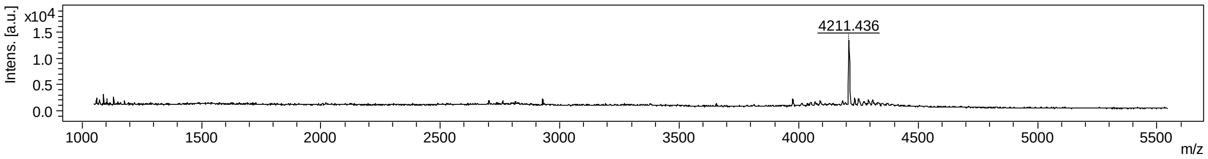

LCMS (ESI); RT= 1.41 min, $[M+3H]^{3+}$: 1404.42, $[M+4H]^{4+}$: 1053.58.

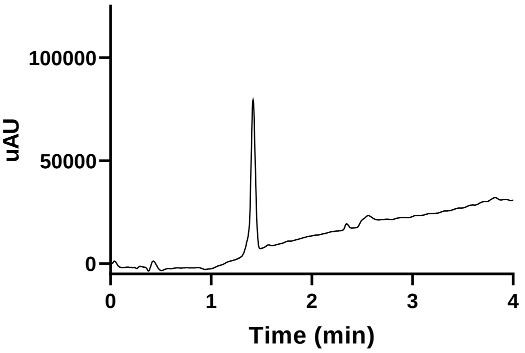

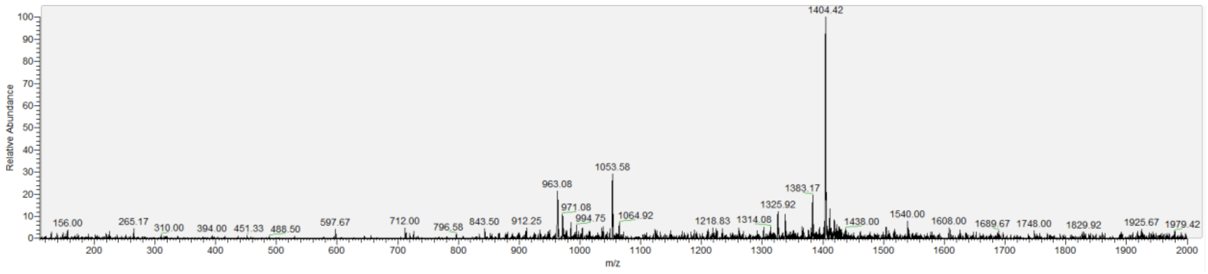

ii) Exosite from different species

E23

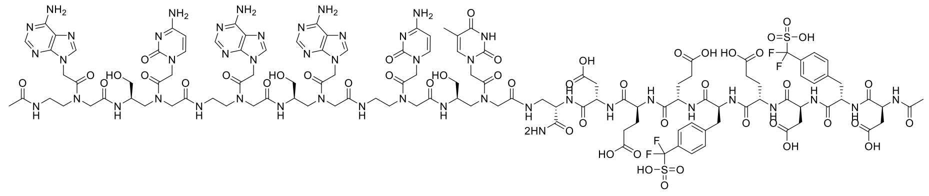

Chemical Formula: $C_{121}H_{152}F_4N_{46}O_{48}S_2$, **Exact Mass**: 3157.02, **Molecular Weight**: 3158.94.

MALDI-TOF; m/z found: 3159.22.

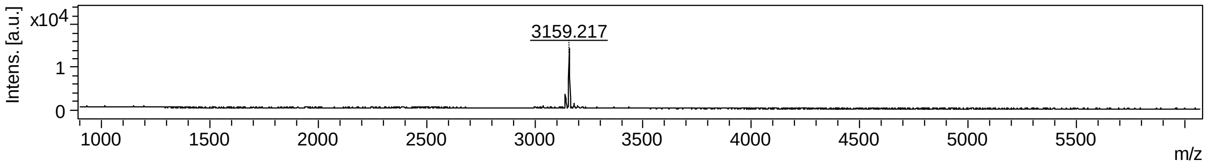

LCMS (ESI); RT= 1.28 min, $[M+3H]^{3+}$: 1053.92.

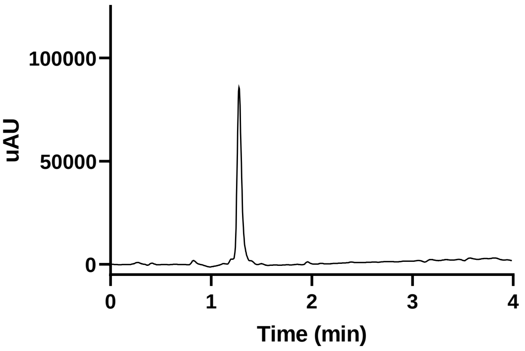

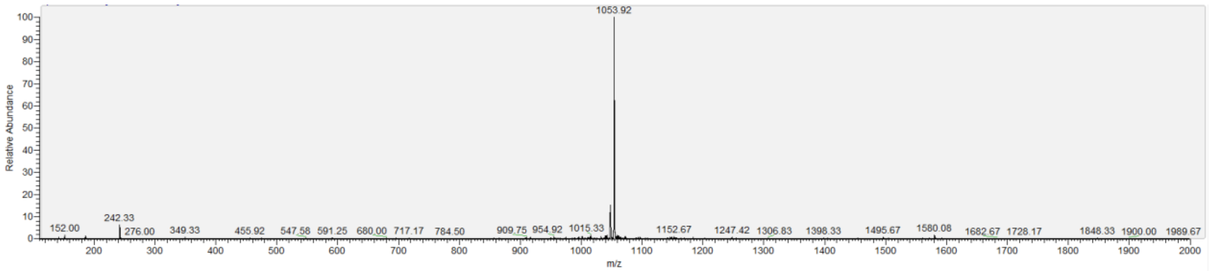

iii) Active Site P3

A4

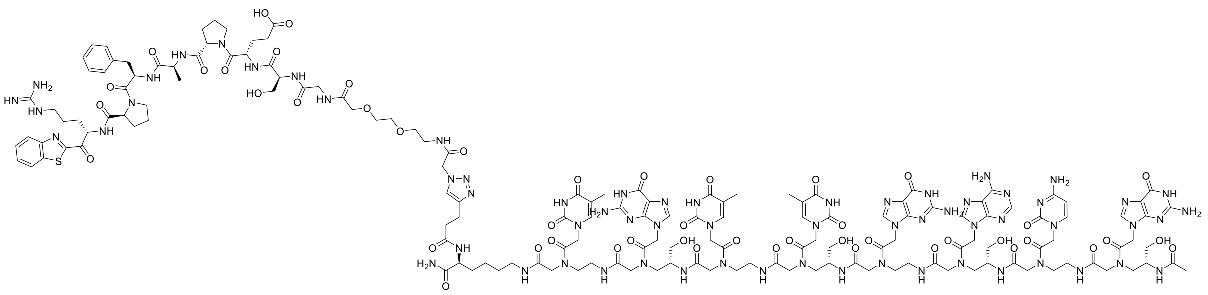

Chemical Formula: $C_{157}H_{208}N_{64}O_{48}S$, **Exact Mass**: 3789.55, **Molecular Weight**: 3791.85.

MALDI-TOF; m/z found: 3791.02.

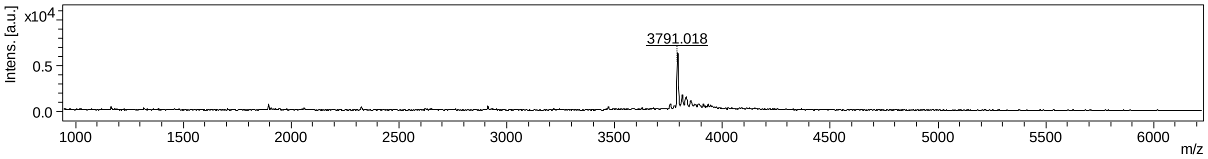

LCMS (ESI); RT= 1.72 min, $[M+3H]^{3+}$: 1264.67, $[M+4H]^{4+}$: 948.92, $[M+5H]^{5+}$: 759.25.

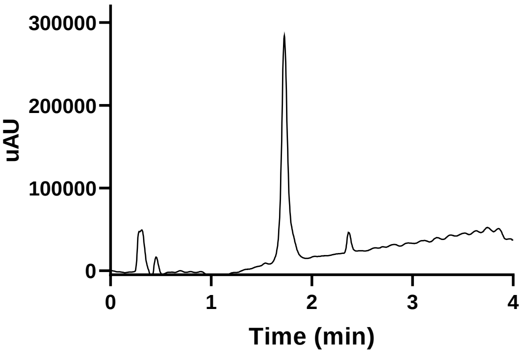

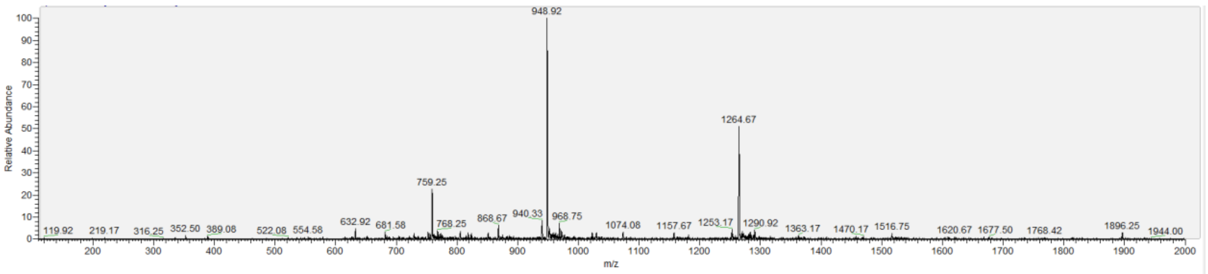

<u>**A5**</u>

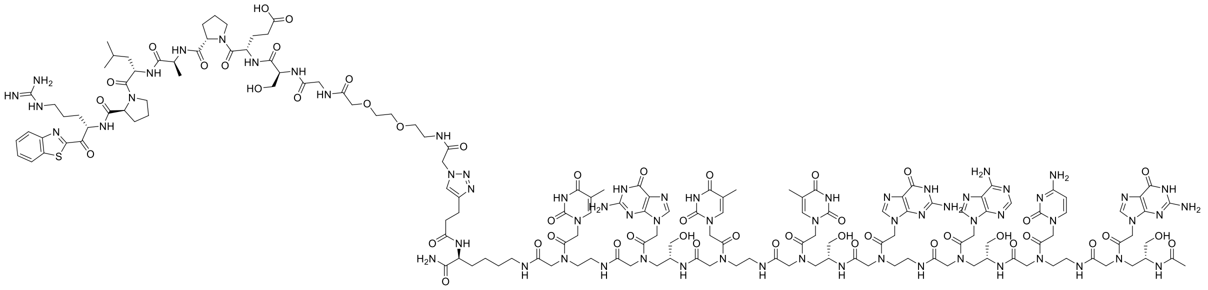

Chemical Formula: $C_{154}H_{210}N_{64}O_{48}S$, **Exact Mass**: 3755.57, **Molecular Weight**: 3757.83.

MALDI-TOF; m/z found: 3757.59.

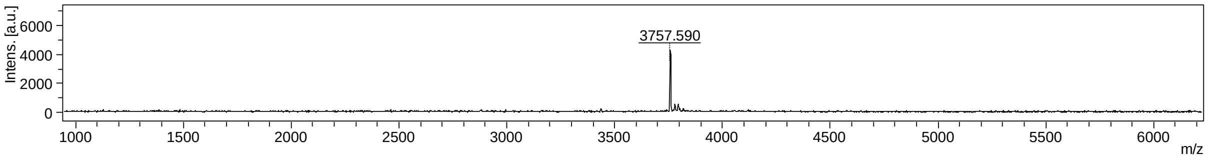

LCMS (ESI); RT= 1.71 min, $[M+3H]^{3+}$: 1253.25, $[M+4H]^{4+}$: 940.33, $[M+5H]^{5+}$: 752.33.

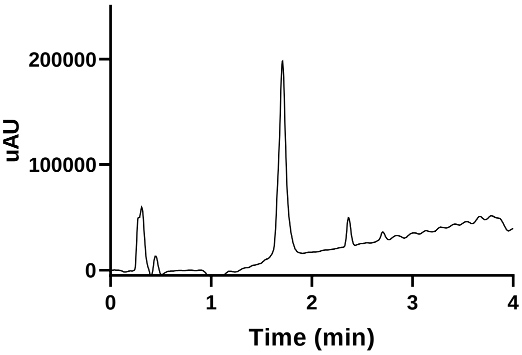

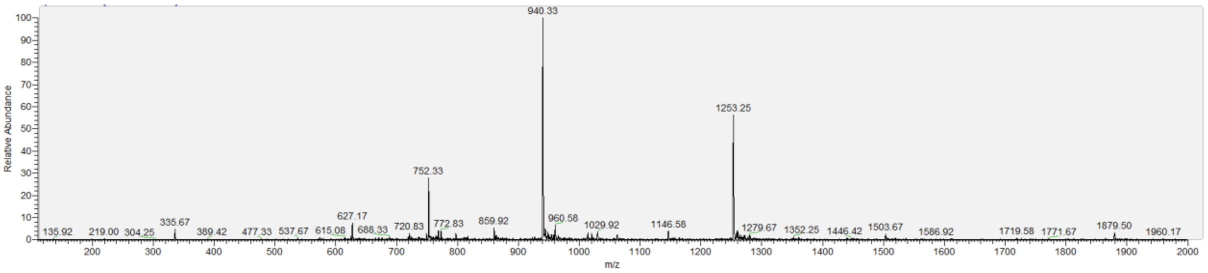

A6

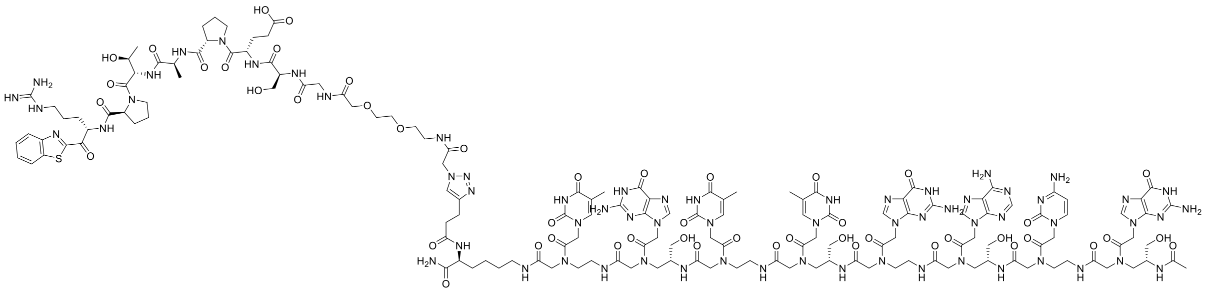

Chemical Formula: $C_{152}H_{206}N_{64}O_{49}S$, **Exact Mass**: 3743.53, **Molecular Weight**: 3745.78.

MALDI-TOF; m/z found: 3745.70.

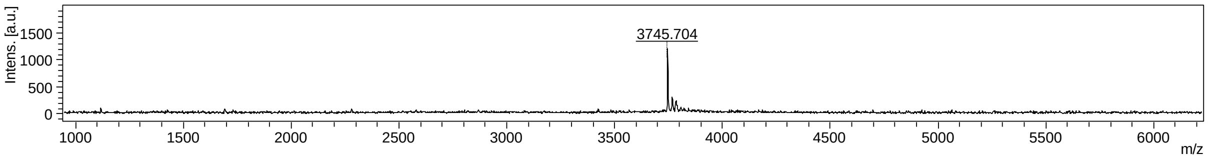

LCMS (ESI); RT= 1.53 min, $[M+3H]^{3+}$: 1249.25, $[M+4H]^{4+}$: 937.25, $[M+5H]^{5+}$: 750.08.

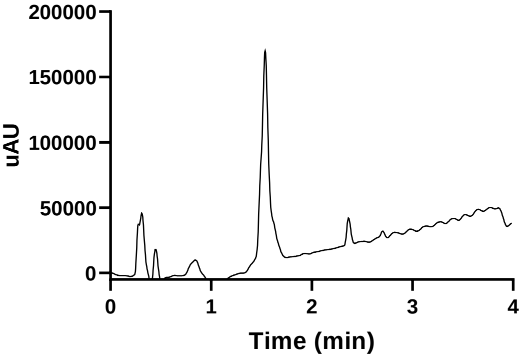

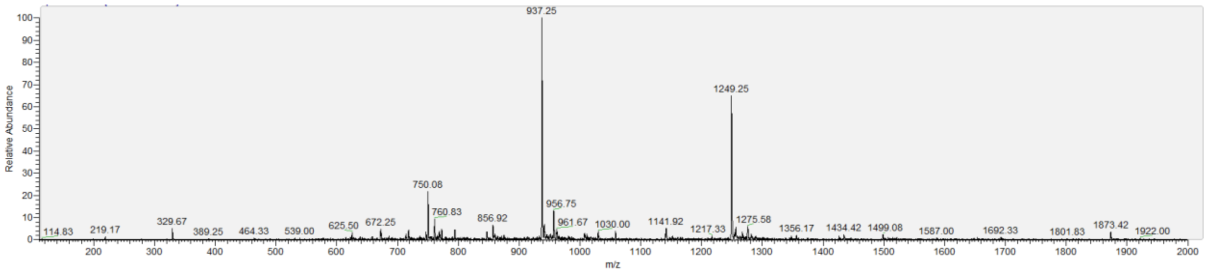

A7

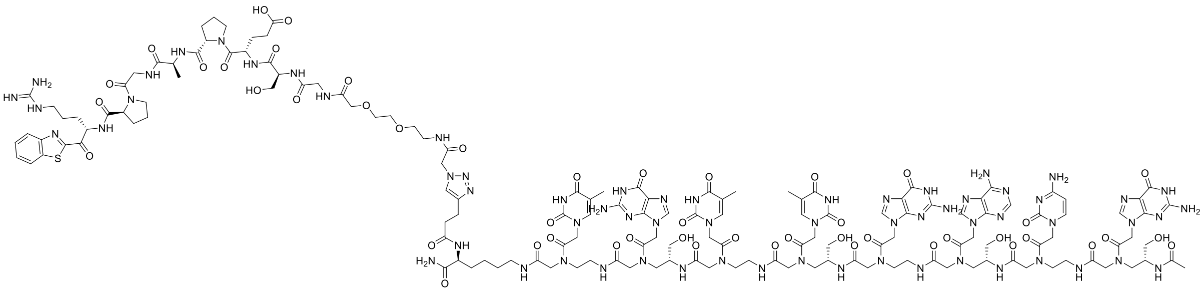

Chemical Formula: $C_{150}H_{202}N_{64}O_{48}S$, **Exact Mass**: 3699.51, **Molecular Weight**: 3701.73.

MALDI-TOF; m/z found: 3701.51.

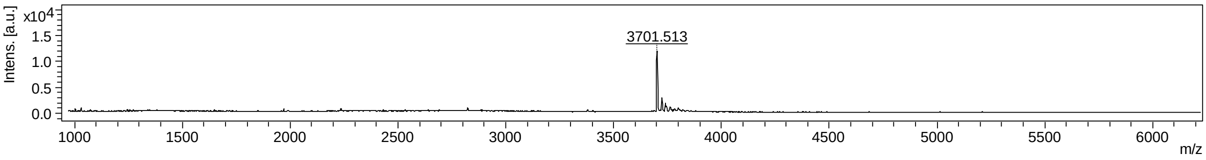

LCMS (ESI); RT= 1.52 min, $[M+3H]^{3+}$: 1234.58, $[M+4H]^{4+}$: 926.33.

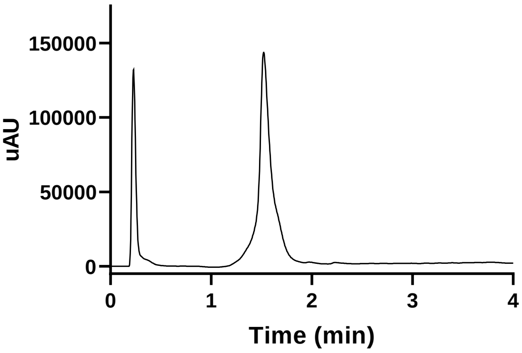

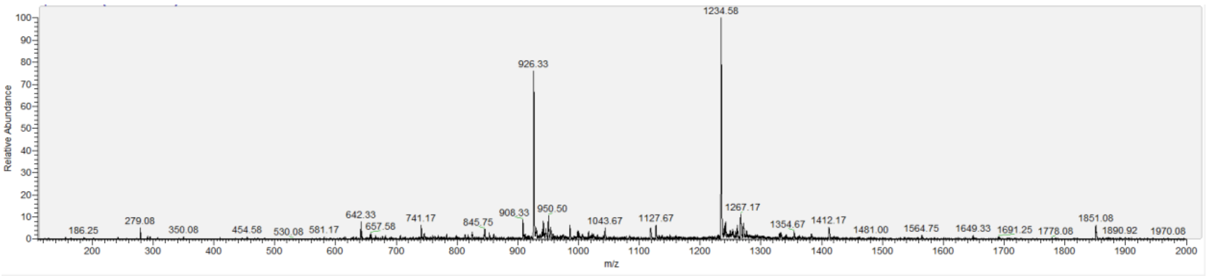

iv) Antidote PNA

Fmoc D monomer was prepared according to the procedure reported by Sugiyama *et al.*[2]

AD1

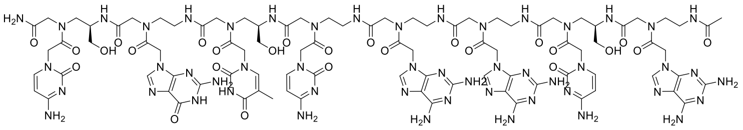

Chemical Formula: $C_{90}H_{119}N_{51}O_{26}$, **Exact Mass**: 2329.96, **Molecular Weight**: 2331.27.

MALDI-TOF; m/z found: 2330.85.

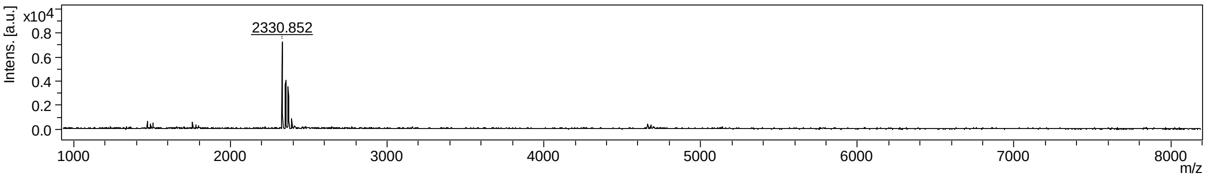

LCMS (ESI); RT= 1.02 min, $[M+2H]^{2+}$: 1166.33, $[M+3H]^{3+}$: 778.00, $[M+4H]^{4+}$: 583.83.

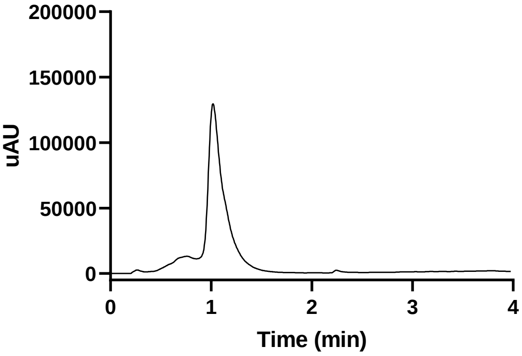

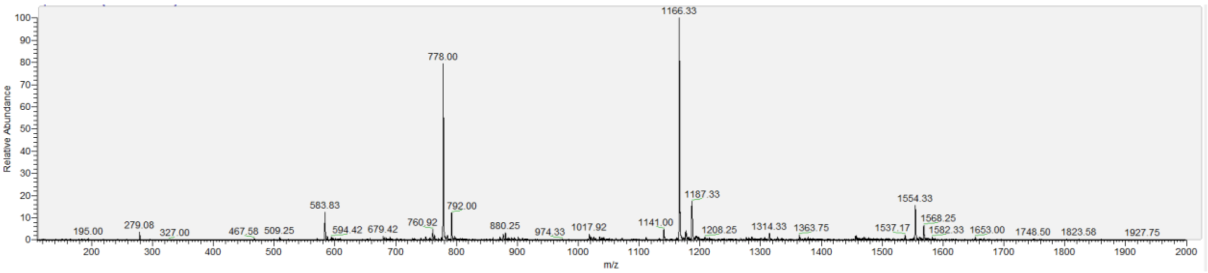

HRMS; $[M+2H]^{2+}$: 1166.4845, $[M+3H]^{3+}$: 777.9944, $[M+4H]^{4+}$: 583.7455.

AD2

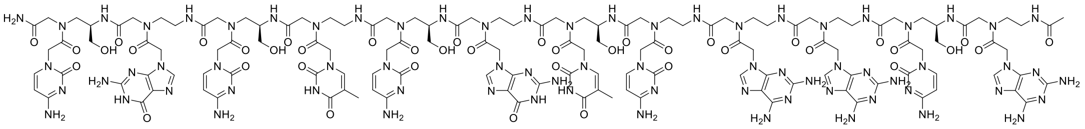

Chemical Formula: $C_{134}H_{176}N_{72}O_{41}$, **Exact Mass**: 3449.36, **Molecular Weight**: 3451.35

MALDI-TOF; m/z found: 3451.04.

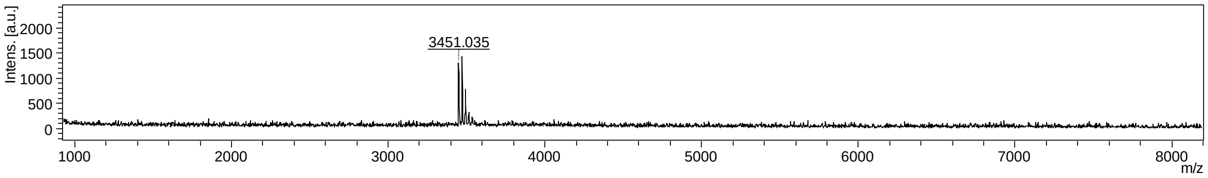

LCMS (ESI); RT= 1.22 min, $[M+2H]^{2+}$: 1725.83, $[M+3H]^{3+}$: 1151.25, $[M+4H]^{4+}$: 863.83.

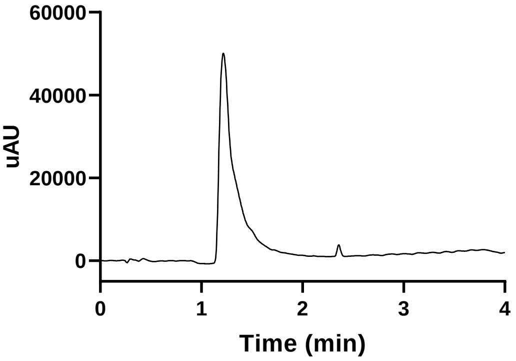

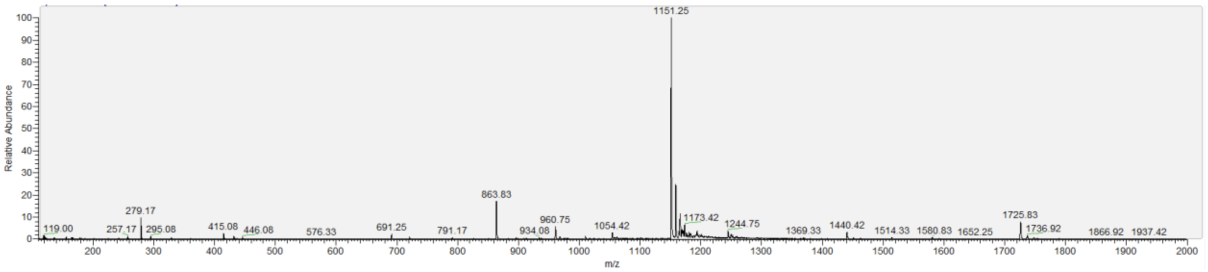

HRMS; $[M+3H]^{3+}$: 1151.1405, $[M+4H]^{4+}$: 863.6079, $[M+5H]^{5+}$: 691.0870.

v) PNA for SPR measurements

<u>Biotin-PNA(8mer)</u>

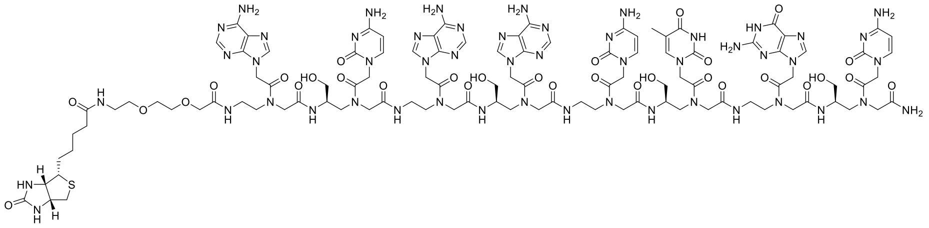

Chemical Formula: $C_{105}H_{141}N_{51}O_{31}S$, **Exact Mass**: 2644.08, **Molecular Weight**: 2645.67.

MALDI-TOF; m/z found: 2645.35.

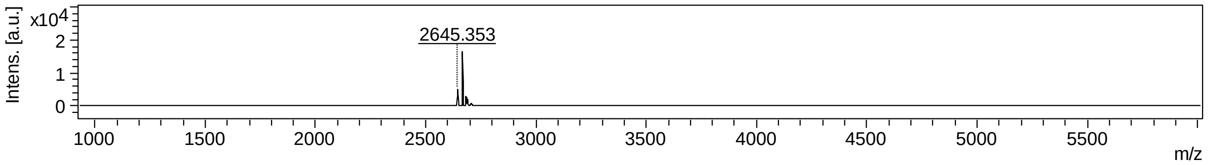

LCMS (ESI); RT= 1.26 min, $[M+2H]^{2+}$: 1323.25, $[M+3H]^{3+}$: 882.67, $[M+4H]^{4+}$: 662.25.

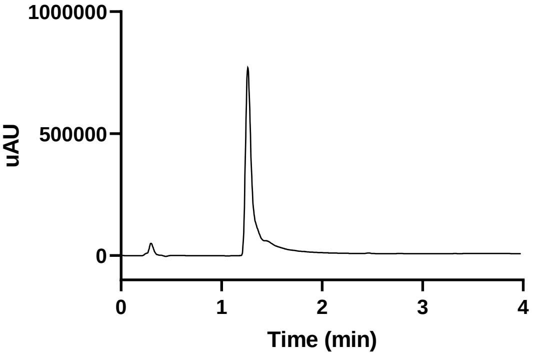

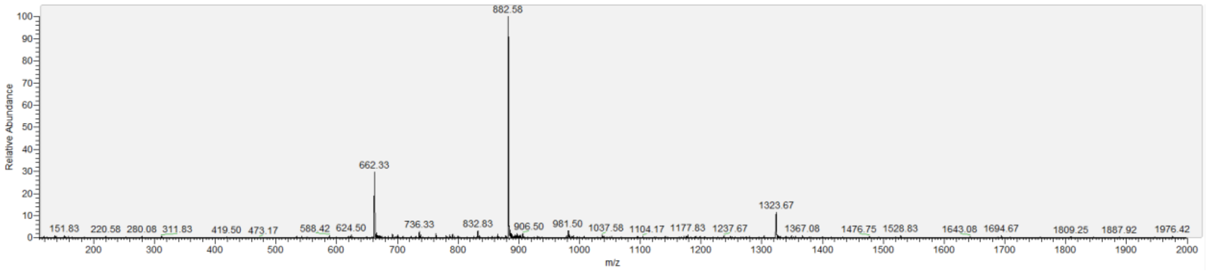

PNA(8mer)

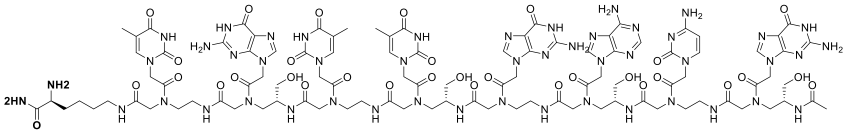

Chemical Formula: $C_{99}H_{132}N_{48}O_{32}$, **Exact Mass**: 2505.02, **Molecular Weight**: 2506.45.

MALDI-TOF; m/z found: 2506.26.

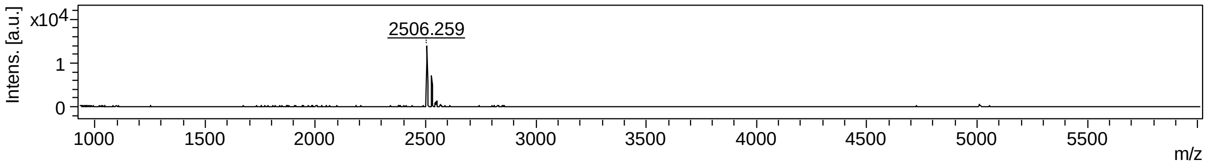

LCMS (ESI); RT= 1.15 min, $[M+2H]^{2+}$: 1254.33, $[M+3H]^{3+}$: 836.25, $[M+4H]^{4+}$: 627.25.

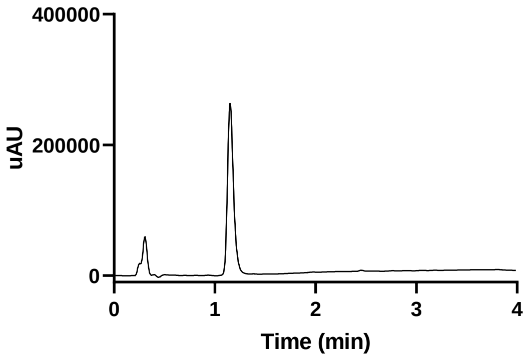

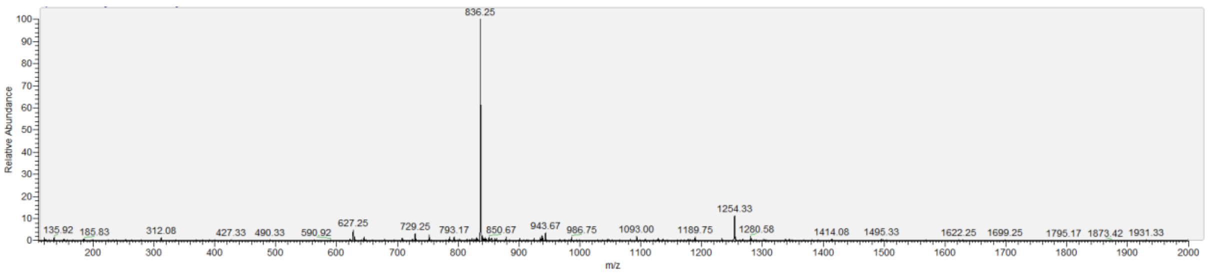

PNA(6mer)

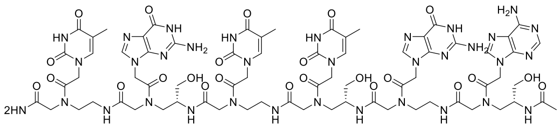

Chemical Formula: $C_{71}H_{92}N_{34}O_{24}$, **Exact Mass**: 1804.70, **Molecular Weight**: 1805.73.

MALDI-TOF; m/z found: 1805.09.

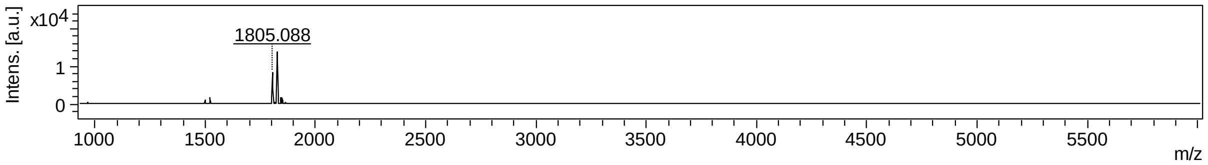

LCMS (ESI); RT= 1.14 min, $[M+2H]^{2+}$: 903.42, $[M+3H]^{3+}$: 602.58.

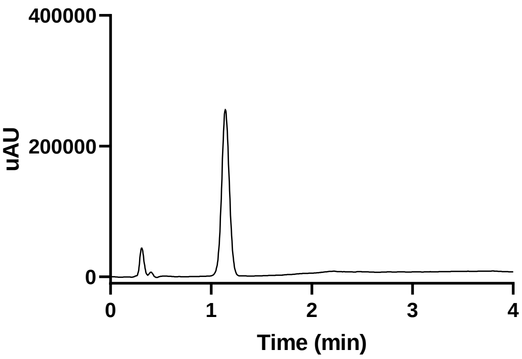

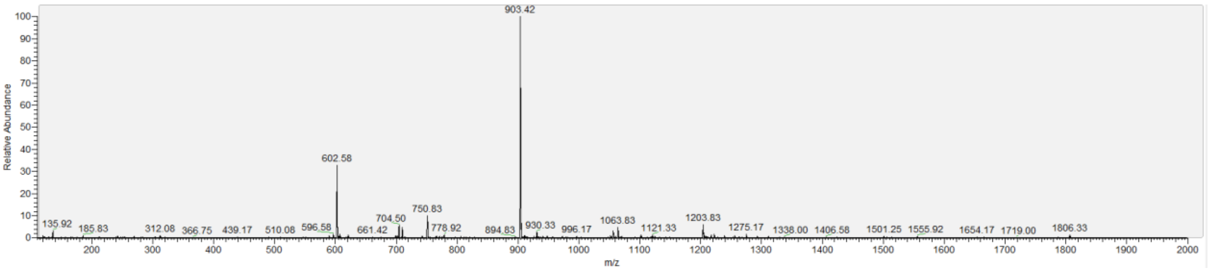

PNA(4mer)

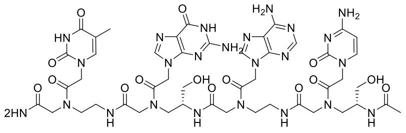

Chemical Formula: $C_{47}H_{62}N_{24}O_{15}$, **Exact Mass**: 1202.48, **Molecular Weight**: 1203.17.

MALDI-TOF; m/z found: 1202.95.

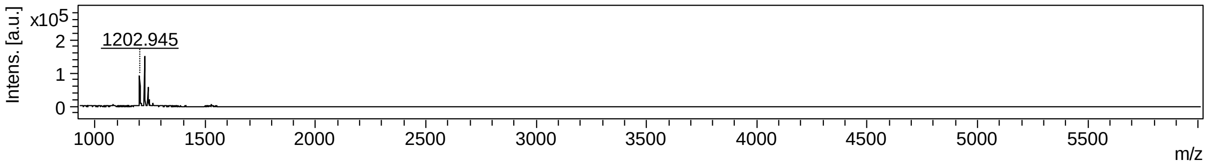

LCMS (ESI); RT= 0.46 min, $[M+H]^{1+}$: 1203.25, $[M+3H]^{3+}$: 602.25.

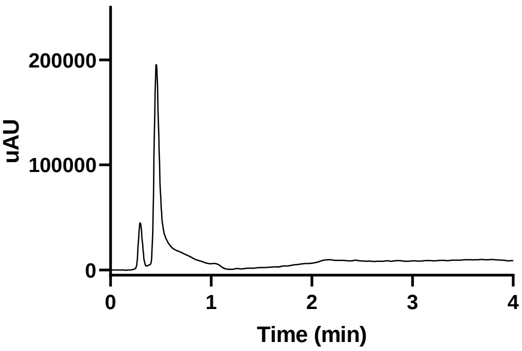

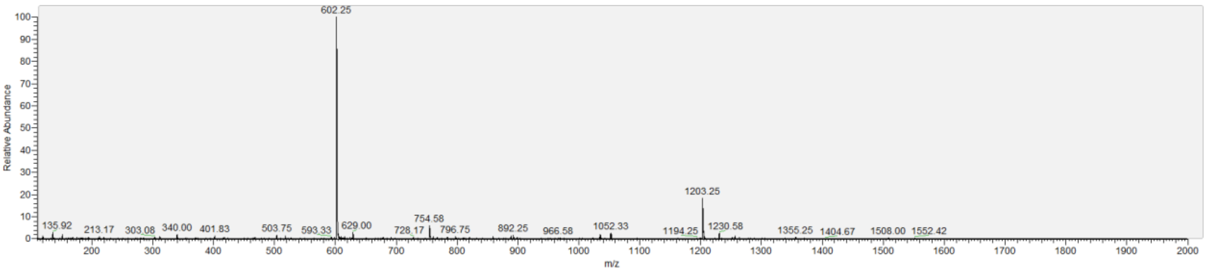

5. Thrombin Inhibition Assay

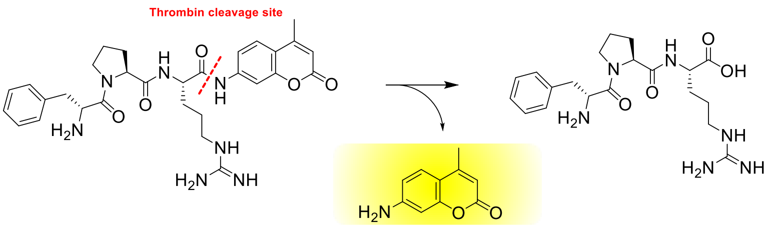

Supplementary Figure 2: Thrombin Fluorogenic Assay

The assay was performed as described in the Online Methods section.

To convert IC50 values to Ki values, K_M was determined: The assay described above was performed without inhibitor but with variation of [substrate]. The Michaelis Menten plot was obtained, and K_M and Vmax were determined to be 2.416 µM and 435.8 respectively (R^2: 0.98).

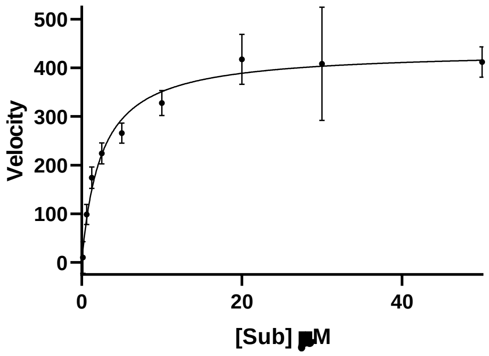

Supplementary Figure 3: Michaelis Menten Plot

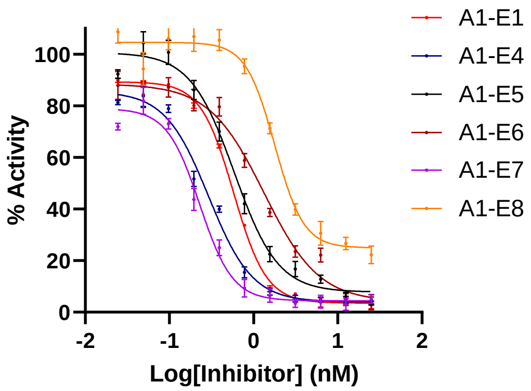

Supplementary Figure 4: IC50 curves for compounds displayed in Figure 2e

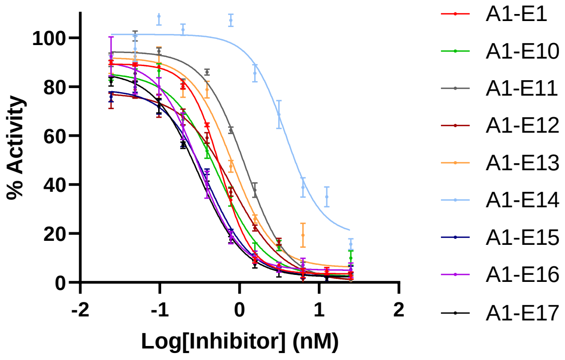

Supplementary Figure 5: IC50 curves for compounds displayed in Figure 2f

The recovery of activity (%) stated in the main text were calculated from the linear regressions of the slope (shown in red on the graph below) at the time stated (30 minutes or 90 minutes after antidote addition) compared to the linear regression of the initial activity of thrombin in the absence of inhibitor.

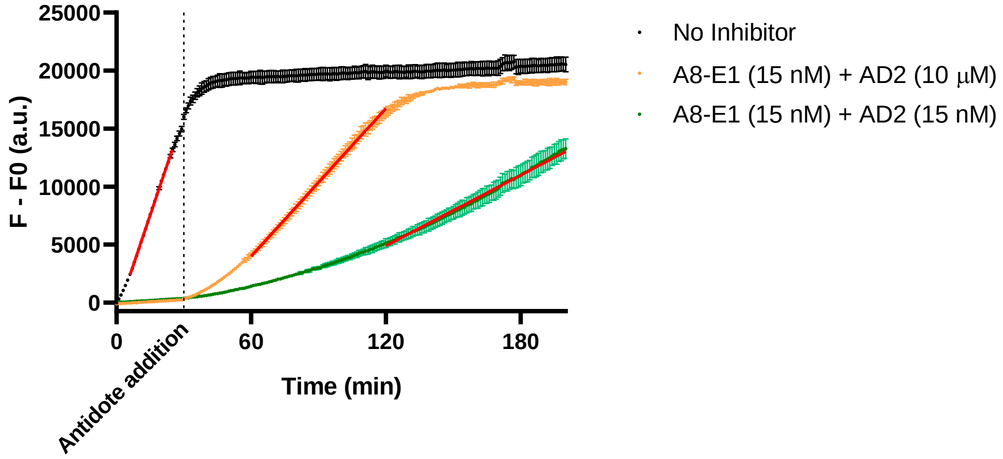

Supplementary Figure 6: Recovery of activity upon antidote addition

Supplementary Table1: Linear regression parameters for recovery of activity upon antidote addition

	No Inhibitor	A8-E1+AD2 10 μM	A8-E1+AD2 15 nM
**Best-fit values**			
Slope	573.4	212.5	101.4
Y-intercept	-1064	-8763	-7271
X-intercept	1.856	41.23	71.69
**Std. Error**			
Slope	2.098	1.330	0.9244
Y-intercept	34.69	122.0	171.5
**Goodness of Fit**			
R square	0.9992	0.9930	0.9798
Equation	Y = 573.4*X - 1064	Y = 212.5*X - 8763	Y = 101.4*X - 7271

6. Needle Injury Thrombosis Model – Max Intensity Projections

The needle injury thrombosis model was performed as described in the Online Methods section. The max intensity projections for the *in vivo* data shown in Fig.3 and Fig.4 are displayed in the table below.

Supplementary Table 2: Max intensity projections for the *in vivo* data shown in Fig.3 and Fig.4

No inhibitor	Argatroban	A1-E1	A8-E1	A8-E1 + AD2
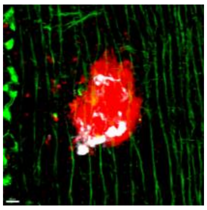	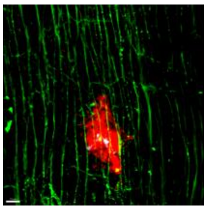	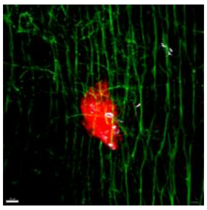	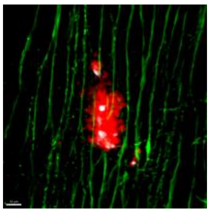	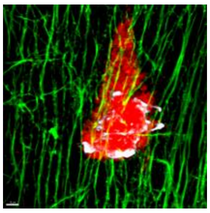

7. References

(1) Jakobsche, C. E.; McEnaney, P. J.; Zhang, A. X.; Spiegel, D. A. Reprogramming Urokinase into an Antibody-Recruiting Anticancer Agent. *ACS Chemical Biology* **2012**, *7* (2), 316-321. DOI: 10.1021/cb200374e.
(2) Sugiyama, T.; Hasegawa, G.; Niikura, C.; Kuwata, K.; Imamura, Y.; Demizu, Y.; Kurihara, M.; Kittaka, A. PNA monomers fully compatible with standard Fmoc-based solid-phase synthesis of pseudocomplementary PNA. *Bioorganic & Medicinal Chemistry Letters* **2017**, *27* (15), 3337-3341. DOI: https://doi.org/10.1016/j.bmcl.2017.06.015.
