# Supplementary material for: Development of supramolecular anticoagulants with on-demand reversibility
Source: Nat Biotechnol. 2024 Apr 30;43(2):186–93. doi: 10.1038/s41587-024-02209-z (PMC11825364; doi:10.1038/s41587-024-02209-z)
Supplement: Supplementary file 2 — Reporting Summary [file 41587_2024_2209_MOESM2_ESM.pdf]

Corresponding author(s): Nicolas Winssinger

Last updated by author(s): Feb 28, 2024

Reporting Summary

Nature Portfolio wishes to improve the reproducibility of the work that we publish. This form provides structure for consistency and transparency in reporting. For further information on Nature Portfolio policies, see our Editorial Policies and the Editorial Policy Checklist.

Statistics

For all statistical analyses, confirm that the following items are present in the figure legend, table legend, main text, or Methods section.

n/a	Confirmed	
☐	☒	The exact sample size (*n*) for each experimental group/condition, given as a discrete number and unit of measurement
☐	☒	A statement on whether measurements were taken from distinct samples or whether the same sample was measured repeatedly
☐	☒	The statistical test(s) used AND whether they are one- or two-sided<br>*Only common tests should be described solely by name; describe more complex techniques in the Methods section.*
☒	☐	A description of all covariates tested
☒	☐	A description of any assumptions or corrections, such as tests of normality and adjustment for multiple comparisons
☐	☒	A full description of the statistical parameters including central tendency (e.g. means) or other basic estimates (e.g. regression coefficient) AND variation (e.g. standard deviation) or associated estimates of uncertainty (e.g. confidence intervals)
☐	☒	For null hypothesis testing, the test statistic (e.g. *F*, *t*, *r*) with confidence intervals, effect sizes, degrees of freedom and *P* value noted<br>*Give P values as exact values whenever suitable.*
☒	☐	For Bayesian analysis, information on the choice of priors and Markov chain Monte Carlo settings
☒	☐	For hierarchical and complex designs, identification of the appropriate level for tests and full reporting of outcomes
☒	☐	Estimates of effect sizes (e.g. Cohen's *d*, Pearson's *r*), indicating how they were calculated

Our web collection on statistics for biologists contains articles on many of the points above.

Software and code

Policy information about availability of computer code

Data collection	LCMS was acquired using Thermo Xcalibur 2.2.SP1.48, NMR using Bruker IconNMR 5.0.10.Build19 and MALDI using Flex control 3.4<br>The thrombin fluorogenic assay and fibrinogen assay data was collected on a SpectraMax i3x.<br>The SPR data was acquired on a biacore T200 machine.<br>The selectivity assays were run on a Synergy2 plate reader.<br>The ex-vivo aPTT assay was run on a ClarioStar plate reader.<br>CAT experiments were performed via a Hemker Calibrated Automated Thrombinoscope (Diagnostica Stago) using a Fluoroskan Ascent® plate reader.<br>The in-vivo needle injury model was monitored using a confocal intravital microscopy platform (Nikon A1R-si; objective: Apo LWD, ×40 magnification, 1.15 numerical aperture, water immersion; sequential excitation: 488-, 561-, and 638-nm lasers; emission: 525/50-, 595/50-, and 700/75-nm filters; using NIS Elements Advanced Research acquisition software, version 5.02.
Data analysis	LCMS was analysed using Thermo Xcalibur Qual Browser 2.2.SP1.48, NMR using MestReNova v 12.0.1-20560 and MALDI using Flex analysis 3.4<br>The thrombin fluorogenic assay and fibrinogen assay data analysed using GraphPad 8.4.3.<br>The SPR data was analysed using the Biacore T200 analyis software.<br>The selectivity assays were analysed using GraphPad 10.2.0.<br>The ex-vivo aPTT assay were analysed using GraphPad 10.2.0.<br>CAT experiments were analysed using GraphPad 10.2.0.<br>The quantifications of the in-vivo needle injury model were analysed using GraphPad 10.2.0.

For manuscripts utilizing custom algorithms or software that are central to the research but not yet described in published literature, software must be made available to editors and reviewers. We strongly encourage code deposition in a community repository (e.g. GitHub). See the Nature Portfolio guidelines for submitting code & software for further information.

Data

Policy information about availability of data

All manuscripts must include a data availability statement. This statement should provide the following information, where applicable:

- Accession codes, unique identifiers, or web links for publicly available datasets
- A description of any restrictions on data availability
- For clinical datasets or third party data, please ensure that the statement adheres to our policy

The data supporting the findings of this study are available within this paper and its Supplementary Information. All the raw data has been deposited on Zenodo (https://doi.org/10.5281/zenodo.10473739). Source data are provided with this paper.
Previously published PDBs that are mentioned and shown in the main text can be found online with the following codes: tick-derived madanin-1 (PDB 5L6N) and TTI from the tsetse fly (PDB 6TKG).

Human research participants

Policy information about studies involving human research participants and Sex and Gender in Research.

Reporting on sex and gender	N/A
Population characteristics	N/A
Recruitment	N/A
Ethics oversight	N/A

Note that full information on the approval of the study protocol must also be provided in the manuscript.

Field-specific reporting

Please select the one below that is the best fit for your research. If you are not sure, read the appropriate sections before making your selection.

☒ Life sciences ☐ Behavioural & social sciences ☐ Ecological, evolutionary & environmental sciences

For a reference copy of the document with all sections, see nature.com/documents/nr-reporting-summary-flat.pdf

Life sciences study design

All studies must disclose on these points even when the disclosure is negative.

Sample size

For intravital animal studies, we have estimated the group size based on power calculations (These cohort sizes were calculated using GraphPad StatMate 2.00 and online calculator; with 80% power and significance level of 0.05), as well as experience from previous studies. Each animal received a minimum of 2 and maximum of 4 injuries per animal over multiple vessels.
It should be clear from the graph but if required, below is the breakdown of our cohorts where n is our sample size and N is representative of the total number of injuries over the cohort:
a. Control injury n=7, N=12
b. A8-E1 n=3, N=11
c. A8-E1+AD2 n=3, N=15
d. Argatroban n=4, N=9

Data exclusions

No data was excluded unless technical failure was encountered.

Replication

For the thrombin fluorogenic assay and fibrinogen assay each concentration was measured in triplicate - all replicates were used for analysis. In almost all cases the assay was only performed once unless technical failure was encountered.
For the SPR experiments, each concentration was measured in triplicate - all replicates were used for analysis. The experiment was performed once.
For the protease selectivity panel, each concentration was measured in duplicate. No data was excluded or repeated.
CAT experiments were performed in triplicate. No data was excluded or repeated.
The in-vivo mice experiments were replicated on a minimum of 3 mice. Statistical significance between multiple treatment groups was analyzed using a one-way analysis of variance (ANOVA) with Tukey's post-testing with a single pooled variance. (Prism software ver. 10.2; GraphPAD Software for Science, San Diego, CA). Data are presented as means ± SEM where 'n' equals the number of independent experiments performed.

Randomization

Covariates were not applicable as control injuries were routinely performed in each mouse prior to administration of drug, and therefore served as an individual internal controls for each experimental animal.

Blinding

In Argatroban cohorts, drug was administered via a bolus followed by and infusion using a jugular catheter and infusion pump while A1-E1

Blinding | was administered via the delivery of multiple intravenous boluses. The distinct delivery mechanisms of these drugs made true blinding of these cohorts impractical.

Reporting for specific materials, systems and methods

We require information from authors about some types of materials, experimental systems and methods used in many studies. Here, indicate whether each material, system or method listed is relevant to your study. If you are not sure if a list item applies to your research, read the appropriate section before selecting a response.

Materials & experimental systems

n/a	Involved in the study
☐	☒ Antibodies
☒	☐ Eukaryotic cell lines
☒	☐ Palaeontology and archaeology
☐	☒ Animals and other organisms
☒	☐ Clinical data
☒	☐ Dual use research of concern

Methods

n/a	Involved in the study
☒	☐ ChIP-seq
☒	☐ Flow cytometry
☒	☐ MRI-based neuroimaging

Antibodies

Antibodies used

Antibodies used:
a. Anti-fibrin (59D8) mouse monoclonal antibody (0.35mg/ml); Merck; MABS2155
b. DyLight 649-labeled Anti-Mouse GPIbα (CD42b) rat monoclonal antibody; Emfret Analytics; X649 (0.1mg/ml) #Lot 649-D

Validation

5. DyLight 649-labeled Anti-Mouse GPIbα (CD42b) rat monoclonal antibody was purchased from commercial sources and the hybridoma secreting the fibrin-specific mAb 59D8 was generated as described previously (Kwan Y. Hui et al., Monoclonal Antibodies to a Synthetic Fibrin-Like Peptide Bind to Human Fibrin But Not Fibrinogen. Science, 222,1129-1132 (1983)). Antibodies were used and stored as recommended by the manufacturer. We performed further validation of antibodies by cross-checking and evaluating the literature and other users. We also performed appropriate controls to validate the antibodies.

Animals and other research organisms

Policy information about studies involving animals; ARRIVE guidelines recommended for reporting animal research, and Sex and Gender in Research

Laboratory animals

All experiments and procedures performed on mice were approved by the University of Sydney Animal Ethics Committee (USYD AEC, Protocol 2021/1912, title: "Investigate potent and safe anti-clotting therapies for ischaemia reperfusion diseases"). All procedures were performed in accordance with the guidelines of the National Health and Medical Research Council Code of Practice for the Care and the Use of Animals for Experimental Purpose in Australia. C57BL/6 male mice were purchased from Australian BioResources Ltd (ABR), NSW, maintained at the Charles Perkins Centre/Laboratory Animal Services under specific pathogen-free (SPF) conditions at 21°C, 45-55% relative humidity, and a 12-hour light-dark cycle with ad libitum access to food and water, and used at the age of 6-8 weeks old.

Wild animals

No wild animals were used in this study.

Reporting on sex

Male

Field-collected samples

No field-collected sample are used in the study.

Ethics oversight

All studies were approved by the University of Sydney Animal Ethics Committee (Protocol 2021/1912) in accordance with the requirements of the Australian Code of Practice for the Care and Use of Animals for Scientific Purposes.

Note that full information on the approval of the study protocol must also be provided in the manuscript.
