# Supplementary figures and images for: Development of supramolecular anticoagulants with on-demand reversibility
Source: Nat Biotechnol. 2024 Apr 30;43(2):186–93. doi: 10.1038/s41587-024-02209-z (PMC11825364; doi:10.1038/s41587-024-02209-z)

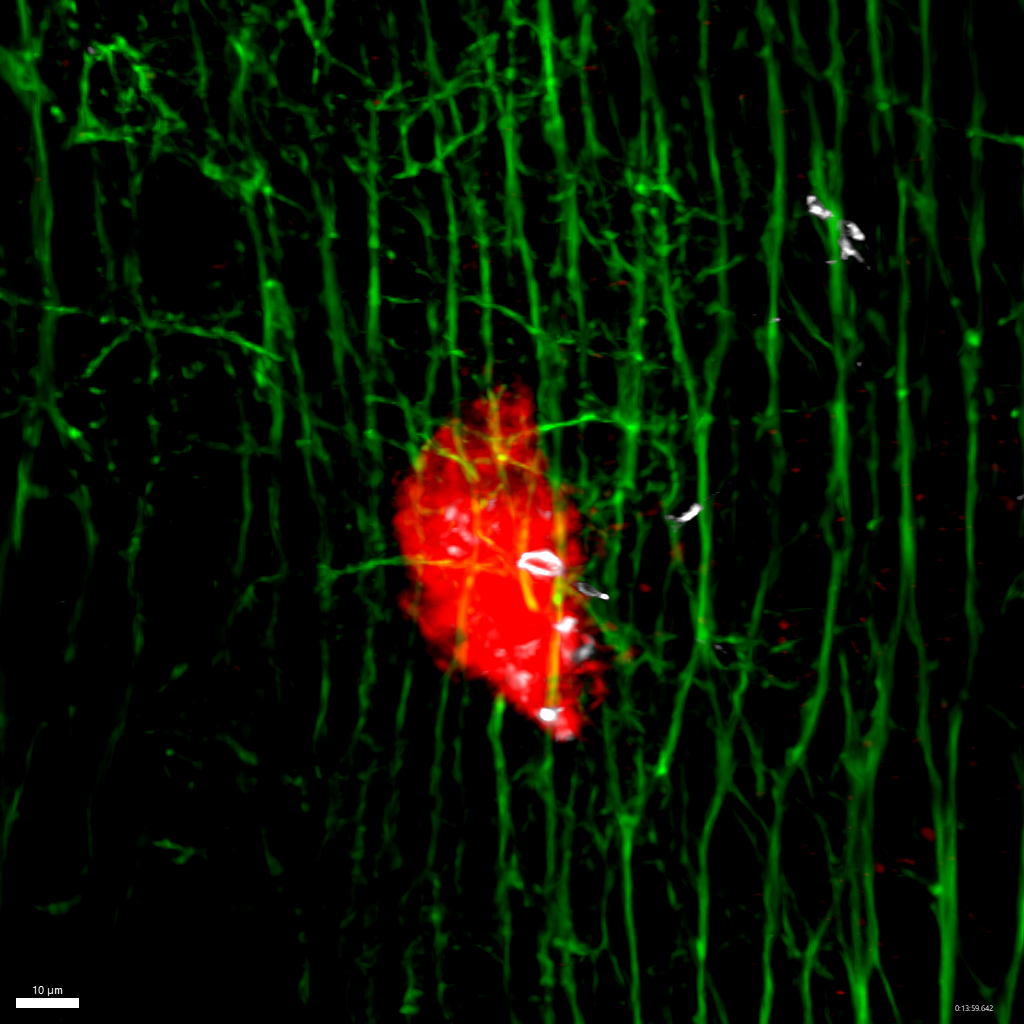

Supplement: Supplementary file 4 — Raw images of maximum intensity projections. [file 41587_2024_2209_MOESM4_ESM.zip › In Vivo Max Projections/A1-E1.tif]

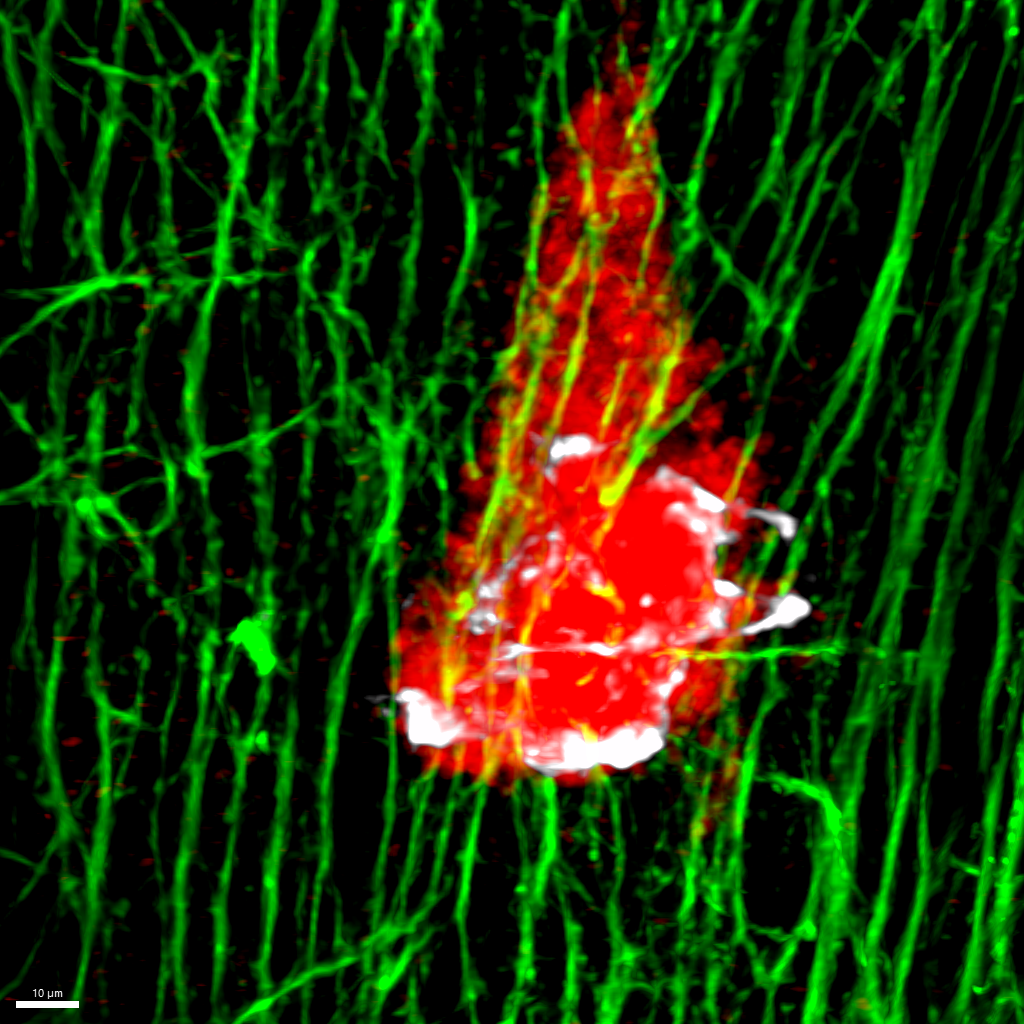

Supplement: Supplementary file 4 — Raw images of maximum intensity projections. [file 41587_2024_2209_MOESM4_ESM.zip › In Vivo Max Projections/A8-E1+AD2.tif]

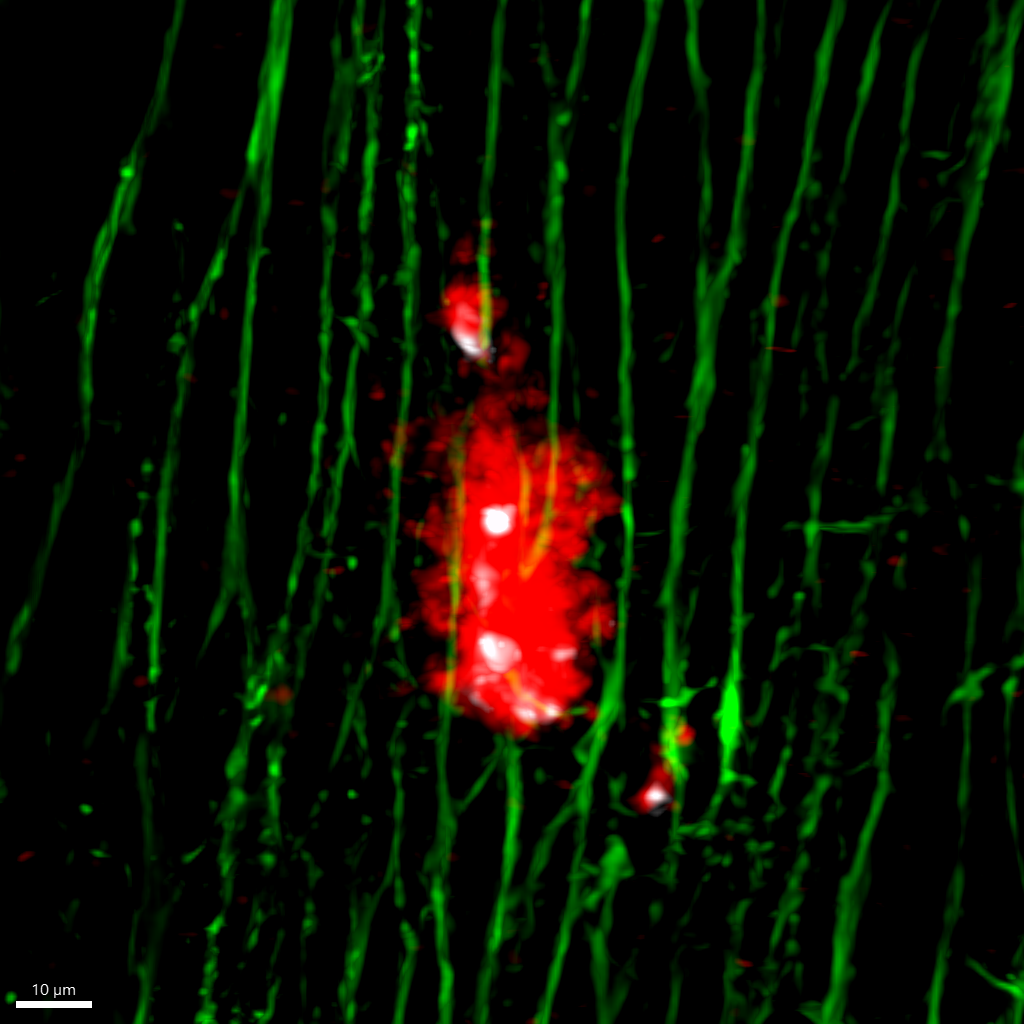

Supplement: Supplementary file 4 — Raw images of maximum intensity projections. [file 41587_2024_2209_MOESM4_ESM.zip › In Vivo Max Projections/A8-E1.tif]

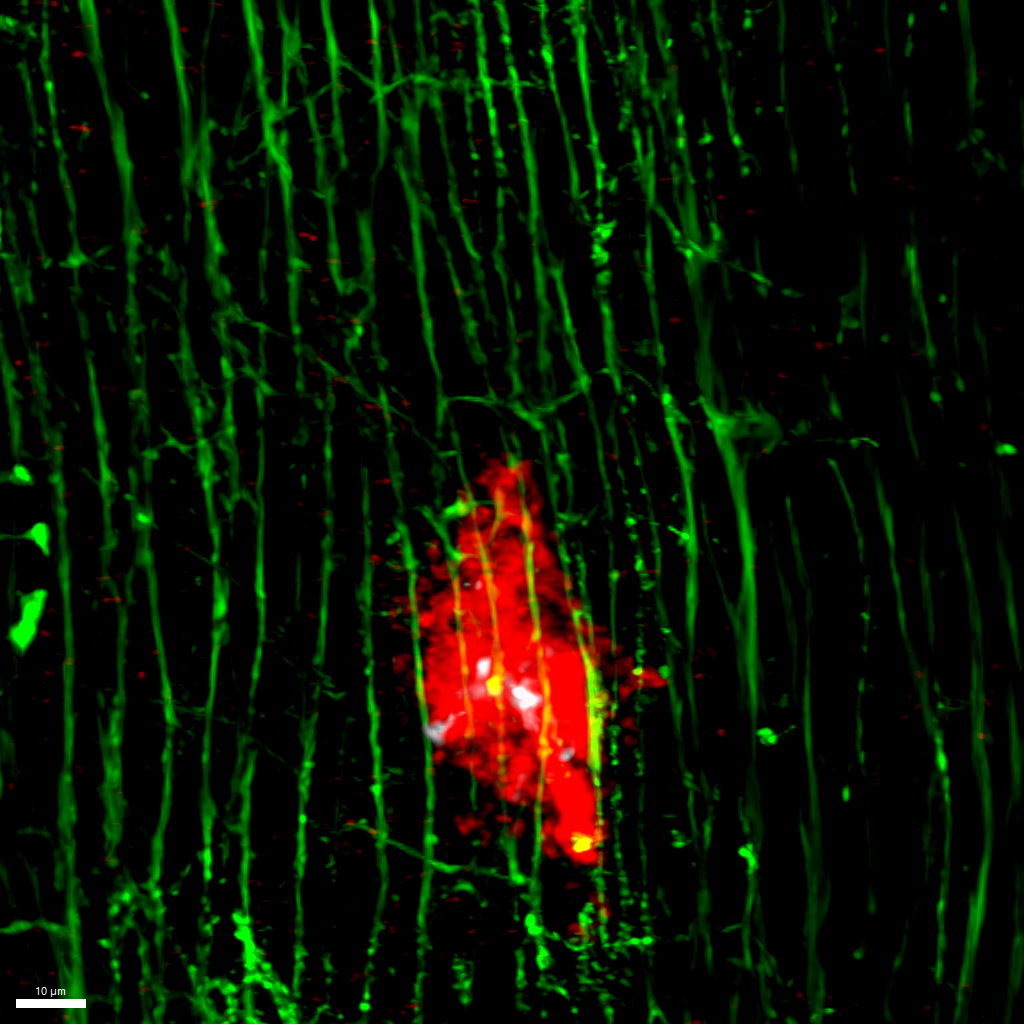

Supplement: Supplementary file 4 — Raw images of maximum intensity projections. [file 41587_2024_2209_MOESM4_ESM.zip › In Vivo Max Projections/Argatroban.tif]

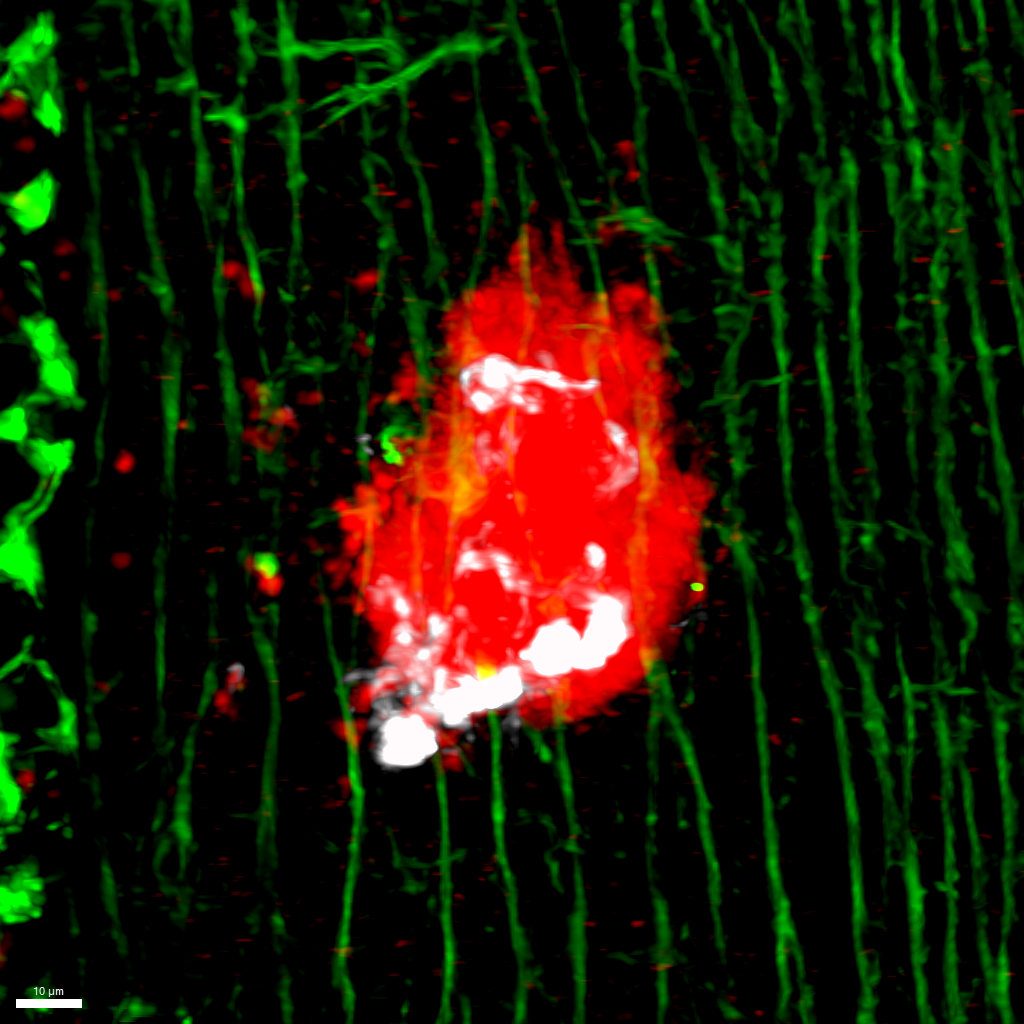

Supplement: Supplementary file 4 — Raw images of maximum intensity projections. [file 41587_2024_2209_MOESM4_ESM.zip › In Vivo Max Projections/No Inhibitor.tif]
